# Microbial inoculants modulate the rhizosphere microbiome, alleviate plant stress responses, and enhance maize growth at field scale

**DOI:** 10.1186/s13059-025-03621-7

**Published:** 2025-06-01

**Authors:** Davide Francioli, Ioannis D. Kampouris, Theresa Kuhl-Nagel, Doreen Babin, Loreen Sommermann, Jan H. Behr, Soumitra Paul Chowdhury, Rita Zrenner, Narges Moradtalab, Michael Schloter, Joerg Geistlinger, Uwe Ludewig, Günter Neumann, Kornelia Smalla, Rita Grosch

**Affiliations:** 1 Department of Nutritional Crop Physiology, Institute of Crop Science, University of Hohenheim, Stuttgart, Germany; 2 Department of Soil Science and Plant Nutrition, Hochschule Geisenheim University, Geisenheim, Germany; 3 Institute for Epidemiology and Pathogen Diagnostics, Julius Kühn Institute (JKI)—Federal Research Centre for Cultivated Plants, Braunschweig, Germany; 4 Plant-Microbe Systems, Leibniz Institute of Vegetable and Ornamental Crops (IGZ), Großbeeren, Germany; 5 Department of Agriculture, Ecotrophology and Landscape Development, Anhalt University of Applied Sciences, Bernburg, Germany; 6 Helmholtz Zentrum München—German Research Center for Environmental Health, Neuherberg, Germany

## Abstract

**Background:**

Field inoculation of crops with beneficial microbes is a promising sustainable strategy to enhance plant fitness and nutrient acquisition. However, effectiveness can vary due to environmental factors, microbial competition, and methodological challenges, while their precise modes of action remain uncertain. This underscores the need for further research to optimize inoculation strategies for consistent agricultural benefits.

**Results:**

Using a comprehensive, multidisciplinary approach, we investigate the effects of a consortium of beneficial microbes (BMc) (*Pseudomonas* sp. RU47, *Bacillus atrophaeus* ABi03, *Trichoderma harzianum* OMG16) on maize (*Zea mays* cv. Benedictio) through an inoculation experiment conducted within a long-term field trial across intensive and extensive farming practices. Additionally, an unexpected early drought stress emerged as a climatic variable, offering further insight into the effectiveness of the microbial consortium. Our findings demonstrate that BMc root inoculation primarily enhanced plant growth and fitness, particularly by increasing iron uptake, which is crucial for drought adaptation. Inoculated maize plants show improved shoot growth and fitness compared to non-inoculated plants, regardless of farming practices. Specifically, BMc modulate plant hormonal balance, enhance the detoxification of reactive oxygen species, and increase root exudation of iron-chelating metabolites. Amplicon sequencing reveals shifts in rhizosphere bacterial and fungal communities mediated by the consortium. Metagenomic shotgun sequencing indicates enrichment of genes related to antimicrobial lipopeptides and siderophores.

**Conclusions:**

Our findings highlight the multifaceted benefits of BMc inoculation on plant fitness, significantly influencing metabolism, stress responses, and the rhizosphere microbiome. These improvements are crucial for advancing sustainable agricultural practices by enhancing plant resilience and productivity.

**Supplementary Information:**

The online version contains supplementary material available at 10.1186/s13059-025-03621-7.

## Background

Modern agriculture relies on intensive tillage, fertilization, and pesticide applications to achieve high crop yields. However, these practices carry potential for environmental risks, including promotion of soil erosion, ecosystem eutrophication, biodiversity decline, and impairment of soil integrity [1–3]. The implementation of sustainable farming strategies to maintain agro-ecosystem functions and services has been proposed to address these concerns. The integration of reduced tillage and fertilization intensity, while considering the beneficial interaction between plants and rhizosphere microorganisms, has potential to stabilize crop yield, plant fitness and resilience, as well as soil health and fertility [4–7]. Indeed, such practices also entail risks for nutrient limitation or increased pathogen/pest pressure that may, due to still limited sustainable options, require conventional agricultural measures [8–10]. Considering the positive effects of beneficial microorganisms (BMs) on plant performance [11–15], their use in farming offers a chance to exploit the interplay between plants and beneficial rhizosphere microbes [16–18], which also have the potential to alleviate plant responses to biotic and abiotic stresses [19–22]. Host plants typically recruit beneficial microorganisms primarily through root exudates that generally serve as chemo-attractants or repellants and deliver nutrients that facilitate microbial rhizosphere colonization. However, the efficacy of BM inoculants at field scale was often inconsistent [23–25]. This remains a major challenge for successful agricultural application. Insufficient root colonization (either root surface, intercellular space or intracellular) and unfavorable environmental conditions are major factors that may hinder the interaction between crops and BMs [26, 27]. For optimal rhizosphere competence, inoculated BMs must compete with the indigenous soil microbial community and adapt to diverse and occasionally harsh environmental conditions.

BMs often show synergisms of their potential functions in the rhizosphere with enhanced promotion of plant performance. Consequently, the application of BM consortia (BMc) was recognized as an opportunity to exploit such synergisms. Especially *Bacillus* and *Pseudomonas* strains with plant-beneficial activity often show synergisms, and their combined inoculation can promote plant growth [28]. In addition, *Trichoderma* strains can interact positively with *Bacillus* strains or show synergistic effects upon co-inoculation despite belonging to different kingdoms [29]. In a previous field experiment, we investigated the ability of a BM consortium (BMc) to establish in the winter rye rhizosphere and assessed its impact on plant performance under various farming practices [13]. Composed of *Pseudomonas* sp. (RU47), *Bacillus atrophaeus* (ABi03), and *Trichoderma harzianum* (OMG16), the BMc demonstrated a high capacity for winter rye rhizosphere colonization. These microorganisms persisted through winter dormancy and significantly enhanced plant nutrient status and performance, particularly under organic farming conditions, which provide less nutrients to the plants than intensive conventional fertilization. Reflecting a broad host range, plant growth-promoting properties of the selected strains have additionally been demonstrated for various crops including lettuce [30, 31], oilseed rape [32], tomato [33], and maize [34]. Combining different microbial strains as BM consortia (BMc) with complementary properties is discussed as an approach to increase the efficiency and flexibility of BM-based production strategies under variable environmental conditions [35]. For example, *Trichoderma-Bacillus* combinations are well-documented examples in this context [36, 37]. Consequently, the use of BMc for sustainable farming, particularly emphasizing reduced agrochemical input and tillage, may be crucial for improving crop health and yield stability.

Seeking to comprehensively examine the impact of the above-mentioned BMc on plant performance and to test the hypothesis that BMc root inoculation mitigates growth limitation associated with reduced N-fertilization intensity, we conducted a systematic inoculation trial on maize (*Zea mays* cv. Benedictio) within a long-term field experiment (LTE) established in 1992. The multi-purpose crop maize was chosen as model plant due to its diverse and dynamic role in global agri-food systems and food/nutrition security [38]. The used LTE employs intensive (recommended N-fertilization intensity and pesticide application) and extensive (reduced N-fertilization intensity without fungicide application) farming practice within a crop rotation (wheat, maize, wheat, rapeseed, barley) using cultivator tillage. To rigorously assess the impact of BMc root inoculation on maize performance, we evaluated plant traits (growth, physiological stress indicators, phytohormonal profiles, and nutrient status), as well as maize root and rhizosphere metabolites. Additionally, we investigated whether BMc inoculation induced structural and functional changes within rhizosphere bacterial and fungal communities, utilizing amplicon and metagenome shotgun sequencing. Severe drought conditions during the experimental year, especially in the juvenile maize establishment phase, provided a unique opportunity to investigate the BMc effects under substantial abiotic stress. This pioneering study represents the first field investigation of the impact of BMc inoculation on the soil/microbe/plant system under contrasting mineral N-fertilization intensities, employing a multidisciplinary framework encompassing diverse analytical techniques.

## Results

### Description of the experimental design

An inoculation experiment was conducted within a long-term field trial (LTE) to investigate the effects of a consortium of beneficial microorganisms (BMc; *Pseudomonas* sp. RU47, *Bacillus atrophaeus* ABi03, and *Trichoderma harzianum* OMG16) on maize performance (*Zea mays* cv. Benedictio). The experimental plots employed conservation tillage and received either the recommended use of nitrogen (N) and pesticides for maize (intensive, Int) or reduced N use and no fungicide application (extensive, Ext). Maize roots were drenched twice with the BMc at growth stages EC 12 and 14–16, and the plants were harvested at maturity (EC 53–63). During the experiment duration, spring precipitation was exceptionally low compared to the average spring rainfall of the past 30 years, with almost no rainfall in April and less in May and July (Additional file 1: Fig. S1). To elucidate the effects of the BMc on the maize-microbiome interaction in the rhizosphere, we analyzed various parameters, including root traits (total root length, root hair length), shoot dry mass (SDM), physiological stress indicators, stress-related gene transcripts in leaves, phytohormones, nutrient status, root and rhizosphere metabolites, and bacterial/archaeal and fungal community compositions, as well as rhizosphere metagenome profiles.

### BMc strains colonized the maize rhizosphere and increased shoot dry mass and iron uptake

**Fig. 1:**
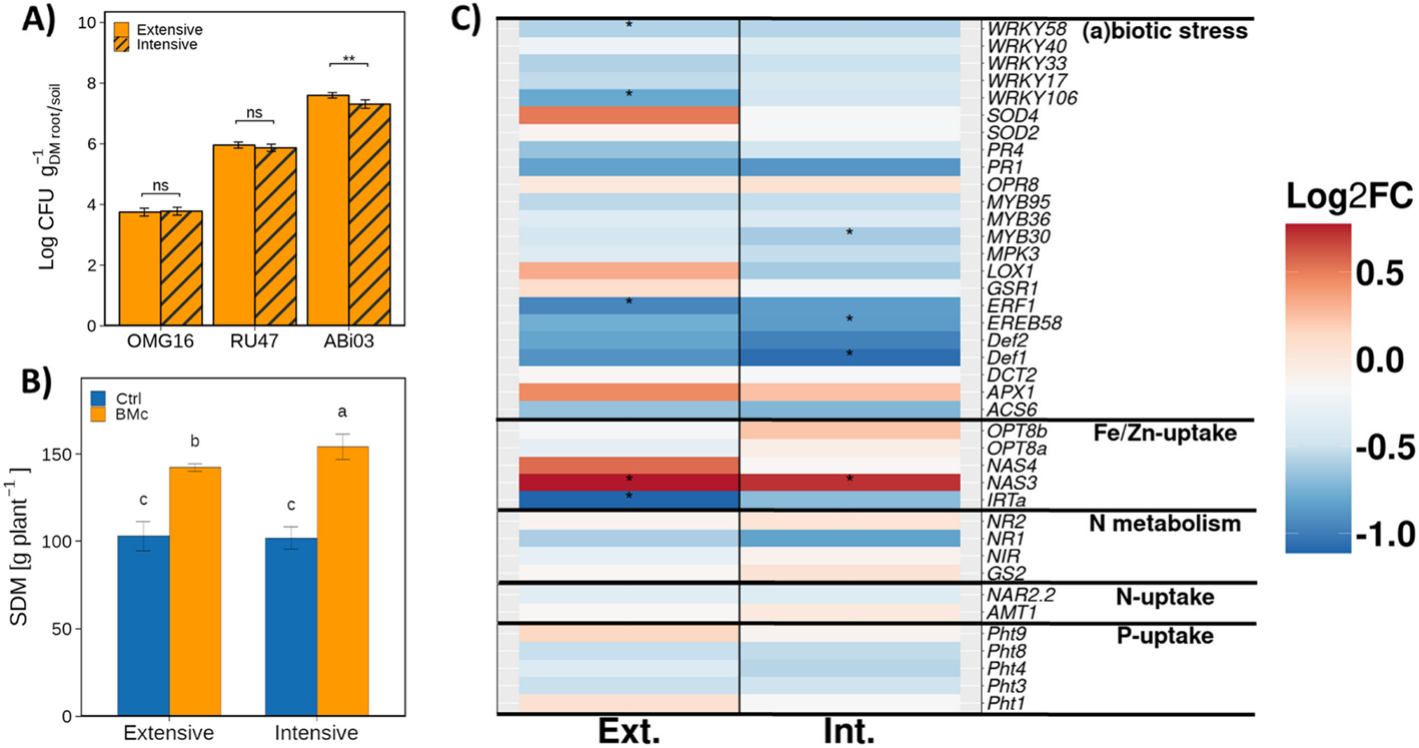
**A** The rhizosphere competence (estimated with culture-dependent methods) of the BMc inoculants (*Trichoderma harzianum* OMG16, *Pseudomonas* RU47, and *Bacillus atrophaeus* ABi03) in the maize rhizosphere (*t*-test, *p < 0.05, **p < 0.01, ns—not significant, *n* = 4). Natural background of rifampicin resistance in the rhizosphere was examined through plating as well: no rifampicin-resistant colonies were found in the control samples. **B** Shoot dry mass (SDM) of maize (*Zea mays* cv. Benedictio) grown in intensively or extensively fertilized soils with (BMc) or without (Ctrl) inoculation. Different letters indicate significant differences at *p* < 0.05 according to Tukey’s HSD post hoc test (Benjamini–Hochberg correction, *n* = 4). **C** The relative transcript levels as inferred by reverse transcription qPCR (ΔCq = housekeeping genes Cq − target gene Cq). The differential abundance was computed and significant differences between BMc-treated and Ctrl plants were verified with Student’s *t*-test and labeled with asterisks (*p ≤ 0.05, *n* = 16). Log_2_FC = log_2_ fold change of relative gene expression (BMc ΔCq − Control ΔCq). Detailed gene assignments can be found in Table S3

The inoculated strains effectively colonized the rhizosphere, as determined by CFU counts through rifampicin selective plating (Fig. 1A), while no rifampicin-resistant colonies were detected in the rhizosphere of the control plants. Among the BMc strains, ABi03 exhibited the most efficient colonization with a density two orders of magnitude higher than RU47 and OMG (ANOVA, *F* = 1889.68, *p* < 0.001, *n* = 8; Fig. 1A). N-fertilization intensity influenced the ABi03 rhizosphere competence (ANOVA, *F* = 16.45, *p* = 0.013, *n* = 8; Fig. 1A), but had no significant effect on RU47 and OMG16 (ANOVA, *F*_RU47_ = 1.2, *F*_OMG16_ = 0.8, *p* > 0.05, *n* = 8; Fig. 1A). Maize SDM was significantly higher by approximately 30% in BMc inoculated plants compared to non-inoculated controls (ANOVA, *F* = 149.7, *p* < 0.001, *n* = 8; Fig. 1B). The increase in SDM was accompanied by higher concentrations of sulfur, iron, zinc, and manganese in maize leaves of inoculated plants (two-way ANOVA, *F* = 0.23–17.14, *p* < 0.05, *n* = 8; Table 1 and Additional file 2: Table S1). However, only iron concentration showed an increase regardless of N-fertilization intensity (two-way ANOVA, *F* = 17.14, Tukey’s HSD, *p* < 0.05, Table 1). Moreover, none of the nutrients (except N in extensively fertilized control plants) showed concentrations below the deficiency limit (Table 1). In contrast, we did not observe any significant effects of the experimental variables on most of the measured soil chemical parameters (Additional file 2: Table S2). Furthermore, BMc inoculation did not significantly affect root growth (total root and hair length) or arbuscular mycorrhizal fungi (AMF) colonization, processes crucial for the spatial acquisition of mineral nutrients (Additional file 1: Fig. S2). However, AMF root colonization was significantly reduced under intensive N-fertilization, indicating that high N-fertilization and pesticides negatively affect mycorrhizal symbiosis.

**Table 1:** Macro- and micronutrient concentrations measured in maize shoots (*Zea mays* cv. Benedictio) grown under extensive and intensive N-fertilization intensities, without (Ctrl; control plants) and with BMc (beneficial microorganism consortium) inoculation. DT indicates deficiency threshold based on Campbell (2000). Data represent mean values ± standard deviation. Different letters indicate significant differences (*p* < 0.05, Tukey’s HSD test, Benjamini–Hochberg correction, *n* = 4)

	**DT**	**Extensive**		**Intensive**	
		**Ctrl**	**BMc**	**Ctrl**	**BMc**
Macronutrients (g kg^−1^)					
C		453 ± 0	454 ± 3.00	453 ± 1.00	454 ± 3.00
N	30	29.7 ± 0.30	31.0 ± 0.80	31.1 ± 1.40	31.8 ± 1.20
P	2.5	2.72 ± 0.16	2.83 ± 0.14	2.57 ± 0.10	2.59 ± 0.13
K	20	22.1 ± 0.80	22.6 ± 1.50	22.0 ± 0.90	21.1 ± 1.00
Mg	2.5	1.84 ± 0.07a	1.85 ± 0.05a	1.72 ± 0.07ab	1.63 ± 0.09b
Ca	4	5.00 ± 0.36	5.03 ± 0.32	4.83 ± 0.20	5.05 ± 0.72
S	1.2	1.95 ± 0.06b	2.13 ± 0.10ab	2.14 ± 0.08ab	2.23 ± 0.07a
Micronutrients (mg kg^−1^)					
Fe	15	85.0 ± 5.0bc	96.2 ± 4.70a	80.2 ± 5.70c	91.7 ± 6.30ab
Cu	5	8.78 ± 0.32	8.41 ± 0.48	10.07 ± 1.01	9.29 ± 0.77
Zn	15	37.53.3b	40.8 ± 4.50ab	40.9 ± 3.90ab	47.0 ± 3.60a
Mn	15	59.1 ± 5.0b	61.7 ± 6.20ab	62.4 ± 4.60ab	77.3 ± 16.4a

Campbell CR (ed). Reference Sufficiency Ranges for Plant Analysis in the Southern Region of the United States. Southern Cooperative Series Bulletin No. 2000, 394

In response to BMc inoculation, a substantial number of stress-related and nutrient uptake-related maize genes, based on relative transcript abundances measured with reverse transcription qPCR, tended to be repressed in the leaves, regardless of the N-fertilization level (Fig. 1C). In contrast, stress-related genes encoding for iron-dependent enzymes (Additional file 2: Table S3), involved in detoxification of reactive oxygen species (ROS), such as superoxide dismutase (SOD) and ascorbate peroxidase (APX) activity, and genes associated with nutrient acquisition pathways, exhibited distinct upregulation in response to BMc inoculation, particularly those involved in iron uptake. Specifically, *NAS3*, a gene associated with the synthesis of the iron/zinc chelator nicotianamine (Additional file 2: Table S3), which likely contributed to enhanced iron concentrations in leaves, was consistently upregulated in extensively and intensively fertilized BMc inoculated plants (*t*-test, *p* < 0.05, *n* = 4; Fig. 1C). The iron transporter *IRTa* showed a contrasting pattern, albeit statistically significant only in the context of extensive N-fertilization samples (*t*-test, *p* = 0.049, *n* = 4).

### BMc inoculation exerted systemic effects on maize hormonal status and improved stress resilience independent of N-fertilization intensity

**Fig. 2:**
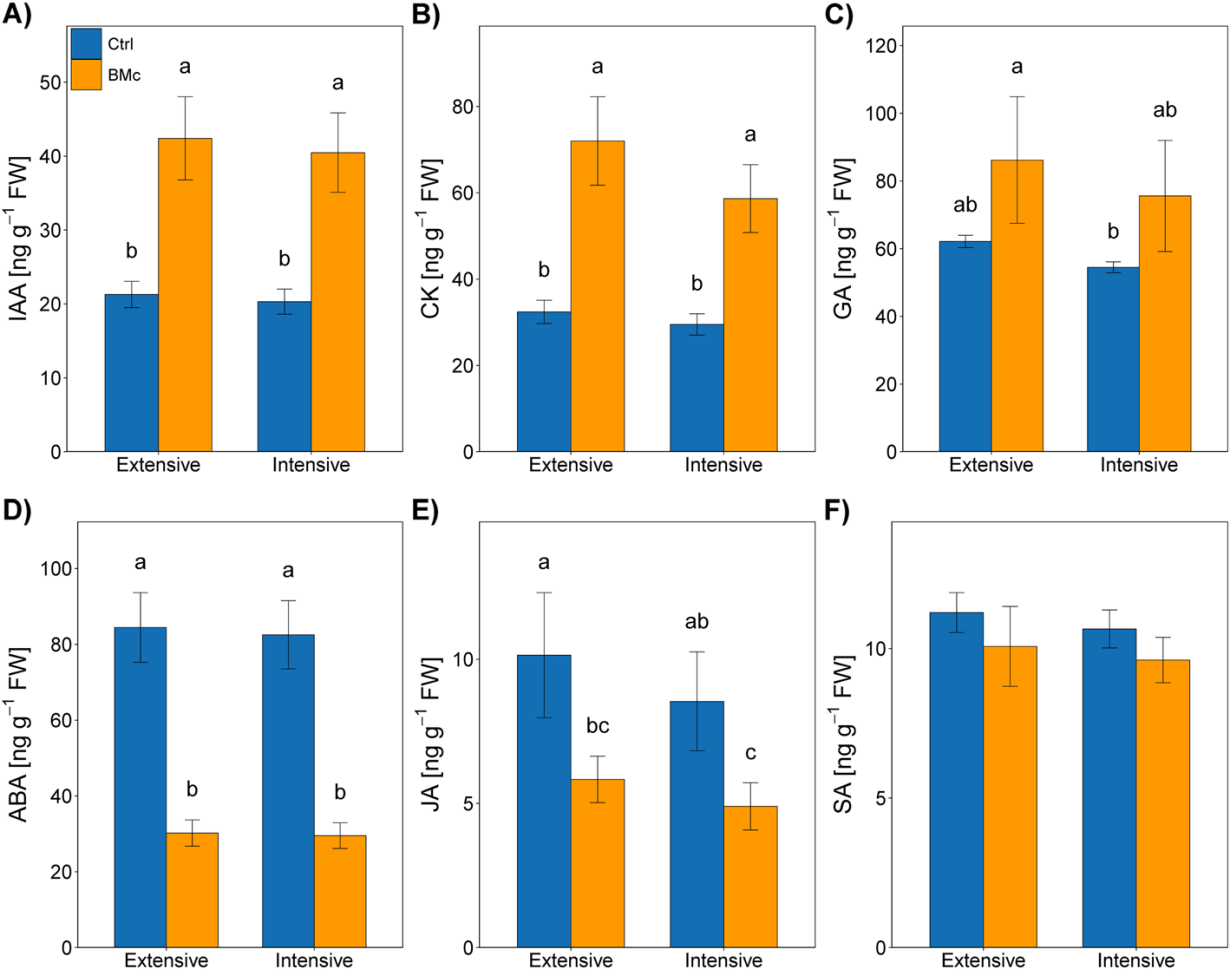
Plant hormone concentrations measured in maize shoots (*Zea mays* cv. Benedictio) grown under different N-fertilization intensities without (Ctrl, control) or with BMc (beneficial microorganism consortium) inoculation: **A** indole acetic acid, **B** zeatin (cytokinin), **C** gibberellic acid, **D** abscisic acid, **E** jasmonic acid, and **F** salicylic acid. Data represent mean values of four biological replicates ± standard deviation. Different letters indicate significant differences (*p* < 0.05) according to Tukey’s HSD test (Benjamini–Hochberg correction, *n* = 4). FW fresh weight

BMc inoculation significantly increased the concentration of growth-promoting phytohormones (indole acetic acid, gibberellic acid, and zeatin) in the shoots by approximately twofold, while decreasing the concentrations of stress-related hormones (abscisic acid (ABA) and jasmonic acid (JA)) (two-way ANOVA, *F*_ABA_ = 28.20, *F*_JA_ = 279.34, *p* < 0.05, Fig. 2). Salicylic acid (SA) concentrations in shoots remained unaffected by BMc inoculation (Fig. 2). In the roots, a similar response of plant growth hormones to BMc inoculation was observed, with significantly higher concentrations of indole acetic acid (IAA) and zeatin (cytokinin) in the inoculated plants compared to the control (Additional file 1: Fig. S3). Interestingly, BMc inoculation led to a significant increase in the concentration of stress-related hormones involved in plant defense in the roots (two-way ANOVA, *F*_JA_ = 94.64, *F*_SA_ = 229.51, *p* < 0.001, Additional file 1: Fig. S3), which could potentially be associated with the plant response to the presence of a high microbial load or the drastic change in microbial community composition in the maize rhizosphere.

**Fig. 3:**
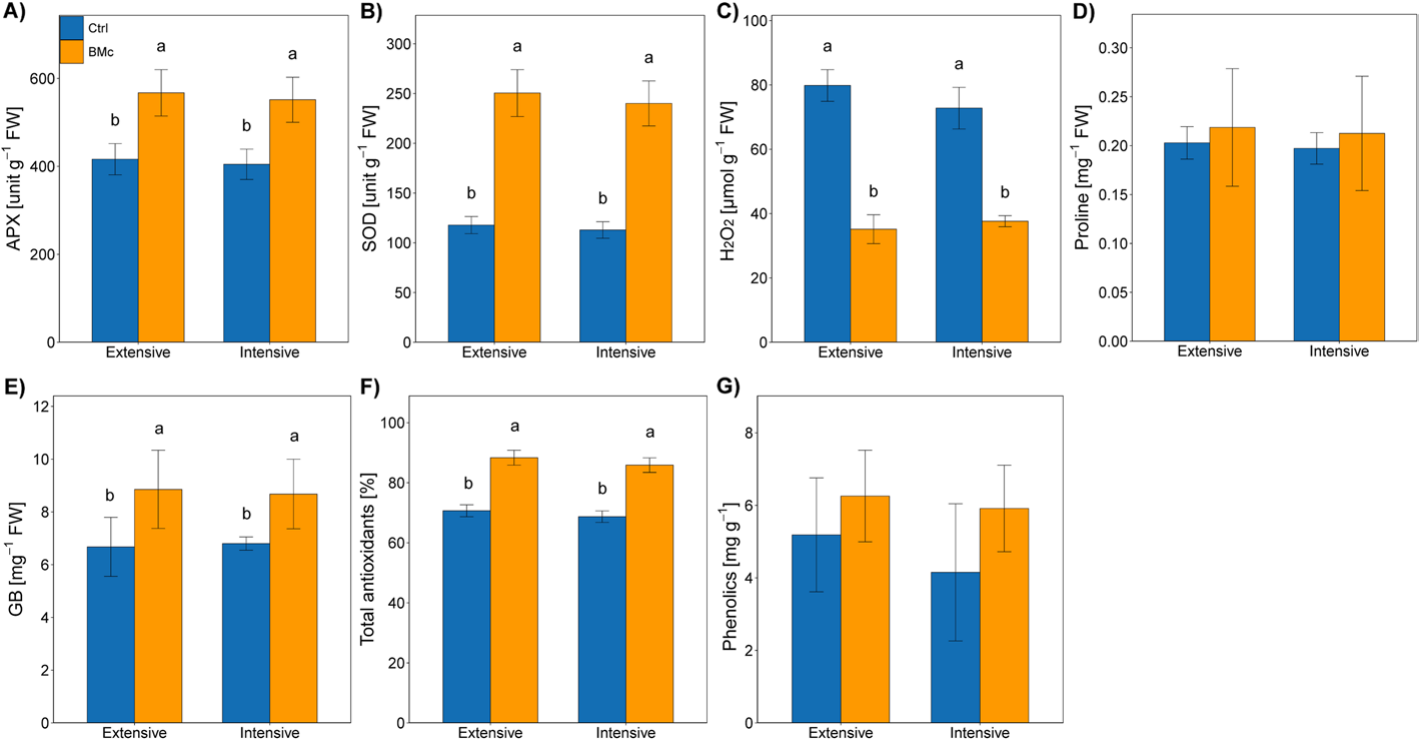
Metabolic stress indicators: activity of ROS (reactive oxygen species) scavenging key enzymes and concentrations of related stress metabolites in maize leaves (*Zea mays* cv. Benedictio) under different N-fertilization intensities with BMc (beneficial microorganism consortium) or without inoculation (Ctrl, control): **A** ascorbate peroxidase activity, **B** superoxide dismutase activity, **C** H_2_O_2_ concentration, **D** proline concentration, **E** glycine betaine concentration, **F** total antioxidant activity, and **G** total phenolics concentration. Different letters indicate significant differences at *p* < 0.05 according to Tukey’s HSD test (Benjamini–Hochberg correction, *n* = 4). FW fresh weight

Since BMc inoculation enhanced the gene expression and shoot activities of iron-dependent ROS detoxification (APX and SOD, ANOVA, *F* = 45.09, *p* < 0.05, Fig. 3), consequently it halved the accumulation of hydrogen peroxide (H_2_O_2_) in the leaf tissue (ANOVA, *F* = 285.5, *p* < 0.001, Fig. 3), and additionally increased leaf concentrations of total antioxidant and glycine betaine (ANOVA, *F* = 12.54, *p* < 0.05, Fig. 3), which have functions in non-enzymatic ROS detoxification and osmotic adjustment. The observed decrease in stress-resilience markers within leaf tissues of BMc inoculated maize plants aligns with the downregulation of stress-related genes in this tissue, indicating a plausible involvement of BMc inoculation in regulating plant stress responses. Principal component analysis (PCA) (Additional file 1: Fig. S4) of the aforementioned variables further mirrors and supports the effects of BMc inoculation on stress mitigation, while promoting maize growth and increasing nutrient uptake.

### The patterns of rhizosphere metabolites are shaped by BMc inoculation

**Fig. 4:**
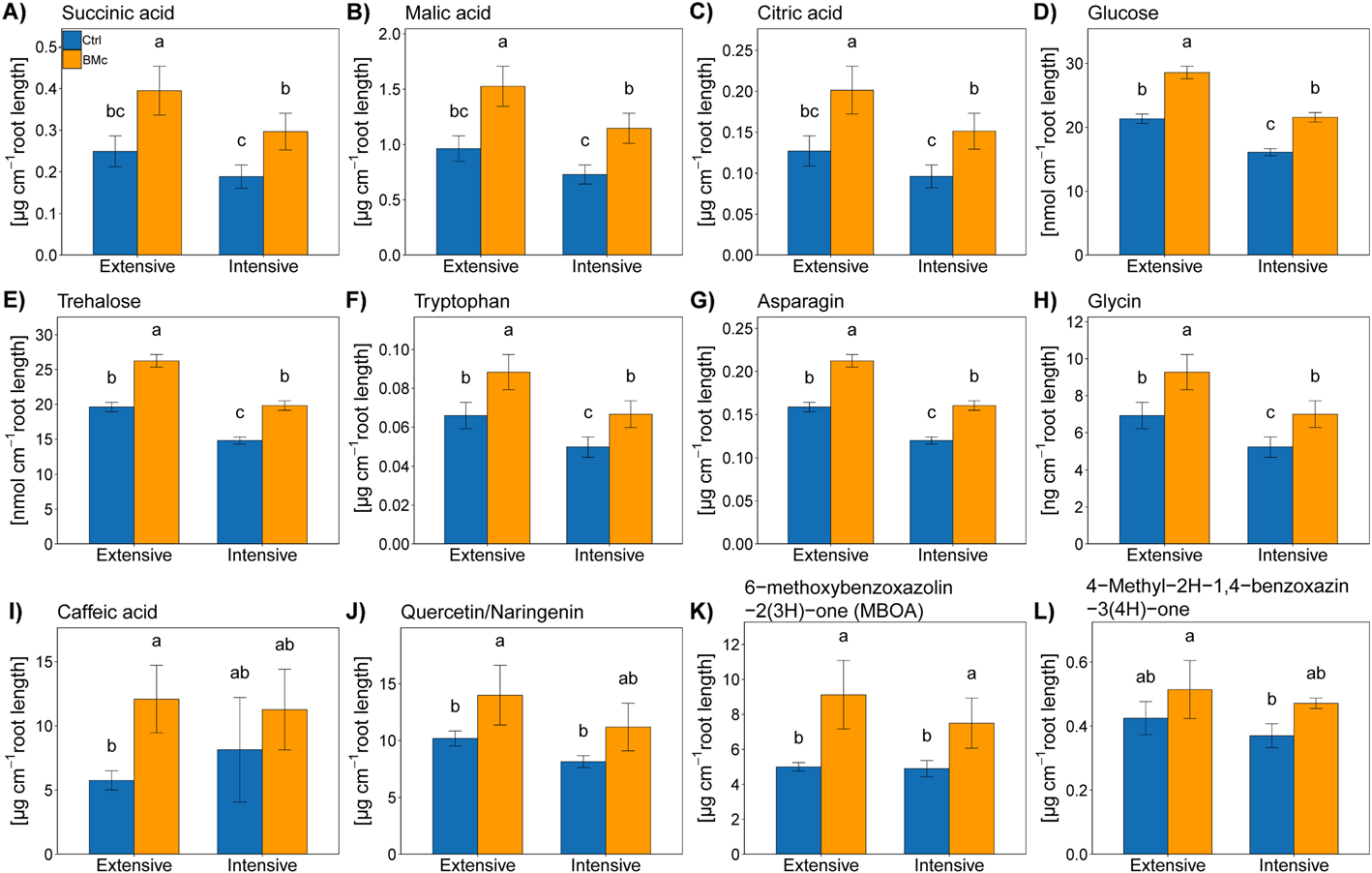
Root exudate metabolites detected at the root apical zone of maize (*Zea mays* cv. Benedictio) grown under different N-fertilization intensities with BMc (beneficial microorganism consortium) or without inoculation (Ctrl, control). Data represent mean values of four biological replicates ± standard deviation. Different letters indicate significant differences at *p* < 0.05 according to Tukey’s HSD test (Benjamini–Hochberg correction, *p* < 0.05, *n* = 4)

To evaluate the impact of BMc inoculation on rhizosphere metabolites, we established experimental plots with root observation windows that enabled the sampling and profiling of low-molecular weight metabolites across distinct maize root zones (Additional file 1: Fig. S5). As expected, metabolite concentrations exhibited significant variation across the sampled root zones, reflecting the distinct physiological and functional characteristics of different root types (Additional file 1: Fig. S6, Additional file 2: Table S4). Given the higher detection of secondary metabolites in the apical root zone, likely exerting a significant effect on microbiota assembly around maize roots in contrast to other root types, our investigation specifically targeted the analysis of rhizosphere metabolites from the apical root zone (Additional file 1: Fig. S6). In line with the improved growth performance exhibited by BMc inoculated plants, a majority of the identified metabolites showed significant increases following BMc inoculation, irrespective of N-fertilization intensity (*p* < 0.05, Tukey’s HSD test, Fig. 4). These enhancements encompassed organic acid anions (predominantly malate), sugars (such as glucose and trehalose), specific phenolic acids and flavonoids (like caffeic acid, quercetin/naringenin), as well as benzoxazinoid metabolites with antibiotic, allelopathic, and iron-mobilizing properties (e.g., MBOA) (Fig. 4).

### BMc inoculation increased Myxococcota, Bacteroidota, and Proteobacteria in the maize rhizosphere

**Fig. 5:**
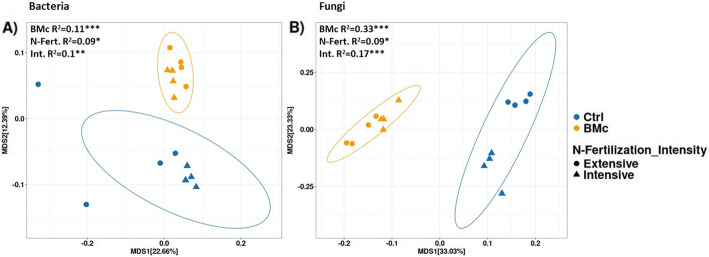
Bacterial (**A**) and fungal community composition (**B**) based on Bray–Curtis dissimilarity in the rhizosphere of maize (*Zea mays* cv. Benedictio). Significant differences were tested with PERMANOVA: *p < 0.05, **p < 0.01, ***p < 0.001, *n* = 4. BMc beneficial microorganism consortium, N-Fert. N-fertilization intensity, Int. interaction effect

**Fig. 6:**
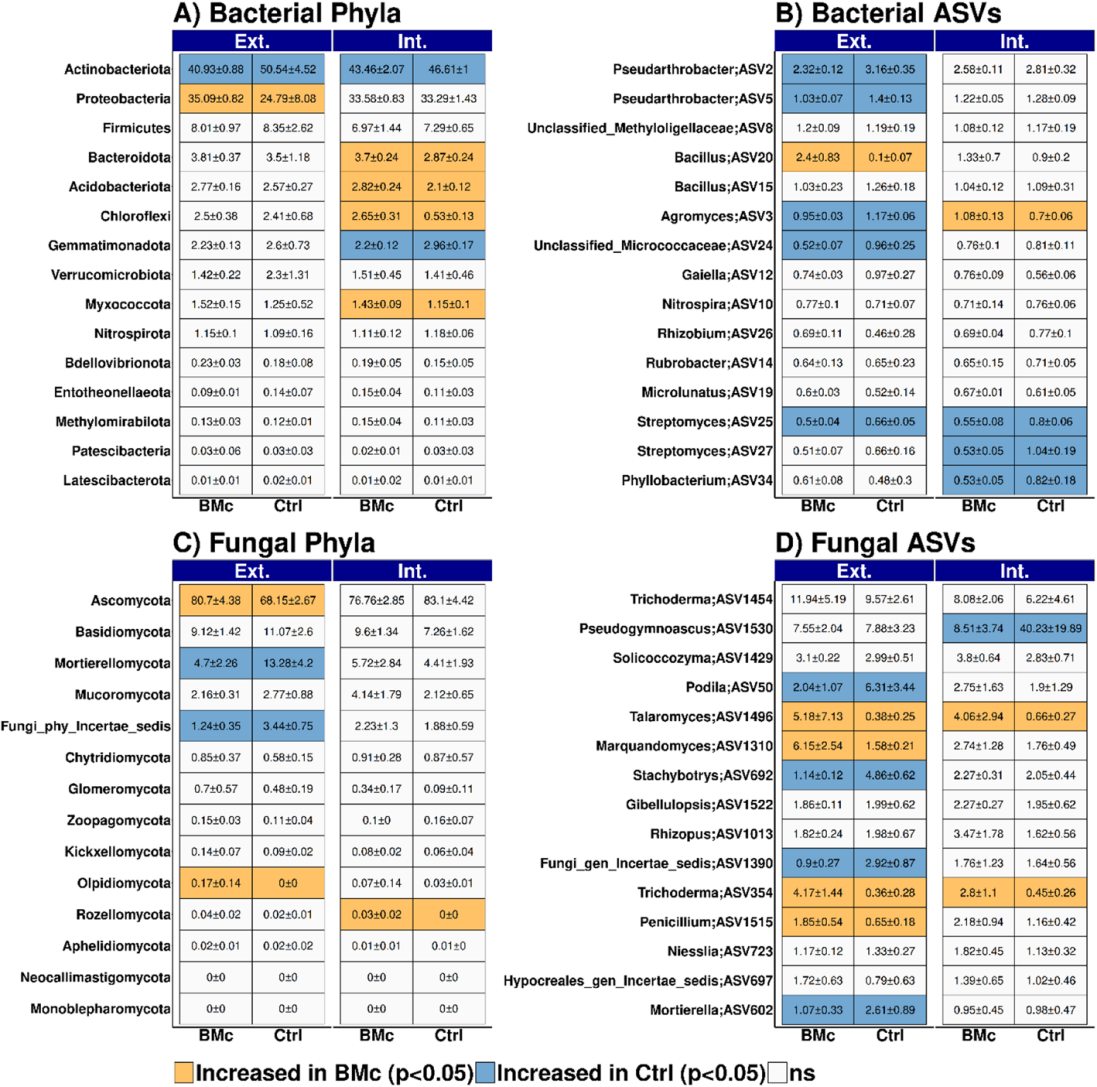
The 15 top taxa for bacterial phyla (**A**), bacterial ASVs (**B**), fungal phyla (**C**), and fungal ASVs (**D**) in the rhizosphere of maize (*Zea mays* cv. Benedictio). For extensive (Ext) and intensive (Int) N-fertilization intensity, respectively, differential abundance of BMc inoculated (beneficial microorganism consortium) vs. Ctrl (control) plants was performed with logistic regression and Benjamini–Hochberg correction (*n* = 4). Taxa with *p* < 0.05 following logistic regression and Benjamini–Hochberg correction are shown in orange and blue color. Numbers indicate the mean relative abundances in four replicates of each group (BMc-Ext., Ctrl-Ext., BMc-Int., and Ctrl-Int.). Values were rounded to the second digit. Ext. extensive, Int. intensive

BMc inoculation significantly impacted bacterial beta-diversity within the maize rhizosphere, suggesting its potential role as a determinant factor shaping bacterial community assembly as revealed by 16S rRNA gene amplicon sequencing (PERMANOVA, BMc: *R*^2^ = 0.11, *p* = 0.001, *n* = 8; Fig. 5A). N-fertilization intensity effect on bacterial community composition was weaker than BMc inoculation (PERMANOVA, *R*^2^ = 0.09, *p* = 0.018, *n* = 8, Fig. 5A) and a significant interaction effect between BMc inoculation and N-fertilization intensity was observed (PERMANOVA *R*^2^ = 0.1, *p* = 0.0028, *n* = 8, Fig. 5A). In addition, both N-fertilization intensity and BMc inoculation influenced alpha-diversity with intensively N-fertilized control plants showing the lowest alpha-diversity and extensively BMc inoculated plants showing the highest alpha-diversity (Additional file 1: Fig. S7 A). However, the results were weak, with only these two groups being statistically different (pairwise Wilcoxon rank-sum test, *p* < 0.05, *n* = 4, Additional file 1: Fig. S7 A). Actinobacteriota, Proteobacteria, and Firmicutes were the most abundant phyla, while Bacteroidota, Acidobacteriota, Gemmatimonadota, Chloroflexi, Myxococcota, and Verrucomicrobiota occurred in relative abundances below 5% (Fig. 6A). BMc inoculation specifically decreased the relative abundance of Actinobacteriota in both N-fertilization intensities, while increasing the relative abundance of Bacteroidota, Acidobacteriodota, Chloroflexi, and Myxococcota under intensive fertilization and Proteobacteria under extensive fertilization (logistic regression, *p* < 0.05, *n* = 4; Fig. 6A). Additionally, BMc inoculation reduced the relative abundance of Gemmatimonadota in the rhizosphere of maize grown under intensive fertilization (logistic regression, *p* < 0.05, *n* = 4; Fig. 6A).

BMc inoculation significantly increased the relative abundance of 55 and 48 ASVs in intensive and extensive fertilization, respectively, out of a total of 6595 bacterial ASVs (logistic regression, *p* < 0.05, Benjamini–Hochberg correction, *n* = 4; Additional file 2: Table S5). Concurrently, the relative abundance of 48 and 33 bacterial ASVs decreased due to BMc inoculation under intensive or extensive N-fertilization, respectively (logistic regression, *p* < 0.05, Benjamini–Hochberg correction, *n* = 4, Additional file 2: Table S5). Out of the 15 most abundant bacterial ASVs identified in the maize rhizosphere, the relative abundance of five of these ASVs, classified as four genera belonging to the Actinobacteriota phylum (*Agromyces*: ASV3, *Pseudarthrobacter*: ASV2 and ASV5, unclassified *Micrococcaceae* ASV24, and *Streptomyces*: ASV27), decreased in response to BMc inoculation under extensive N-fertilization intensity (logistic regression, *p* < 0.05, Benjamini–Hochberg correction, *n* = 4, Fig. 6B). Moreover, the relative abundance of two out of 15 ASVs classified as Actinobacteriota (*Streptomyces*: ASV25 and ASV27) decreased in response to BMc inoculation under intensive N-fertilization (logistic regression, *p* < 0.05, Benjamini–Hochberg correction, *n* = 4, Fig. 6B). In contrast, ASV3 (*Agromyces*) increased in relative abundance due BMc inoculation under intensive N-fertilization intensity (logistic regression, *p* < 0.05, Benjamini–Hochberg correction, *n* = 4, Fig. 6B), an opposite trend than the one observed under extensive N-fertilization intensity (Fig. 6B). Moreover, one ASV (ASV20), classified as *Bacillus* sp., increased due to BMc treatment in samples under extensive N-fertilization (logistic regression,* p* < 0.05, Benjamini–Hochberg correction, *n* = 4; Fig. 6B). Notably, ASV20 exhibited high sequence identity with *Bacillus atrophaeus* and the ABi03 inoculated strain based on pairwise sequence alignment of the amplified region of the 16S rRNA gene (Additional file 1: Fig. S8 A). Furthermore, ASV20 was consistently among the most abundant ASVs detected in the rhizosphere across all samples. Given its high abundance in the control samples (Fig. 6B) and the phylogenetic similarity of ASV20 to other *Bacillus atrophaeus* strains (Additional file 1: Fig. S6 A), we considered ASV20 to represent both the inoculant ABi03 and sequences from naturally occurring *Bacillus* spp. strains. Therefore, we did not exclude ASV20 from the dataset used to assess BMc-driven modulation of the indigenous microbiome. We did not observe ASVs affiliated with *Pseudomonas* (particularly RU47) among the dominant ASVs in the maize rhizosphere of inoculated plants. This result aligns with our culture-dependent data, as RU47 demonstrated significantly lower establishment rates in the rhizosphere compared to ABi03 (Fig. 1A). However, bacterial ASV1083 (classified as *Pseudomonas* as well) shared high sequence homology with RU47, and its relative abundance tended to increase with BMc inoculation (logistic regression, *p* > 0.05, Benjamini–Hochberg correction; *n* = 4, data not shown).

### Fungal community assembly in the rhizosphere was remarkably affected by BMc inoculation

The fungal community composition in the maize rhizosphere was significantly affected by BMc inoculation (PERMANOVA, *R*^2^ = 0.33, *p* < 0.001, *n* = 8, Fig. 5B). Fertilization intensity also influenced fungal community composition, albeit to a lesser extent (PERMANOVA, *R*^2^ = 0.09, *p* = 0.028, *n* = 8, Fig. 5B). A stronger significant interaction effect between BMc inoculation and N-fertilization intensity was found (PERMANOVA, *R*^2^ = 0.17, *p* = 0.001, *n* = 8), when compared to bacterial community composition. This interaction effect was also observed in alpha-diversity, where extensive N-fertilization and BMc inoculation significantly increased richness and the Shannon index compared to control samples under intensive N-fertilization (pairwise Wilcoxon rank-sum test, *p* < 0.05, *n* = 4, Additional file 1: Fig. S7B), similar to the pattern observed in the bacterial community (Additional file 1: Fig. S7 A). Accordingly, both experimental variables, particularly BMc inoculation, significantly affected the composition of the fungal community across all taxonomic levels in the rhizosphere (Fig. 6C and D). For example, at the phylum level, the relative abundance of Ascomycota, the most dominant phylum, increased from 68.2 to 80.7% in extensively fertilized samples due to BMc inoculation (logistic regression, *p* < 0.05, *n* = 4; Fig. 6B). Contrarily, Mortierellomycota and unclassified fungi (Fungi_phy_Incertae) significantly decreased in relative abundance following BMc inoculation under extensive N-fertilization (logistic regression; *p* < 0.05, *n* = 4; Fig. 6C).

Among the 1605 fungal ASVs identified, 121 and 124 exhibited increased relative abundance in response to BMc inoculation under intensive and extensive N-fertilization, respectively, while 49 and 110 ASVs displayed decreased relative abundance under intensive and extensive N-fertilization, respectively (Additional file 2: Table S6). Notably, eight dominant ASVs responded to BMc inoculation in the extensive treatments, while only three ASVs responded in the intensive N-fertilization context. Specifically, four fungal ASVs, affiliated with the genera *Trichoderma*, *Marquandomyces*, *Talaromyces*, and *Penicillium*, were significantly enriched under extensive fertilization (logistic regression, *p* < 0.05, *n* = 4; Fig. 6D), while *Podila*, *Mortierella*, *Stachybotrys*, and Fungi_gen_incertae_sedis showed an opposite trend (logistic regression, *p* < 0.05, *n* = 4; Fig. 6D). Interestingly, BMc inoculation also led to increased abundance of *Trichoderma* and *Talaromyces* ASVs in intensive fertilized treatments (Fig. 6D)*.* However, none of the enriched *Trichoderma* ASVs in response to BMc was closely phylogenetically associated with OMG16, based on the pairwise alignment of the amplified ITS region (Additional file 1: Fig. S8). Moreover, the shifts in abundance of the highly abundant fungal taxa under intensive N-fertilization were restricted to ASV1530 (*Pseudogymnoascus*), which significantly decreased from the explicitly high abundance of 40.23 ± 19.89 to 8.51 ± 3.74% (logistic regression, *p* < 0.05, *n* = 4; Fig. 6D).

### BMc inoculation enhanced relative abundance of microbial lipopeptide and siderophore biosynthesis genes in the maize rhizosphere

**Fig. 7:**
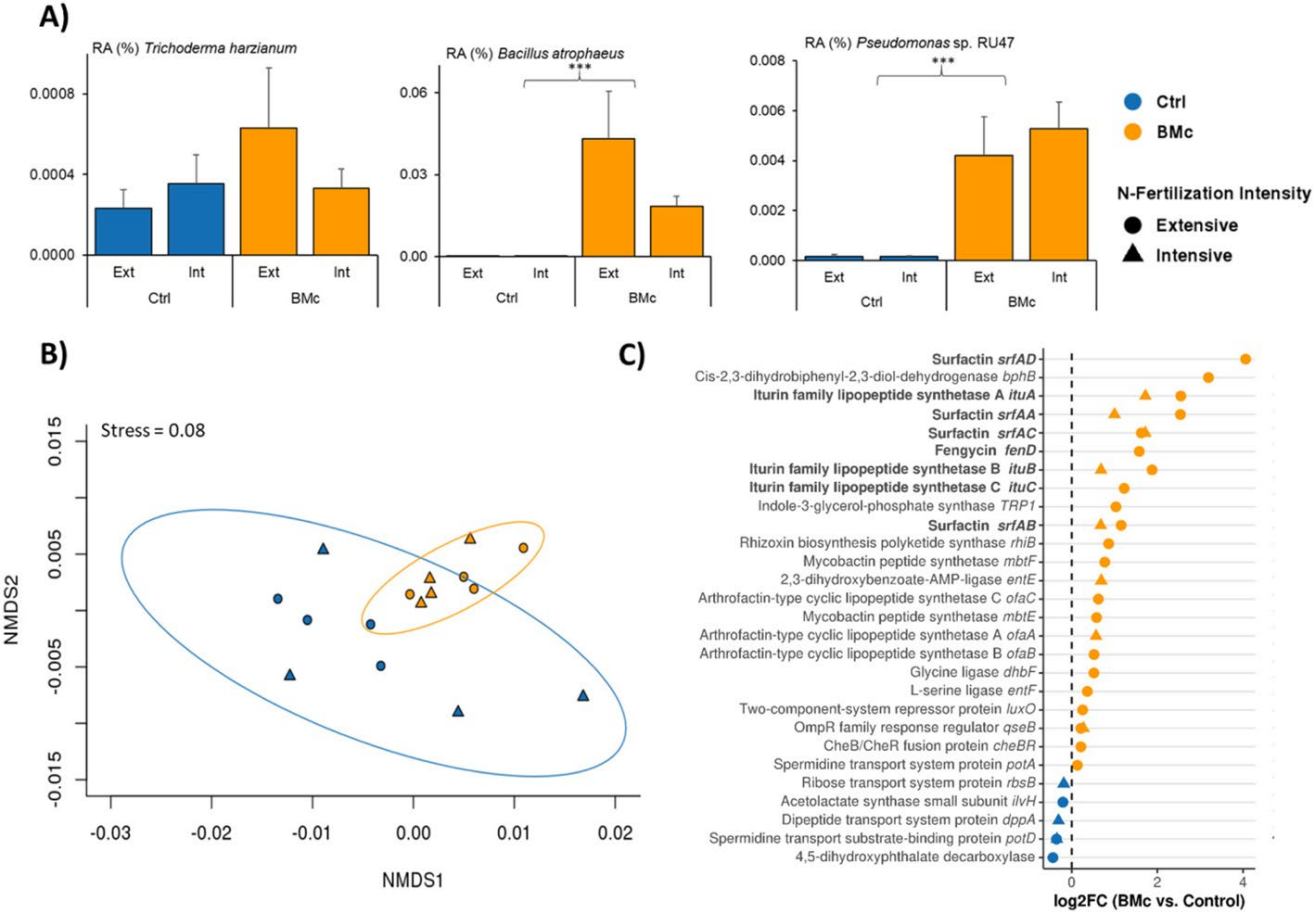
**A** Mapping of reads related to the BMc (beneficial microorganism consortium) strains in the rhizosphere metagenome of maize (*Zea mays* cv. Benedictio). The mapping verified the observed results from plating and amplicon sequencing. Significant differences were tested via two-way ANOVA (*p < 0.05, **p < 0.01, ***p < 0.001, *n* = 4). **B** The profile of functional genes (annotated with COG database) associated with bacteria in the rhizosphere metagenome of maize plants inoculated with BMc vs. Ctrl (control) plants. BMc inoculation significantly affected the functional gene profile (PERMANOVA, *R*^2^ = 0.15, *p* = 0.0014, *n* = 8), in contrast to N-fertilization intensity (PERMANOVA, *R*^2^ = 0.07, *p* > 0.05, *n* = 8). **C** Enriched functional genes associated with bacteria in the rhizosphere of maize under extensive or intensive N-fertilization. Significant differences were tested for both N-fertilization intensities with edgeR and Benjamini–Hochberg correction (*p* < 0.05). Bold-marked genes indicate functional genes with 100% identity and alignment (DIAMOND) to *Bacillus atrophaeus* ABi03. Blue color indicates significant enrichment in the rhizosphere of control plants and orange color indicates enrichment in the rhizosphere of BMc inoculated plants

To track the inoculants in the metagenomic dataset, we mapped and annotated multiple sequences to the genomes of ABi03, RU47, and OMG16 (Additional file 2: Table S7). BMc inoculation significantly increased the relative abundance of reads assigned to *Bacillus atrophaeus* ABi03 (ANOVA, *F* = 96.0,* p* < 0.001, *n* = 4; Fig. 7A) and *Pseudomonas* sp. RU47 (ANOVA, *F* = 50.6, *p* < 0.001, *n* = 4; Fig. 7A), corroborating their successful establishment in the rhizosphere. In contrast, no such increase was observed for *Trichoderma harzianum* OMG16, likely due to the naturally high abundance of *Trichoderma* spp. in that field site (as also shown in Fig. 6D). To investigate the impact of BMc inoculation on microbial functions in the rhizosphere, metagenomic shotgun sequencing reads were mapped against a customized database targeting potential plant-beneficial microbial functions (Additional file 2: Table S8). BMc inoculation significantly influenced the functional composition of bacterial communities in the rhizosphere (PERMANOVA, *R*^2^ = 0.15, *p* = 0.0014, Fig. 7B) and led to a significant increase in the abundance of genes associated with chemotaxis, quorum sensing, the degradation of aromatic compounds, as well as synthesis of auxin, siderophores, lipopeptides (e.g., surfactin, iturin, athrofactin), and spermidine (*potA*) (edgeR, *p* < 0.05, *n* = 4. Figure 7C). Genes such as mycobactin synthetase (*mbtE*), involved in the synthesis of iron-chelating siderophores, were highly enriched due to BMc inoculation, particularly under extensive N-fertilization (Fig. 7C). In contrast, *lvH*, *dppA*, *rbsB*, *potD*, and 4,5-diphosphate decarboxylase (involved in type-3-secretion system, chemotaxis, spermidine production, and aromatic compound degradation, respectively) decreased with BMc inoculation (edgeR, *p* < 0.05, *n* = 4; Fig. 7C).

In order to link microbial taxa to their respective functions, we taxonomically annotated sequences assigned to differentially abundant functions. Approximately 66% of sequences assigned to specific functions met the criteria for reliable taxonomic classification. Eight genes associated with the production of lipopeptides were linked to *Bacillus* spp., as discerned by sequence similarity. The majority of these genes exhibited 100% identity and 100% alignment with ABi03 (reads from each gene annotated as ABi03: *fenD* 63%, *ituA* 75%, *ituB* 67%, *ituC* 61%, *srfAA* 79%, *srfAB* 54%, *srfAC* 74%, and *srfAD* 93%; Fig. 7C). Furthermore, five BMc-enriched genes associated with siderophore production (*dhbf* 64%, *entE* 66%, *entF* 66%, *mbtE* 63%, and *mbtF* 59% reads, Table 2) were mapped to bacterial taxa affiliated with the phylum Actinobacteriota, with the highest proportion of attributed sequences annotated as *Streptomyces* spp. (Additional file 1: Fig. S9). Notably, only a limited percentage of these genes was annotated to RU47 and ABi03 genomes, and these instances were exclusively restricted to the BMc samples (Table 2).

**Table 2:** Annotated siderophore genes in the shotgun sequencing data and their association with *Bacillus atrophaeus* ABi03 and *Pseudomonas* sp. RU47. In addition, the association of these genes to ABi03 and RU47 genomes was evaluated by DIAMOND (*e* = 10^−5^, identity = 100%)

Siderophore genes	Total counts	Number of counts annotated to genera	Number of genera annotated per gene	Read counts annotated as *Pseudomonas*	Read counts annotated as RU47 (100% identity)	Read counts annotated as *Bacillus*	Read counts annotated as ABi03 (100% identity)
2,3-Dihydroxybenzoate-AMP-ligase, *entE*	647	428	185	4	0	48	27
L-serine ligase, *entF*	3389	2242	59	167	0	30	26
Glycine ligase, *dhbF*	4889	3138	176	257	1	191	141
Mycobactin peptide synthetase, *mbtE*	1146	721	98	78	2	4	2
Mycobactin peptide synthetase, *mbtF*	579	344	56	5	0	8	6

### Improved plant micronutrient uptake, rhizosphere metabolite patterns, and microbiome modulation are linked to BMc inoculation

**Fig. 8:**
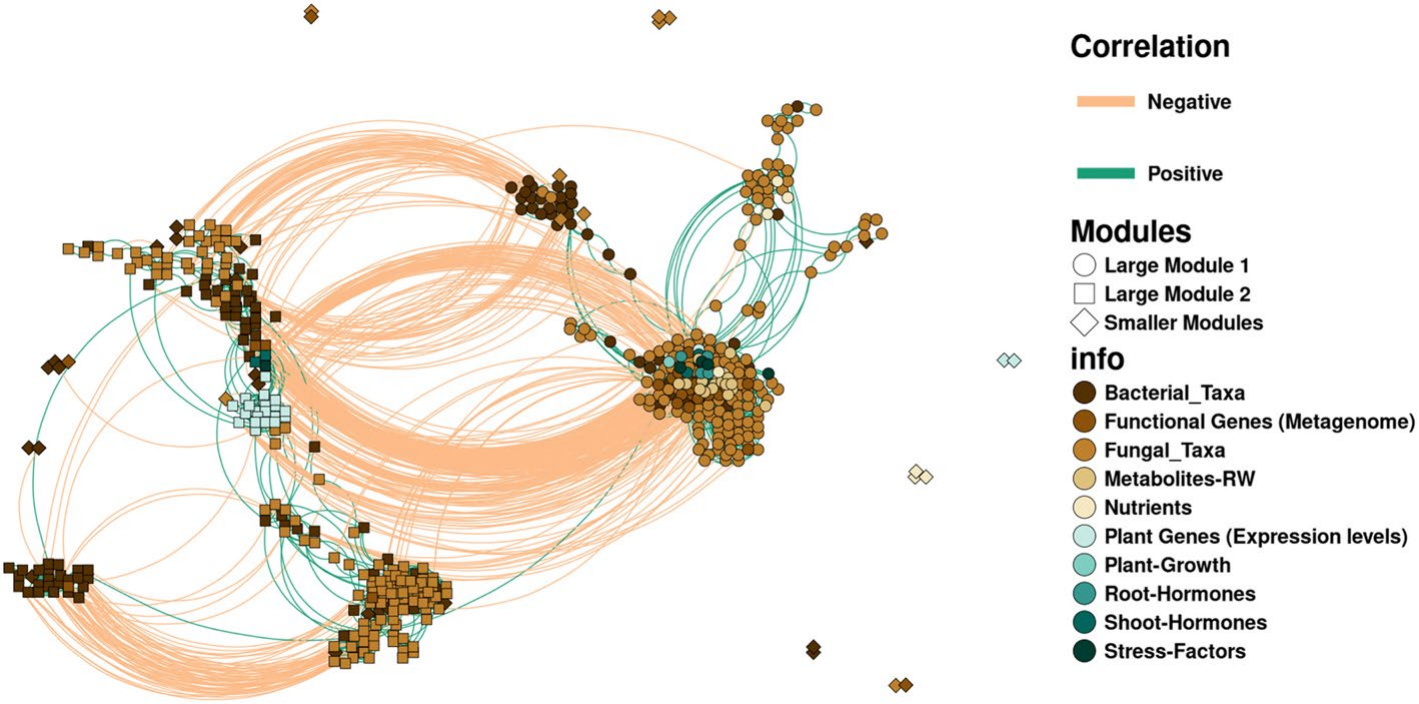
Integrated network analysis between different variables, which passed differential testing (*p* < 0.05) between BMc (beneficial microorganism consortium) vs. Ctrl (control) under extensive or intensive N-fertilization intensity. The network visualizes correlations with Pearson’s |*ρ*|> 0.8 and *p* < 0.05 following Benjamini-Hochberg. Clustering of variables was performed based on positive correlations. Therefore, groups that closely align in the network are positively correlated (due to the high degree of clustering, some positive correlations were not fully visualized by the colored lines). Modules were assigned based on positive correlations and the Louvain method (package “igraph”). We mainly divided the clusters into module 1 (positively correlated with plant growth) and module 2 (negatively correlated with plant growth). Smaller modules that did not cluster with plant growth are shown as well. Bacterial and fungal taxa were analyzed at the ASV level

To delve deeper into the interconnected shifts within the soil–plant system resulting from BMc application, we conducted an integrated network analysis, which included all plant physiochemical parameters, stress indicators, gene transcripts of leaves, rhizosphere metabolites, and microbial data. Only variables from each section that passed the above differential testing and showed exclusively highly significant correlations were included (Pearson |*ρ|*≥ 0.8, *p* < 0.05, Benjamini–Hochberg correction, *n* = 16). In addition, distance-based clustering was used to construct this network (Fig. 8, Additional file 2: Table S9). This analysis unveiled two primary clusters, module 1 and module 2, incorporating all variables positively or negatively correlated with maize growth, respectively (Fig. 8). Module 1 comprised ASV20, taxonomically linked to *Bacillus* ABi03 from the applied BMc (Additional file 1: Fig. S8 A), alongside various fungal ASVs taxonomically categorized as *Trichoderma* spp., although none was phylogenetically associated with *Trichoderma* OMG16 (Additional file 1: Fig. S8B). All of these were positively correlated with growth-promoting variables (Fig. 8, Additional file 2: Table S9). Interestingly, the concentrations of iron, zinc, and manganese, along with the functional genes (in the metagenome) encoding bacterial siderophore synthetases (mycobactin synthetase *MbtE*, Additional file 2: Table S9), displayed a positive correlation with maize growth and the expression of *NAS3*, a plant gene involved in iron/zinc translocation (Fig. 1C). All these positive correlations clustered in module 1, which indicated that BMc inoculation improved iron uptake by facilitating alterations in bacterial siderophore release and by stimulating root exudation of iron-mobilizing phenolics. This resulted in an improved iron-nutritional status, promoting ROS detoxification with protective effects under drought stress.

## Discussion

Intensified agricultural practices have revolutionized agricultural production enabling sustained high crop yields but often concurrently compromising soil health and plant resilience [1, 2, 39]. Therefore, implementation of sustainable farming practices is required, including environmentally sound methods for maintaining ecosystem functions. Recognizing the pivotal role played by rhizosphere microbiota in supporting plant performance, we hypothesized that the use of a beneficial microbial consortium (BMc) mitigates growth reductions associated with reduced (extensive) N-fertilization intensity. Contrarily to our expectations, BMc inoculation outperformed plants grown under intensive N-fertilization, demonstrating the potential of BMc inoculation for promoting sustainable agriculture through reduced reliance on mineral N-fertilizer inputs.

The observed increase in maize shoot biomass and concomitant improvement of plant fitness in BMc inoculated plants were likely attributable to the mitigated impact of drought induced by BMc inoculation. This association may be explained by the prolonged period of unusually low rainfall experienced during the early plant development stages of maize in our field experiment (Additional file 1: Fig. S1). Notably, no significant differences in shoot macronutrient concentrations were detected between control and BMc inoculated plants. However, the latter exhibited a significantly higher iron concentration compared to control plants, regardless of N-fertilization intensity. This observation is consistent with existing studies, which suggests a strong link between iron uptake and plant performance under drought stress conditions [40]. Indeed, recent research highlighted a notable reduction in the uptake of this micronutrient in *Poaceae* roots under drought conditions. For example, Xu et al. [40] employed root RNA-seq data to demonstrate that drought-stressed sorghum exhibited downregulated iron transporter genes and upregulated iron storage functionality. Similar observations were found in barley under water limiting conditions [41]. These findings support the notion that plants respond to drought by downregulating iron uptake genes and upregulating iron storage [42, 43]. This strategic metabolic shift is thought to address the drought-induced reduction in photosynthesis, as the photosynthetic machinery is a major consumer of iron in plants [44, 45]. Additionally, it might reflect the decreased iron availability in dry soils and the negative impact of drought on iron acquisition mediated by mugineic acid phytosiderophores [40]. Accordingly, no mugineic acids were detected the rhizosphere soil solutions collected in our study (Additional file 2: Table S4). Therefore, the prolonged drought during early maize growth could have limited iron uptake, consequently hindering plant development. Conversely, BMc inoculated maize plants exhibited a significant increase in the relative abundance of genes associated with microbial siderophore production (specifically, *dhbf*, *entE*, *entF*, *mbtE*, and *mbtF*). This finding supports the presumption of enhanced iron availability in BMc inoculated plants, potentially contributing to the mitigation of negative effects of severe drought conditions. In contrast to the stimulation of shoot growth, root development and root AMF colonization remained unaffected by BMc inoculation (Additional file 1: Fig. S2), suggesting that BMc inoculation promoted iron availability rather than spatial iron acquisition.

Interestingly, metagenomic analysis highlighted Actinobacteriota, particularly *Streptomyces* spp., as harboring genes for siderophore production. This finding aligns with recent research emphasizing the pivotal role of Actinobacteriota in mitigating drought stress in plants [40, 46–48], particularly by secreting various iron-chelating organic molecules that sequester and immobilize soluble iron (Fe^3+^) for plant uptake in iron-deficient soils [49, 50]. More specifically, the genus *Streptomyces*, renowned for its capacity to produce diverse hydroxamate siderophores via divergent biosynthetic gene clusters [51], has demonstrated an ability to attain a competitive growth advantage over other soil bacteria in environments characterized by limited iron availability [52, 53] and during periods of drought [54]. Network analysis further supported the potential benefits of Actinobacteriota, revealing a significant positive correlation between shoot dry mass and the presence of genes associated with Actinobacteriota-derived siderophores, underscoring their role in enhancing plant fitness. Control maize plants surprisingly also displayed a notable enrichment in Actinobacteriota, concomitant with a decrease in the relative abundance of taxa affiliated with the Bacteroidota and Proteobacteria phyla. This finding is consistent with observations commonly reported in moisture-limited soils, where an increase in the ratio of Gram-positive to Gram-negative bacteria is widely noted under drought conditions [55–57]. Specifically, an increase in the abundance of Actinobacteriota, coupled with a concurrent reduction of Proteobacteria and Bacteroidota in the plant rhizosphere under drought, has been frequently documented [58–62]. The fact that BMc inoculated plants exhibited an opposite pattern, with a decrease in Actinobacteriota and an enrichment of Gram-negative taxa, could further substantiate the drought mitigation effect promoted by the inoculated BMc. However, the decrease in Actinobacteriota abundance, along with the increase in the relative abundance of Actinobacteriota-related siderophore genes, could also indicate a direct interaction between the inoculated microbes and the resident rhizosphere microbiota. This interaction may selectively enrich Actinobacteriota taxa possessing siderophore synthesis capabilities while depleting antagonistic members within the same phylum. Unfortunately, the short-read metagenomic sequencing approach did not provide sufficient coverage to yield fully assembled genomes (Additional file 1: Fig. S10), preventing a comprehensive estimation of the genomic content for Actinobacteriota members positively associated with iron uptake. To address this limitation, further sequencing endeavors utilizing a combination of short- and long-read and Hi-C sequencing technologies could potentially enhance metagenome assemblies. This approach holds promise in facilitating a more accurate assessment of the genomic potential of the identified Actinobacteriota members, particularly those linked to iron uptake.

The maize rhizosphere microbiota exhibited significant responses to both N-fertilization intensities and BMc inoculation. Notably, these factors impacted fungal community assembly more than bacterial communities. BMc inoculation specifically led to an enrichment of fungal amplicon sequence variants (ASVs) with potential plant growth-promoting capabilities. This included *Talaromyces* spp., previously documented to enhance plant growth under controlled drought conditions [63–65]. Additionally, the rhizosphere of inoculated maize plants harbored a greater abundance of ASVs classified as *Trichoderma* spp., although not exhibiting a close phylogenetic relationship to the inoculated OMG16. Several *Trichoderma* strains are known for producing phytohormones and siderophores [66–70]. Therefore, it is plausible that these *Trichoderma* taxa contributed to the observed improvement in micronutrient uptake following BMc inoculation. It is worthwhile noting that the BMc strains used are commonly found in commercial biofertilizers and are known for their beneficial effects on plants. These strains possess documented beneficial traits, including modulation of plant hormones, antagonism against pathogens, and promotion of plant nutrition [13]. Among them, ABi03 displayed the highest density and relative abundance in the maize rhizosphere, indicating superior rhizosphere competence compared to the other strains. Integrated network analysis identified ABi03 as a key driver of improved nutrient uptake, leading to increased shoot dry mass and concomitant alterations in plant gene transcription, hormonal levels, stress factors, and root exudate composition. However, the possibility that RU47 and OMG16 persisted in sufficient densities during initial inoculation stages and subsequently declined over time cannot be excluded.

The establishment of rhizosphere microorganisms is highly influenced by root exudates [71–73]. To comprehensively assess the impact of BMc inoculation on the composition of root exudates and rhizosphere soil solution, root windows were installed. This methodology facilitated a nuanced understanding of the intricate interplay between BMc and the specific root exudates that shape the rhizosphere microbial community. Plants inoculated with BMc exhibited higher levels of organic acids, sugars, and amino acids in their rhizosphere solution, indicating enhanced physiological activity of roots and rhizosphere microbes. The increase in trehalose within the rhizosphere metabolite profile of BMc inoculated plants could be associated with BMc-induced adaptations of maize plants to drought conditions. Indeed, trehalose was extensively documented for its role in improving adaptation to water deficit in various crops, including maize [74–76]. Additionally, BMc promoted rhizosphere accumulation of several secondary metabolites known to influence rhizosphere microbiota, including phenolic acids, flavonoids, and benzoxazinoids. The latter, a fascinating class of secondary metabolites mainly known from the *Poaceae*, exhibit a multifaceted role in plant physiology. Benzoxazinoids are renowned for their allelopathic effects, herbivore deterrence, and antimicrobial activity, significantly contributing to plant resilience [77–79]. In addition to their role as defense mechanism activators, recent research has revealed that benzoxazinoids exhibit siderophore-like properties, demonstrating a high affinity to chelate metals, particularly iron [77, 80, 81]. This property also applies to phenolic acids and flavonoids, which accumulated in the rhizosphere in response to BMc inoculation [82, 83]. This siderophore capability has implications for nutrient acquisition, potentially influencing plant-microorganism interactions and nutrient cycling within the rhizosphere. The detected levels of these metabolites (10–15 µg cm^−1^ root length, Fig. 4) translate to rhizosphere concentrations in the millimolar range, which are sufficiently high to mediate the mobilization of mineral nutrients [84]. Furthermore, benzoxazinoids can act as chemo-attractants for beneficial microorganisms, including *Pseudomonas* and *Bacillus*. The tolerance of these bacteria to benzoxazinoids may explain the observed advantage of ABi03 in achieving high densities in the maize rhizosphere. Bacterial tolerance to benzoxazinoids, particularly MBOA, likely influences the benzoxazinoid-dependent community structure of the maize rhizosphere microbiome [85–87]. Moreover, recent studies have reported increased accumulation of jasmonic acid (JA) in oilseed rape inoculated with *Bacillus* spp. and *Trichoderma* OMG16 [32], similar to the increased JA levels observed in the root tissue of BMc inoculated maize plants in this study (Additional file 1: Fig. S3). Interestingly, JA triggers the biosynthesis of benzoxazinoids [88]. Therefore, the BMc may have induced the production and release of benzoxazinoids by stimulating JA accumulation. The intricate interplay between benzoxazinoids and siderophore dynamics underscores the multifaceted and integral role these compounds play within the broader ecological and physiological context. These observations also suggest a potential direct effect of BMc on maize metabolic patterns, leading to a shift in root exudate production and, consequently, shaping bacterial and fungal community compositions in the rhizosphere.

Our findings suggest that BMc inoculation potentially exerted systemic mitigation effects on the prolonged and severe drought stress experienced by the investigated maize plants during their early growth stages. Supporting this hypothesis, a significant decrease in the expression of various maize genes associated with metabolic stress was observed in the leaf tissue of BMc inoculated plants. Furthermore, a reduction in shoot stress indicators, including hydrogen peroxide accumulation, ABA, and JA levels, was accompanied by elevated gene expression and activities of key iron-dependent enzymes involved in hydrogen peroxide detoxification, such as ascorbate peroxidase and superoxide dismutase. Additionally, there was an increase in glycine betaine, an essential organic osmolyte that enhances plant responses to water stress. Apart from alleviating metabolic stress, BMc inoculation also led to increased concentrations of plant growth-promoting phytohormones (IAA and cytokinins). While the precise mechanisms underlying the observed alterations in plant hormone and metabolic stress profiles remain elusive, potentially involving enhanced micronutrient uptake or direct cross-signaling with the applied BMc, our results demonstrate that BMc inoculation promoted plant health and mitigated the negative impacts of drought conditions.

## Conclusions

Our study demonstrates beneficial effects of BMc inoculation in enhancing maize growth under both conventional and reduced N-fertilization intensities under field conditions. We elucidate the intricate interplay between BMc strains, iron metabolism, drought conditions, the rhizosphere microbiome, and root exudate profiles. This highlights the efficacy of a multidisciplinary approach in unraveling these complex relationships. Furthermore, we demonstrate the potential application of BMc alongside reduced N-fertilization intensity to promote soil health and sustainable agriculture without compromising crop productivity. BMc inoculation not only modulates the rhizosphere microbiome but also concurrently alters root exudation patterns, thereby enhancing iron uptake and overall plant health. Our observations reveal that BMc inoculation significantly influences the assembly of maize rhizosphere microbiota while simultaneously increasing the abundance of genes related with microbial siderophores and the root exudation of benzoxazinoids, which are potential iron chelators. These findings hold particular relevance for alleviating iron deficiency, a common consequence of drought conditions in the rhizosphere. This indicates the potential of BMc to mitigate drought effects in long-term applications and foster sustainable agriculture in regions suffering from extended drought periods.

## Methods

### Experimental design at long-term field trial

An experiment was conducted within an ongoing long-term field trial (LTE Westerfeld, 10.20387/bonares-9ne9-a828) in Bernburg, Germany (51° 82′ N, 11° 70′ E) to assess the impact of a beneficial microorganism consortium (BMc: *Pseudomonas* sp. RU47 (DSM 117411), *Bacillus atrophaeus* ABi03 (DSM 32285), and *Trichoderma harzianum* OMG16 (DSM 32722)) on maize performance (*Zea mays* cv. Benedictio, KWS Saat SE & Co. KGaA) during the 2020 growing season. The LTE, with the soil type loess chernozem over limestone (8% sand, 70% silt, 22% clay; pH 7.0–7.4), was described in detail by Sommermann et al. [89]. The respective field plot (0.6 ha), managed with cultivator tillage (12–15 cm depth), was further subdivided into strips with reduced nitrogen (N) fertilization intensity (40 kg ha^−1^ N) without fungicide use (extensive; Ext) compared to conventional N-fertilization intensity (100 kg ha^−1^ N, intensive; Int) with recommended pesticide (including fungicide) application. The strips were divided into four blocks in which the treatments were arranged. Controls (Ctrl) and maize BMc root inoculated plants were arranged at a distance of 2 m, within the respective blocks forming subplots (size 2.25 m^2^). Therefore, each treatment (Ctrl-Ext, BMc-Ext, Ctrl-Int, and BMc-Int) included four subplot-replicates with 33 maize plants each, with an intra-row distance of 14 cm between plants and inter-row distance of 60 cm.

### Inoculum preparation and BMc application

An endospore suspension of a rifampicin-resistant *B. atrophaeus* ABi03 selectant was provided by ABiTEP GmbH (Berlin, Germany). The inoculum of *T. harzianum* OMG16 (strain collection of Anhalt University of Applied Sciences, Bernburg, Germany) was prepared as described by Hafiz et al. [32]. The inoculum of the rifampicin-resistant *Pseudomonas* sp. RU47 selectant (DSM 117411) was grown in nutrient broth (Sifin diagnostics GmbH, Germany) supplemented with rifampicin (75 µg ml^−1^; Th. Geyer GmbH & Co. KG, Germany) on a rotary shaker (200 rpm) at 28 °C for 24 h to obtain fresh viable cells. RU47, ABi03, and OMG16 were mixed in tap water to a final concentration of 10^8^ CFU ml^−1^ per strain immediately before drenching the roots. Each maize plant was drenched at 2 and 5 weeks after emergence (EC 12 and EC 14) with 50 ml BMc-suspension. The control plants were drenched with 50 ml tap water at the respective inoculation times.

### Installation of root windows

To study effects of the experimental variables on root exudation and rhizosphere metabolite profiles, root windows were installed in each control and BMc-treated subplots/replicates prior to the second drenching, according to Neumann et al. [90]. Specifically, soil profiles measuring 50 × 50 cm were excavated adjacent to maize plants at EC 14–16 using steel plates. After removing the plates, the profiles were covered with plexiglass sheets stabilized with wooden timbers inserted into the soil. Subsequently, the root windows were thermally insulated with two layers of Styrofoam® panels and finally covered with black plastic foil and soil to prevent light and rain exposure (Additional file 1: Fig. S5).

### Sampling and sample processing

For plant molecular (23 stress-related genes and 16 genes related to nutrient uptake and metabolism) and physiological analyses (stress indicator), a sample (3 × 2 cm from the middle portion of the leaf lamina) from the youngest fully developed leaf was collected at the flowering stage (EC 53–63, three maize plants per treatment replicate). The leaf material from each treatment was pooled and immediately immersed in a total of 10 ml of RNAlater (Thermo Fisher Scientific, Darmstadt, Germany). The samples were incubated at 4 °C overnight and then stored at − 80 °C. After grinding the leaf material in liquid nitrogen, total RNA was extracted from 100 mg of the homogenized sample using the RNeasy Plant Mini Kit (Qiagen GmbH, Hilden, Germany) and quantified spectrophotometrically. Single-stranded cDNA synthesis from 2 µg of total RNA was performed with the High Capacity cDNA Reverse Transcription Kit with RNase Inhibitor (Applied Biosystems, Foster City, CA, USA), and qPCR was performed as previously described [91]. The selected targeted genes and corresponding primer pairs are listed in Additional file 2: Table S3. These genes were selected based on their association with biotic and abiotic stress responses and nutrient acquisition. In addition, several genes involved in N-metabolism and Fe-transport were analyzed. An aliquot of the leaf material preserved in RNAlater was used for the analysis of plant hormones and stress-associated metabolites and enzymes (see below).

Following leaf sampling, these maize plants were excavated, the shoots were separated from the roots, and soil samples were collected. Shoots, roots, and soil samples were stored immediately at 4 °C. Maize shoots were dried at 60 °C for 48 h, then shoot dry mass (SDM) was measured. Shoot and soil nutrient content was analyzed as described by Behr et al. [13].

#### Sampling of maize rhizosphere for microbial analysis

Per subplot/replicate, root material from three plants was pooled. First, the roots with adhering soil were shaken vigorously to remove loosely adhering soil, and the roots were briefly washed with sterile tap water. The root systems with strongly adhering soil of all plants per replicate were cut and homogenously mixed. From this mixed root sample, 5 g was used to recover rhizosphere microbial cells by three 1-min blending steps in a Stomacher 400 Circulator (Seward Ltd, Worthing, UK) each with 15 ml of 0.3% NaCl, as described previously [92]. One milliliter of the rhizosphere suspension was used to determine the rhizosphere competence of the individual strains of BMc via serial dilution plating counts, following the method described by Behr et al. [13]. Serial dilutions were also plated for the control plants. The remaining rhizosphere suspension was centrifuged to harvest the microbial rhizosphere fraction [90]. Total community DNA was extracted from total rhizosphere pellets using the FastPrep-24 bead-beating system and FastDNA Spin Kit for Soil (MP Biomedicals, Santa Ana, CA, USA) following the manufacturer’s recommendations. DNA samples were further purified with the GeneClean Spin Kit (MP Biomedicals, Santa Ana, CA, USA).

#### Analysis of root traits and mycorrhization

The WinRHIZO root analysis system (Regent Instruments, Quebec, Canada) was used to measure total root length (TRL) of the collected roots by optical scanning. Root hair length was determined after magnification of high-resolution digital photographs taken from roots growing along the observation windows (Zeiss Axiovision software, Oberkochen, Germany). For analysis of mycorrhization, a modified staining method following Vierheilig et al. (1998) was used. Collected roots were washed and cut into segments of 1–2 cm and incubated at 90 °C for 45 min in 10% (w/v) KOH. Bleached roots were acidified with 1% HCl and then stained in a 5% ink-vinegar solution (5% v/v ink in 5% v/v acetic acid) at 90 °C for 10 min. Destaining was performed with acidified tap water. The grid–line intersection method of Giovannetti and Mosse [93] was employed to determine the rate of mycorrhization.

#### Sampling rhizosphere solution from root window plots for metabolite profiling

The collection of rhizosphere solutions from the root windows was conducted on the same day as the collection of soil and plant materials described previously, by following the procedure outlined by Neumann et al. [94] (Additional file 1: Fig. S5). After root window opening, moist filter discs (5 mm diameter) were placed onto the surface of first order lateral roots in subapical root zones (1–2 cm behind the root tip), mature root zones (8–10 cm behind the root tip), crown roots, and soil areas without visible roots. Two filters were applied for each root zone in five replicates, covering root segments of at least 1 cm per replicate. After a sampling period of 4 h, the filters were removed and pooled in 2 ml of 80% v/v methanol solution. The sorption filters were precipitated by centrifugation for extraction, and the supernatants were filtered (Chromafil R O-20/15MS) and stored at − 80 °C.

### Amplicon sequencing of bacterial and fungal rhizosphere communities

For prokaryotic community analyses, DNA from the maize rhizosphere was used for amplification of 16S rRNA gene fragments using the 16S primers Uni341 F (5′-CCTAYGGGRBGCASCAG-3′) and Uni806R (5′-GGACTACNNGGGTATCTAAT-3′) targeting bacteria and archaea [95]. Library construction and sequencing was carried out by Novogene (Cambridge, UK) on NovaSeq 6000 PE250 (Illumina, Cambridge, UK). Primers and adapters were removed from sequences using cutadapt [96] (v3.7). Paired-end reads were processed using the DADA2 pipeline [97] (v1.26.0) using R [98] (v.4.2.2). The obtained amplicon sequence variants (ASVs) were taxonomically classified to the lowest possible taxonomic level by using a Naive Bayesian Classifier [99], trained on the SILVA SSU Reference Taxonomy database [100] (v138.1). Sequences originating from chloroplasts or mitochondria and sequences with less than five reads were removed. Following these steps, no sequence classified as archaea was present in the final dataset. A total of 1.35 × 10^6^ out of 4.31 × 10^6^ reads (31.8 ± 5.2%) were retained after processing, generating 6595 bacterial ASVs in total with an average of 42,114 reads per sample.

In parallel, we investigated the fungal community in the rhizosphere by performing sequencing of the Internal Transcribed Spacer (ITS2) region. Briefly, three PCRs per sample were carried out at three annealing temperatures (56 °C ± 2 °C) with 24 cycles in 20 µl volume using Q5® High-Fidelity 2 × Master Mix (New England Biolabs). Each PCR included 10 ng TC-DNA and bovine serum albumin (final concentration 0.5 mg ml^−1^), and PCRs were performed using the standard 8-nucleotide Illumina barcoded ITS86 F/ITS4 primer pair for PCR amplification [89]. High-throughput amplicon sequencing was carried out on the Illumina® MiSeq® platform in paired-end mode (2 × 300 bp) as previously described [101]. ASV generation and taxonomic assignment based on database-dependent strategy [102] using the GALAXY platform and UNITE v9.0 database [103, 104] was performed as described in Behr et al. [105]. A total of approximately 3.43 × 10^6^ out of 3.82 × 10^6^ reads (89.8% ± 1.5%) were retained, and after singleton removal the total number of fungal ITS2-ASVs was 1965 (average of 107,251 reads per sample). Rarefaction curves were generated for estimating the read coverage of each sample (Additional file 1: Fig. S11).

### Library preparation and shotgun sequencing for metagenome analysis

Shotgun metagenomic libraries for functional analysis of rhizosphere DNA samples were prepared using NEBNext Ultra II FS DNA Library Prep Kit® (New England Biolabs, Frankfurt, Germany) according to the manufacturer’s protocol with modifications as described previously [105]. The libraries were diluted to 1 nM and sequenced on a NextSeq550 sequencer (Illumina, San Diego, CA, USA) using the NextSeq 500/550 High Output Kit v2.5 (300 cycles) after equimolar pooling. The shotgun metagenomic sequencing generated 186,817,794 forward and 186,817,794 reverse high-quality raw reads with a sequencing depth of 16 × 10^6^ reads per sample. Trimming and merging resulted in a total of 170,500,215 merged sequences (library size range 7,168,494–18,104,610) representing the majority of reads (91.3%). Nonpareil output showed coverage between 5.4 and 13.9%, which indicated a sufficient coverage for read-based analyses (Additional file 1: Fig. S10). Taxonomic and functional annotation were conducted following the methodology outlined in our prior study [105]. For detection of the applied BM, sequences taxonomically assigned to *Trichoderma harzianum*, *Bacillus atrophaeus*, and *Pseudomonas* sp. RU47 were extracted. In order to confirm whether these sequences actually represent the applied BM, sequences were compared with the respective BM genomes using NCBI BLAST (blastn [106]) with 100% sequence identity and 100% sequence alignment. Additionally, sequences of differentially abundant genes were compared with the genomes of the BM to estimate their potential contribution to the respective function, such as iturin, fengycin, surfactin, and genes associated with siderophore production.

### Determination of plant hormones and stress metabolites

The plant hormones (indole acetic acid, gibberellic acid, cytokinin, abscisic acid, jasmonic acid, and salicylic acid) were analyzed from shock-frozen maize shoot and root material. UHPLC-MS analysis of phytohormones was carried out on a Velos LTQ System (Thermo Fisher Scientific, Waltham, MA, USA) utilizing a Synergi Polar column, 4μ, 150 × 3.0 mm (Phenomenex, Torrance, CA, USA) as described by Moradtalab et al. [107]. Part of the same material was also used for the determination of selected stress metabolites via spectrophotometry (Spectrophotometer, Hitachi, Tokyo, Japan). After methanol extraction (80% v/v methanol) at 750 nm, total phenolics were determined using the Folin method [108]. Proline analysis was conducted at 520 nm after acetic acid and acid ninhydrin derivatization [109]. Glycine betaine determination was performed according to the method of Grieve and Grattan [110] with modifications described by Valadez-Bustos et al. [111]. The 1,1-diphenyl-2-picrylhydrazyl radical (DPPH) method was used to evaluate the free radical scavenging activity of antioxidants in the plant tissue [107]. Hydrogen peroxide levels were determined at 390 nm as described previously [109]. Ascorbate peroxidase (APX, EC 1.11.1.11) activity was recorded according to the method described by Boominathan and Doran [112]. Extraction and determination of superoxide dismutase (SOD, EC 1.15.1.1) was optimized for shoot tissue of maize according to the method of Moradtalab et al. [109].

### High-performance liquid chromatography profiles of rhizosphere soil solutions

HPLC profiling of organic acids, sugars, and amino acids in the rhizosphere soil solutions in 80% methanol extracts of the root windows sorption filters was conducted as described by Windisch et al. [91]. For phenolic compounds and benzoxazinoids, identification was performed with positive/negative switching LC–MS on a QExactive Plus Electrospray Mass Spectrometer (Thermo Fisher Scientific) coupled to an Agilent 1290 Ultra Performance Liquid Chromatography System. Details of the HPLC conditions are presented in Additional file 2: Table S10. A list of investigated phenolics is reported in Additional file 2: Table S4.

### Data analysis and statistics

Statistical analysis of SDM and nutrient data was performed using R [98] (v.4.2.2). Main and interaction effects between fertilization intensity and BMc inoculation were analyzed by two-way ANOVA for SDM, shoot/soil nutrients, and BMc rhizosphere competence. Homoscedasticity and normal distribution of residuals was inspected visually using the performance package [113] (v.0.10.2). Pairwise comparison was conducted using the Tukey’s HSD test of the agricolae package [114] (v.1.3.5). Data were visualized using the ggplot2 [115] (v.3.4.1) and ggpattern packages [116] (v.1.0.1). For gene expression data, we performed *t*-test (Ctrl vs. BMc).

For handling the sequencing data, we mainly used the “tidyverse” set of packages [117] (v.1.3.1⁠) and vegan [118] (v.2.6.1⁠). Samples were analyzed in relative abundance (% of reads) for sequencing data. Bray–Curtis distance was used for estimating the β-diversity with log_10_ transformed datasets and pseudocount addition (only amplicon sequencing data). PERMANOVA tests were applied to evaluate how the N-fertilization intensity or BMc inoculation affected bacterial and fungal β-diversity by using the vegan package [118]. Multidimensional scaling (MDS) plots were used to visualize beta-diversity results.

For investigating the effect of BMc inoculation on different bacterial and fungal taxa, we performed logistic regression, and *p* values were corrected with Benjamini-Hochberg. The differential abundance testing focused on the rhizosphere and was performed separately for intensive and extensive fertilization. The tests were applied for several taxonomic groups, starting from the phylum and reaching up to the ASV level. For metagenomic shotgun sequencing data, we performed differential abundance testing via edgeR [119] (v2.1.2⁠) with maximum likelihood test and *p* values corrected with Benjamini–Hochberg method. The algorithm edgeR was selected for metagenomics data due to its high sensitivity for metagenomics datasets [120]. For each differential abundance test, the minimum cut-off was 75%. Comparisons with *p* values below 0.05 were considered statistically significant (*α* = 0.05). To estimate whether an ASV corresponds to the 16S rRNA gene or ITS from our BMc strains, we performed phylogenetic association of responder ASVs to BMc inoculation with the 16S rRNA gene or ITS2 DNA sequence from our BMc strains. In addition, we also downloaded 16S rRNA gene and ITS2 sequences from the NCBI type strains for comparison. We performed multiple sequence alignments using the package DECIPHER [121] (v2.26.0).

An integrated network analysis was performed with the variables (e.g., SDM, plant gene expression, bacterial/fungal ASVs) that passed the differential testing. The aim of this network was to visualize the intercorrelation of different variables affected by BMc inoculation in a PCA-like approach. We selected this approach over PCA to ensure a more optimal visualization of the data. Thus, to create the network, we performed multiple Pearson correlation (*p* values were corrected with the Benjamini–Hochberg method), which is inferred through covariance, a necessary part for inferring principal components in PCA. We included only correlation with |*ρ|*> 0.8 and *p* < 0.05. Distance was based on Pearson’s rho coefficient. Clustering was carried out by using the positive correlation coefficients and the Louvain method to divide the network into different modules (package “igraph,” v1.5.1 [122]). The network was visualized with the packages “igraph” and “ggraph” [123] (v2.1).

## Supplementary Information

Additional file 1: Fig. S1 Rainfall during 2020 growing season and average rainfall from 1982 to 2010. Fig. S2 Root traits measured in roots of maize grown under different fertilization intensities. Fig. S3 Plant hormone concentrations measured in roots of maize grown under different fertilization intensities. Fig. S4 Principal component analysis of different plant physiochemical characteristics, stress-indicator phytohormones, shoot nutrient concentrations, and gene transcripts in leaves. Fig. S5 Root windows installed in the field. Fig. S6 Heatmap of the concentrations of low-molecular weight rhizosphere metabolites across three root zones of maize grown under different fertilization intensities and inoculation treatments. Fig. S7 Alpha-diversity plots of bacterial and fungal communities. Fig. S8 Clustering based on Hamming distance of *Bacillus* and *Trichoderma* ASVs. Fig. S9 The relative abundance of differential abundant siderophore-related genes and their association with bacterial genera. Fig. S10 Nonpareil curve for coverage of diversity for the metagenomic reads. Fig. S11 Rarefaction curves for diversity coverage through amplicon sequencing of 16S rRNA gene or ITS2 fragments.

Additional file 2: Table S1 Two-way ANOVA test of the main effect and interactions between different long-term N-fertilization practices and application of beneficial microorganism consortium on the nutrient content of maize shoots. Table S2 Effect of BMc inoculation on the chemical properties of soils with different long-term N-fertilization intensities. Table S3 List of plant genes used for gene expression analyses, along with their annotation and PCR primers. Table S4 Detected phenolic compounds in the rhizosphere of maize inoculated with a consortium of beneficial microorganisms. Table S5 Bacterial ASVs that passed the differential abundance testingfor extensive or intensive fertilization. Table S6 Fungal ASVs that passed the differential abundance testingfor extensive or intensive fertilization. Table S7 Detailed information on metagenomics sequences annotated to ABi03, RU47, and OMG16 genomes. Table S8 List of potential plant-beneficial functions of rhizosphere microorganisms in a customized database, established including protein sequences downloaded from online Kyoto Encyclopedia of Genes and GenomesOrthology database. Table S9 Variables of the two main modulesof the integrated network. Table S10 HPLC conditions for determination of phenolic compounds in rhizosphere soil solutions of maize.

## Data Availability

Sequencing data (bacterial amplicons and metagenome shotgun) were deposited at the Sequence Read Archive (https://www.ncbi.nlm.nih.gov/sra) under the BioProject accession PRJNA1045550 [124], while fungal ITS2 amplicon sequences [125] can be found under PRJEB74508. All scripts including detailed parameters and data to reproduce the figures for this study have been deposited in GitHub [126] and Zenodo [127]: GitHub: https://github.com/JonKampouris/BMc_inoculants_on_promoting_plant_growth Zenodo: 10.5281/zenodo.15334727 The source code provided in GitHub and Zenodo is released under an open source license compliant with OSI (http://opensource.org/licenses).
