# Supplementary material for: Microbial inoculants modulate the rhizosphere microbiome, alleviate plant stress responses, and enhance maize growth at field scale
Source: Genome Biol. 2025 Jun 1;26:148. doi: 10.1186/s13059-025-03621-7 (PMC12128319; doi:10.1186/s13059-025-03621-7)
Supplement: Supplementary file 1 — Additional file 1: Fig. S1 Rainfall during 2020 growing season and average rainfall from 1982 to 2010. Fig. S2 Root traits measured in roots of maize grown under different fertilization intensities. Fig. S3 Plant hormone concentrations measured in roots of maize grown under different fertilization intensities. Fig. S4 Principal component analysis of different plant physiochemical characteristics, stress-indicator phytohormones, shoot nutrient concentrations, and gene transcripts in leaves. Fig. S5 Root windows installed in the field. Fig. S6 Heatmap of the concentrations of low-molecular weight rhizosphere metabolites across three root zones of maize grown under different fertilization intensities and inoculation treatments. Fig. S7 Alpha-diversity plots of bacterial and fungal communities. Fig. S8 Clustering based on Hamming distance of Bacillus and Trichoderma ASVs. Fig. S9 The relative abundance of differential abundant siderophore-related genes and their association with bacterial genera. Fig. S10 Nonpareil curve for coverage of diversity for the metagenomic reads. Fig. S11 Rarefaction curves for diversity coverage through amplicon sequencing of 16S rRNA gene or ITS2 fragments. [file 13059_2025_3621_MOESM1_ESM.docx]

**Supplementary Material**

**
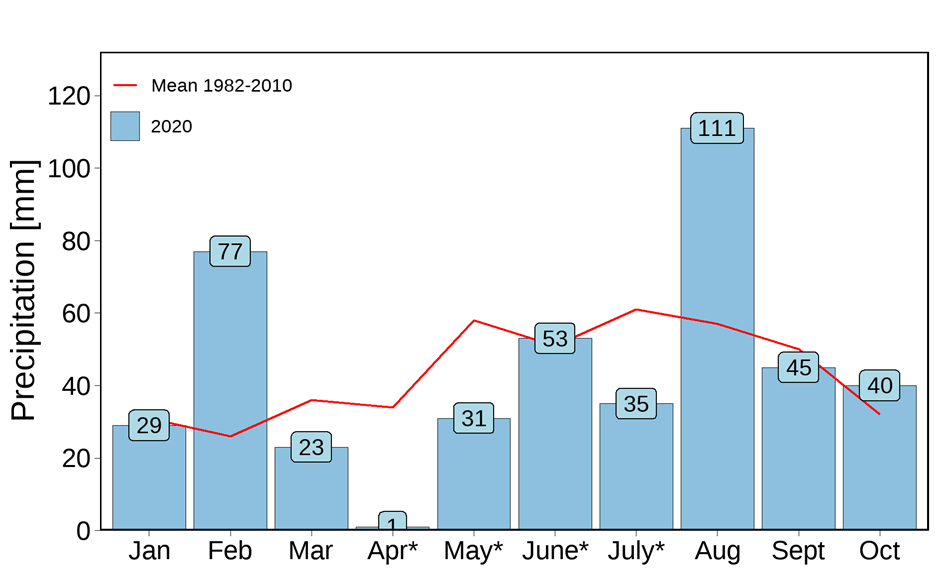
**

**Fig. S1.** Rainfall during 2020 growing season and average rainfall from 1982 to 2010. Asterisks (*) indicate the months were the BMc inoculation experiment was performed.


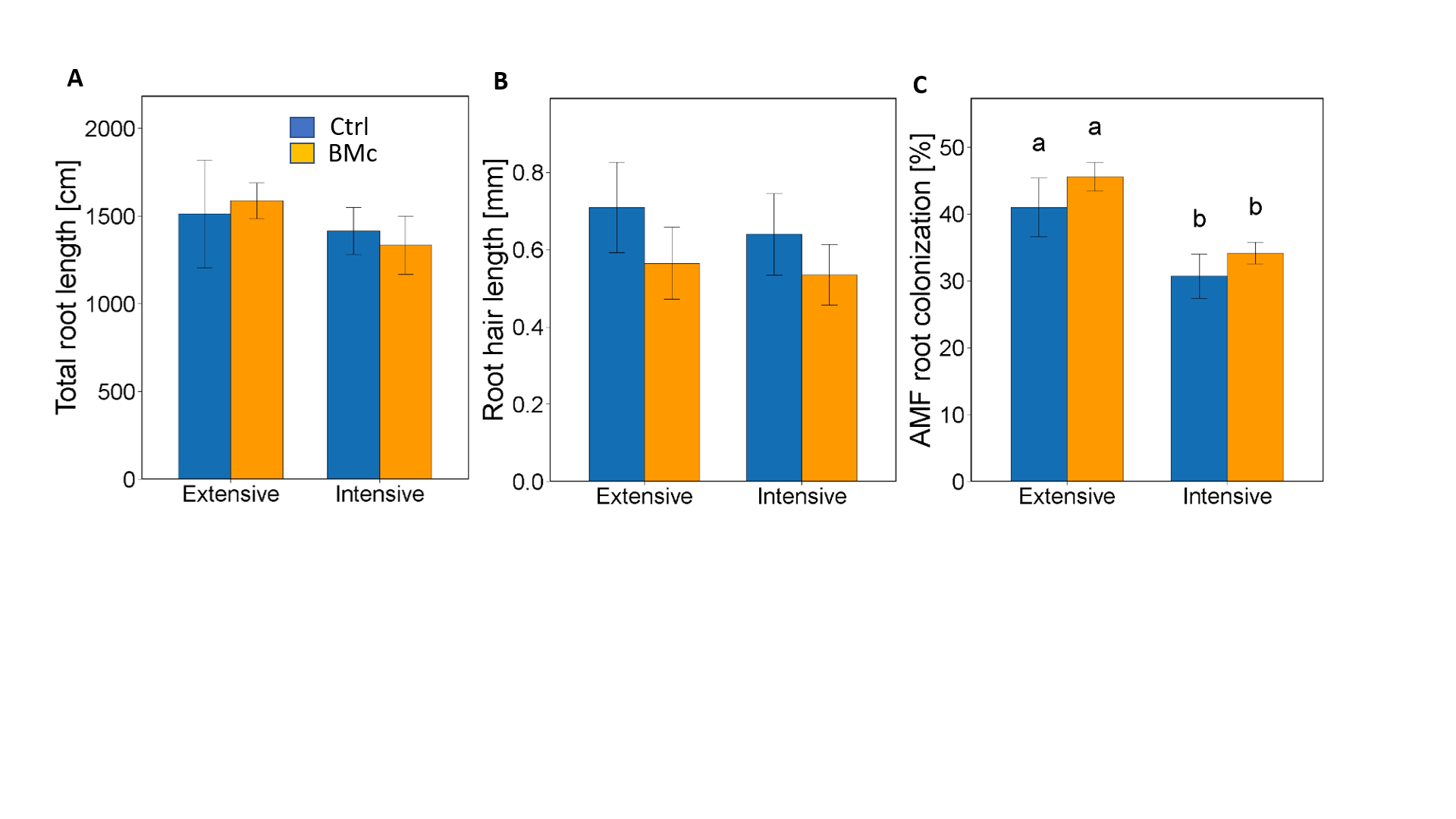


**Fig. S2.** Root traits measured in roots of maize (*Zea mays* cv. Benedictio) grown under different fertilization intensities (extensive vs intensive and inoculation vs control). Data represent mean values of four biological replicates ± standard deviation. Different letters indicate significant differences at *p*<0.05 according to Tukey’s HSD post hoc test. BMc: Beneficial microorganisms consortium. Ctrl: Control. AMF: arbuscular mycorrhizal fungi.


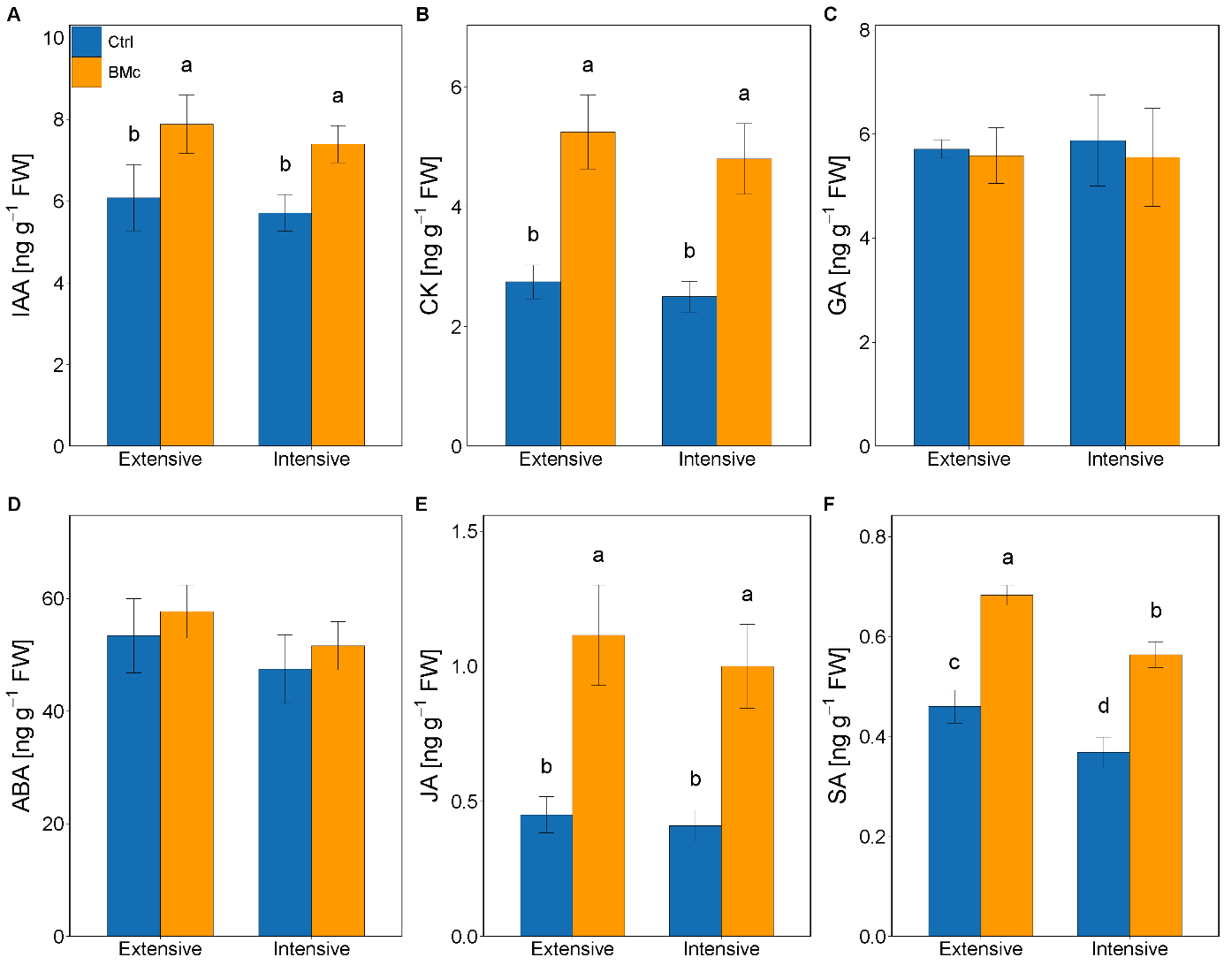


**Fig. S3.** Plant hormone concentrations measured in roots of maize (*Zea mays* cv. Benedictio) grown under different fertilization intensities (extensive vs intensive and inoculation vs control). A, Auxin, B, Cytokinin. C, Gibberellic acid. D, Abscisic acid. E, Jasmonic acid. F, Salicylic acid. Data represent mean values of four biological replicates ± standard deviation. Different letters indicate significant differences at *p*<0.05 according to Tukey’s HSD post hoc test. BMc: Beneficial microorganisms consortium. Ctrl: Control. FW: fresh weight.


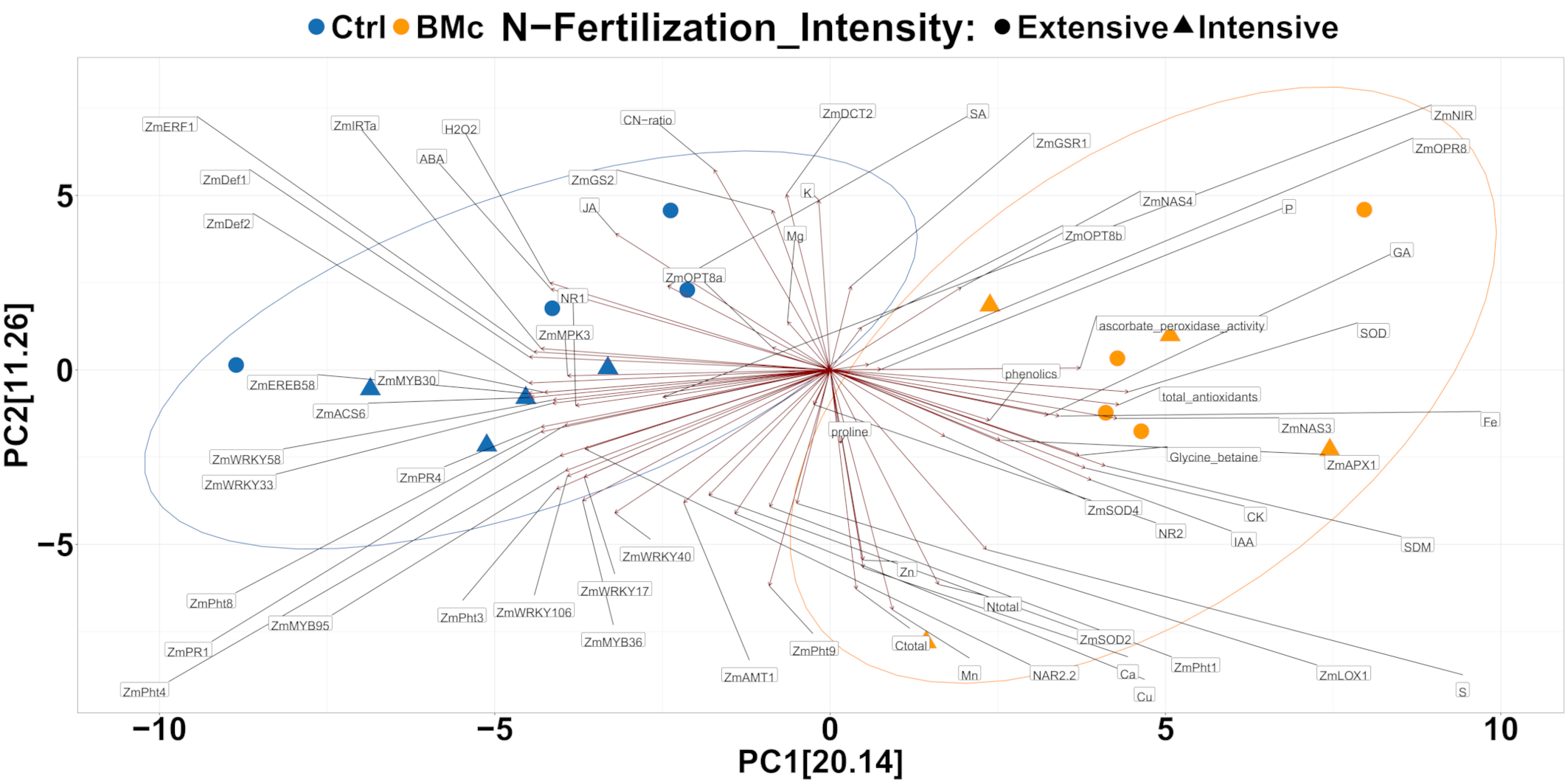


**Fig. S4.** Principal component analysis of different plant physiochemical characteristics, stress-indicators phytohormones, shoot nutrient concentrations, and gene transcripts in leaves. PERMANOVA test BMc Inoculation: R^2^ = 84.2%, *p* = 0.0001; N-Fertilization Intensity: R^2^ = 0.8%, *p* = 0.45; Interaction: 0.2%, *p =* 0.75 (n = 16). BMc: Beneficial microorganisms consortium. Ctrl: Control.


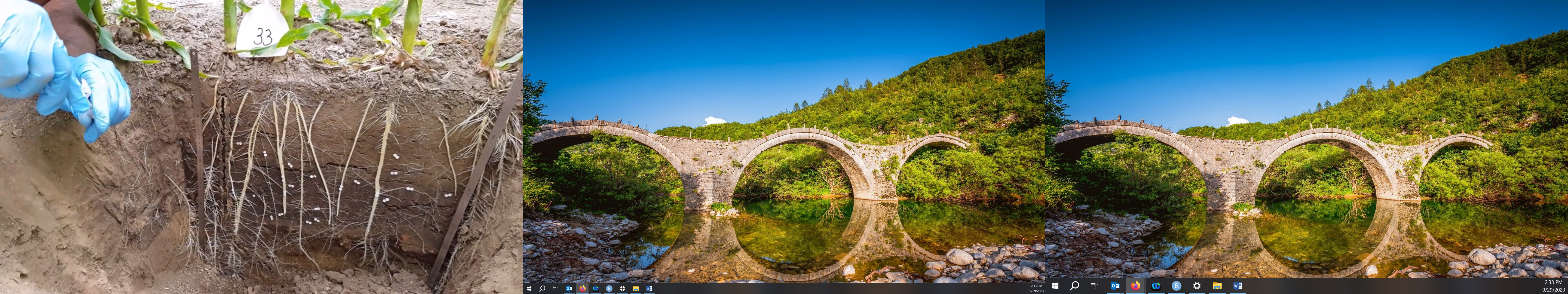


**Fig. S5**. Root windows installed in the field. The installation can be watched under the following link: http://dicontrol.igzev.de/wp-content/uploads/2021/01/Root-windows_Moradtalab_Neumann_2020.mp4.


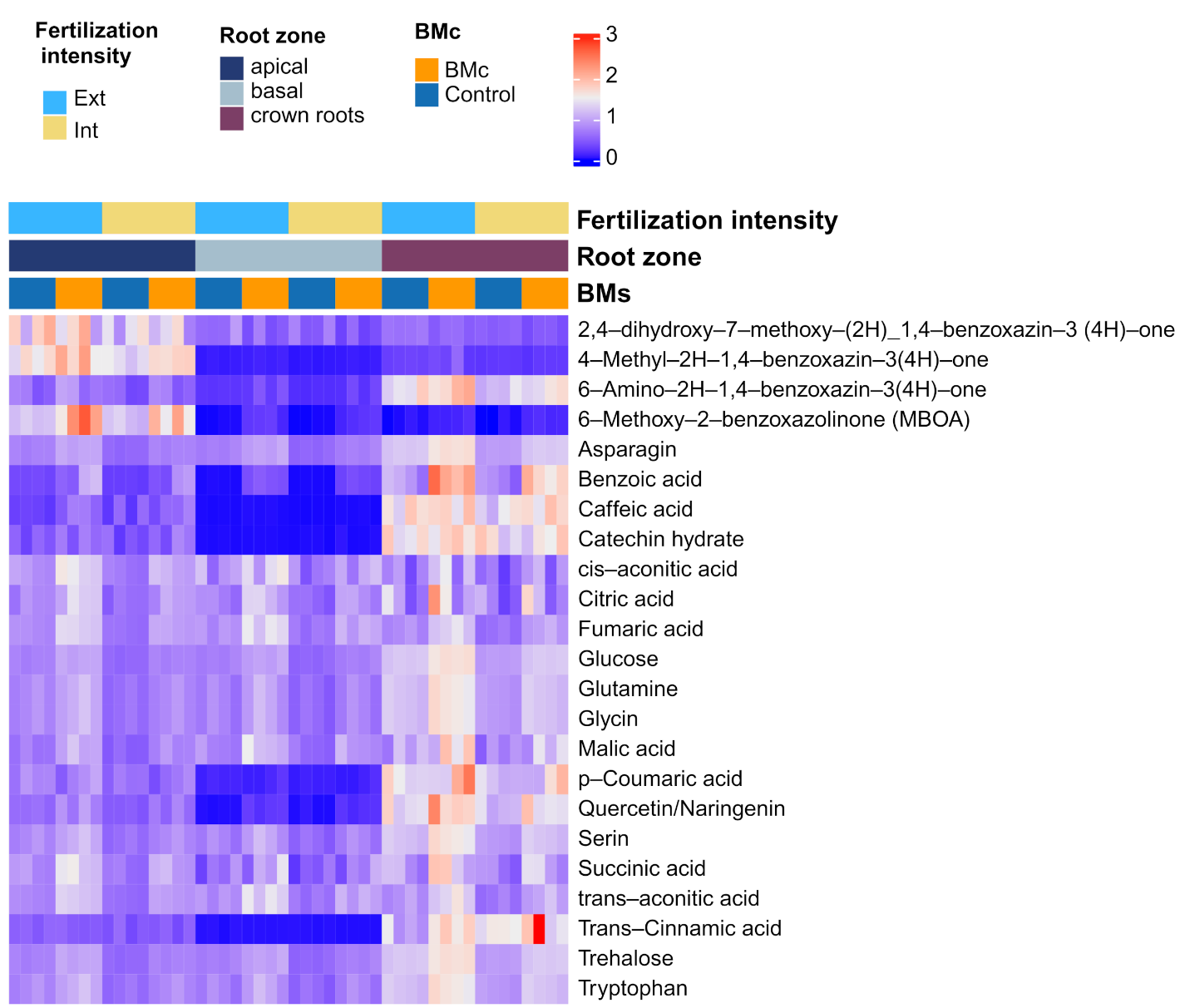


**Fig. S6.** Heatmap of the concentrations of low-molecular weight rhizosphere metabolites across three root zones of maize (*Zea mays* cv. Benedictio) grown under different fertilization intensities and inoculation treatments. The colour code indicates the accumulation intensity from low (blue) to high (red) concentrations. BMc: Beneficial microorganisms consortium. Ctrl: Control. Ext-extensive N-fertilization. Int- intensive N-fertilization.


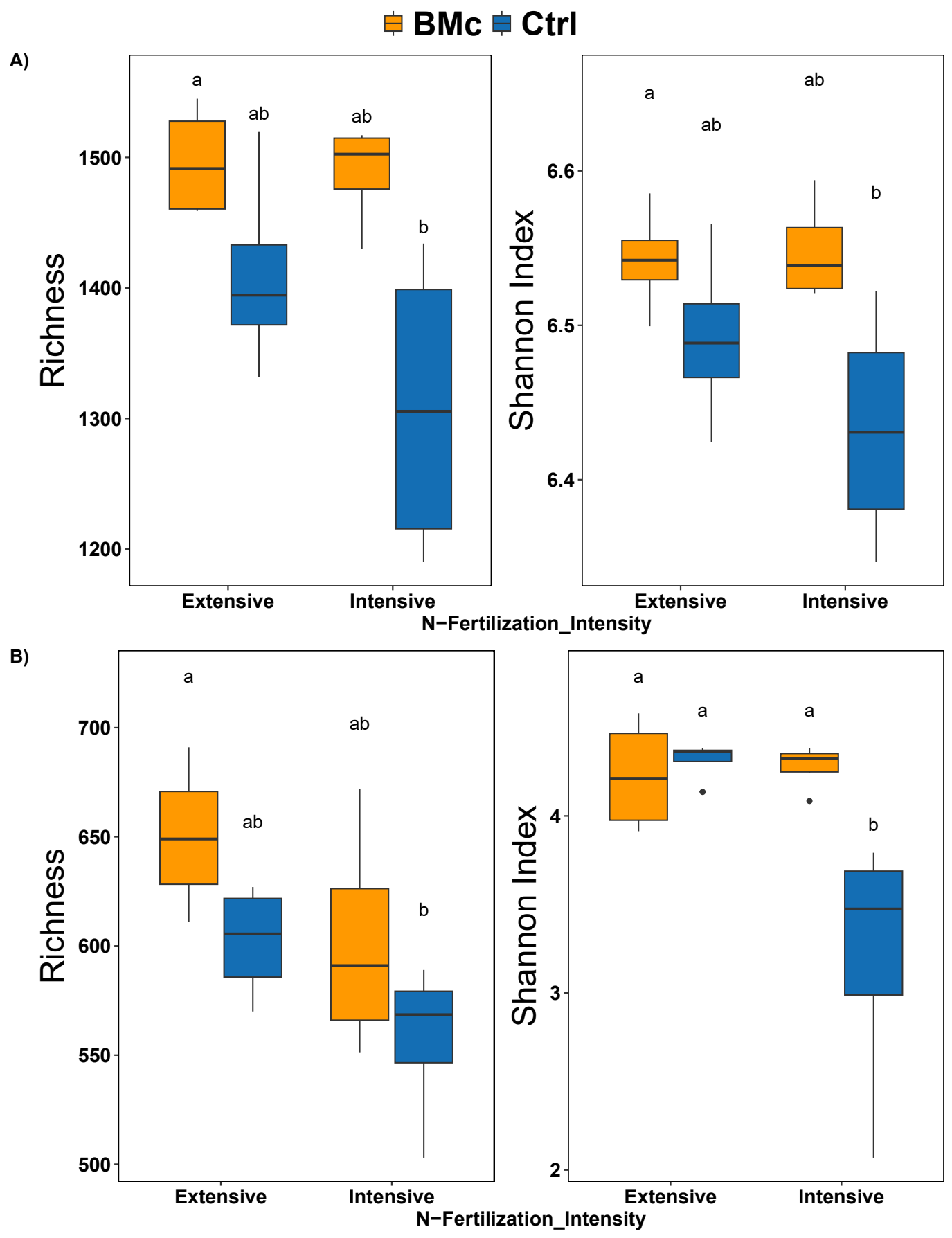


**Fig. S7.** Alpha-diversity plots (Richness and Shannon Index) of bacterial (A) and fungal (B) communities. Different letters indicate significant differences at p<0.05 according to pairwise Wilcoxon’s rank-sum test and Benjamini-Hochberg correction. BMc: Beneficial microorganisms consortium. Ctrl: Control.


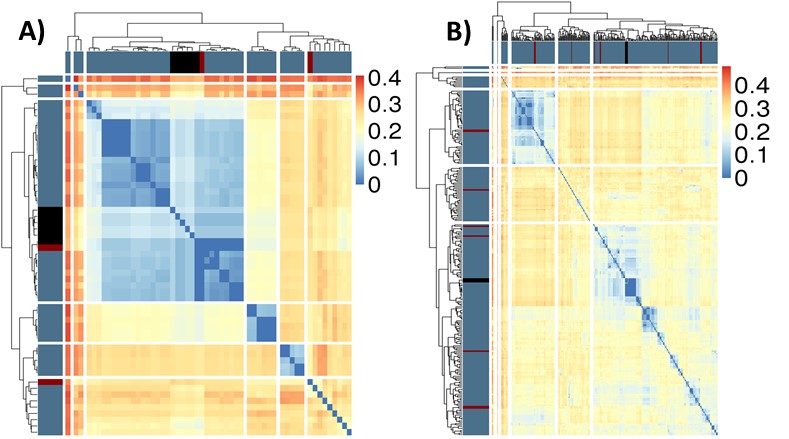


**Fig. S8.** Clustering based on Hamming distance (100-Similarity%)^^2^ of *Bacillus* ASVs (A) and *Trichoderma* ASVs (B), shown in red color, with the respective BMc members (*Bacillus atrophaeus* ABi03 or *Trichoderma* OMG16) shown with black color and *Bacillus* or *Trichoderma* type strains from NCBI shown with blue color. The pairwise sequence alignment was performed with the 16S rRNA gene for bacterial ASVs and the ITS2 region for fungal ASVs. The color scale of the heatmap shows the sequence dissimilarity based on alignments (perfect match=0%).

**
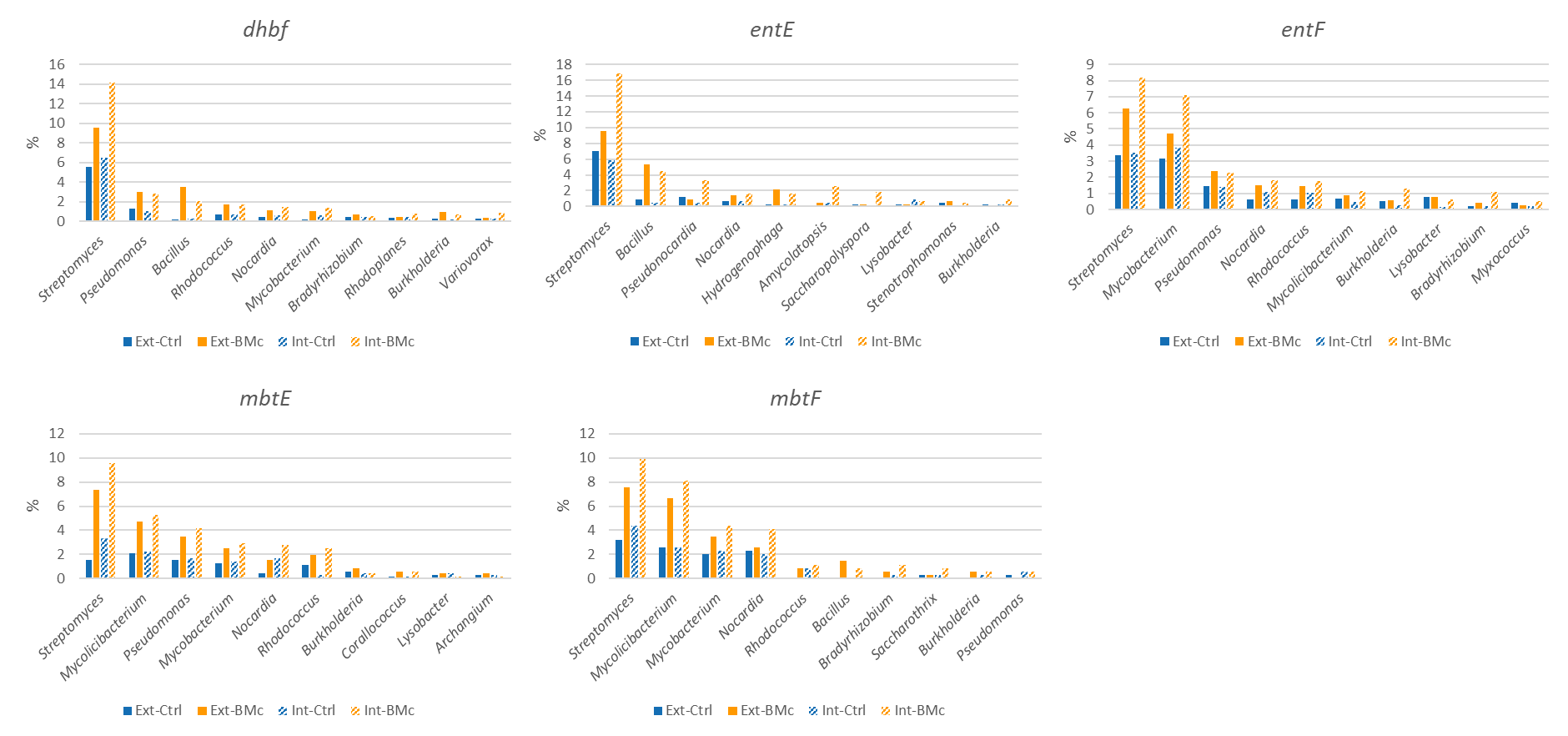
**

**Fig. S9.** The relative abundance of differential abundant siderophore-related genes and their association with bacterial genera. BMc: Beneficial microorganisms consortium. Ctrl: Control. Ext-extensive N-fertilization. Int- intensive N-fertilization.

**
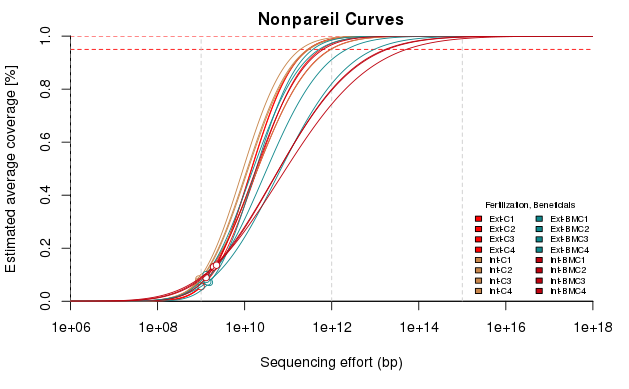
**

**Fig. S10.** Nonpareil-curve for coverage of diversity for the metagenomic reads.

**
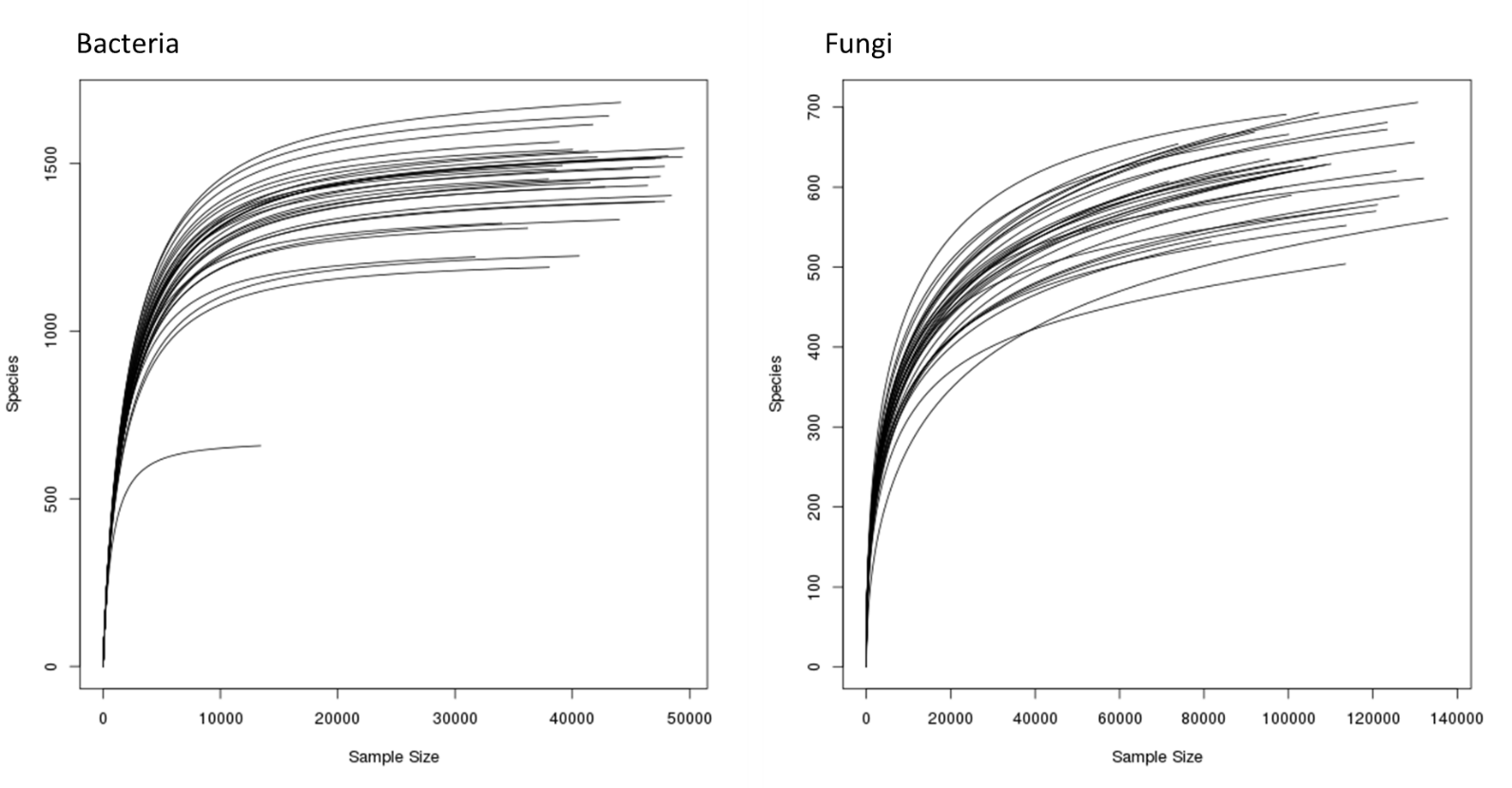
**

**Fig. S11.** Rarefaction curves for diversity coverage through amplicon sequencing of 16S rRNA gene (Bacteria) or ITS2 fragments (Fungi).
