# Supplementary material for: Microbial inoculants modulate the rhizosphere microbiome, alleviate plant stress responses, and enhance maize growth at field scale
Source: Genome Biol. 2025 Jun 1;26:148. doi: 10.1186/s13059-025-03621-7 (PMC12128319; doi:10.1186/s13059-025-03621-7)
Supplement: Supplementary file 2 — Additional file 2: Table S1 Two-way ANOVA test of the main effect and interactions between different long-term N-fertilization practices and application of beneficial microorganism consortium on the nutrient content of maize shoots. Table S2 Effect of BMc inoculation on the chemical properties of soils with different long-term N-fertilization intensities. Table S3 List of plant genes used for gene expression analyses, along with their annotation and PCR primers. Table S4 Detected phenolic compounds in the rhizosphere of maize inoculated with a consortium of beneficial microorganisms. Table S5 Bacterial ASVs that passed the differential abundance testingfor extensive or intensive fertilization. Table S6 Fungal ASVs that passed the differential abundance testingfor extensive or intensive fertilization. Table S7 Detailed information on metagenomics sequences annotated to ABi03, RU47, and OMG16 genomes. Table S8 List of potential plant-beneficial functions of rhizosphere microorganisms in a customized database, established including protein sequences downloaded from online Kyoto Encyclopedia of Genes and GenomesOrthology database. Table S9 Variables of the two main modulesof the integrated network. Table S10 HPLC conditions for determination of phenolic compounds in rhizosphere soil solutions of maize. [file 13059_2025_3621_MOESM2_ESM.docx]

**Supplementary material: Tables**

**Table S1.** Two-way ANOVA test of the main effect and interactions between different long-term N-fertilization practices (Fert.) and application of beneficial microorganisms consortium (BMc) on the nutrient content of maize shoots.

|  | **F-value_Fert._** | **P-value_Fert._** | **F-value_BMc_** | **P-value_BMc_** | **F-value_Fert x BMc_** | **P-value_Fert x BMc_** |
| --- | --- | --- | --- | --- | --- | --- |
| N | 4.60 | 0.068 | 4.16 | 0.071 | 0.37 | 0.52 |
| P | 8.08 | 0.008 | 0.77 | 0.324 | 0.44 | 0.448 |
| K | 2.04 | 0.102 | 0.18 | 0.602 | 1.77 | 0.124 |
| Mg | 22.27 | 0.002 | 1.47 | 0.287 | 1.95 | 0.224 |
| S | 13.34 | 0.008 | 10.97 | 0.013 | 1.29 | 0.314 |
| Fe | 2.86 | 0.079 | 17.14 | 0.001 | 0.00 | 0.954 |
| Cu | 9.65 | 0.015 | 2.68 | 0.148 | 0.34 | 0.59 |
| Zn | 10.25 | 0.009 | 0.23 | 0.01 | 0.08 | 0.184 |
| Mn | 1.20 | 0.03 | 0.92 | 0.042 | 0.23 | 0.1 |

**Table S2.** Effect of BMc inoculation on the chemical properties of soils with different long-term N-fertilization intensities. Nutrient composition of soils with different N-fertilization intensities (extensive (Ext) and intensive (Int)) after beneficial microorganisms consortium (BMc) inoculation. Root-associated soil of control (Ctrl) and inoculated plants (BMc) was sampled at a depth of 0-30 cm. Abbreviations: total carbon (TC), total nitrogen (TN), dry matter (DM), organic matter (OM). Values represent means ± standard deviation of four replicates. Letters denote significant differences among the treatments (Tukey- post-hoc test; *p*≤0.05).

|  | **Ctrl-Ext** | **BMc-Ext** | **Ctrl-Ext** | **BMc-Ext** |
| --- | --- | --- | --- | --- |
| **pH** | 7.5±0 | 7.5±0 | 7.4±0.1 | 7.4±0.1 |
| **C/N** | 11.5±0.6 | 11.5±0.6 | 11.3±0.5 | 11.5±0.6 |
| **TC(%)** | 2±0.1 | 2±0.1 | 2.1±0.1 | 2.1±0.1 |
| **TN(%)** | 0.17±0.01 ab | 0.16±0.01b | 0.18±0a | 0.17±0.01ab |
| **DM(%)** | 82.2±1.4 | 79.9±1.7 | 83.9±3.0 | 82.9±3.4 |
| **OM(%)** | 3.4±0.1 ab | 3.2±0.1b | 3.5±0.1a | 3.5±0.1a |
| **NO3-N (mg 100 g^-1^  soil)** | 0.42±0.16b | 0.47±0.08ab | 0.49±0.04ab | 0.81±0.32a |
| **NH4-N (mg 100 g^-1^  soil)** | 0.03±0.01b | 0.03±0.01b | 0.06±0.01a | 0.02±0.01b |
| **K (mg 100 g^-1^  soil)** | 21.3±0.8 | 19.3±0.5 | 20.3±1.8 | 19.5±1.3 |
| **Mg (mg 100 g^-1^  soil)** | 8.4±0.2 | 8.7±0.4 | 9.3±1.1 | 9.7±1.0 |
| **P (mg 100 g^-1^  soil)** | 8.7±0.5 | 10.3±1.7 | 9.3±1.0 | 10.5±2.00 |
| **Ca (mg 100 g^-1^  soil)** | 271±5 | 274±6 | 268±7 | 273±9 |
| **Fe (mg 100 g^-1^  soil)** | 2.2±0.1 | 2.5±0.4 | 2.4±0.4 | 2.5±0.5 |
| **Cu (mg kg^-1^  soil)** | 2.5±0.1 | 2.6±0.1 | 2.6±0.2 | 2.7±0.3 |
| **Mn (mg kg^-1^  soil)^##^** | 119±8 | 114±12 | 142±39 | 148±51 |
| **Na (mg kg^-1^  soil)** | 10±1.4b | 11.3±1.0a | 8.8±0.5b | 10.8±0.5a |
| **Zn (mg kg^-1^  soil)** | 3.4±0.2 | 3.6±0.3 | 3.6±0.2 | 3.7±0.4 |

**Table S3.** List of plant genes used for gene expression analyses, along with their annotation and PCR primers.

| **Gene** | **Annotation** | **FW Primer (5’-3’)** | **REV Primer (5’-3’)** |
| --- | --- | --- | --- |
| *PR1* | (a)biotic stress | AACCTTCTTGGCACCACCCT | GTTGGTGTCGTGGTCGTAGT |
| *PR4* | (a)biotic stress | TGATGGATAGATGGCGATTGC | AGAATTGACACCGCCAAACC |
| *SOD2* | (a)biotic stress | CACCAACGGCTGCATGTC | ATGCTCCTTGCCAACAGGAT |
| *ACS6* | (a)biotic stress | GTGCTCATCACCAACCCTTC | ACGAAGTCCACCAGCATCTC |
| *LOX1* | (a)biotic stress | CACTCGAGCTCGTCAAGGAT | TCCAACCTGTCTTGTCCTCTTT |
| *OPR8* | (a)biotic stress | AAGAGCAGACTGATGCATGG | ATATTGGAGCAGAACCACCC |
| *Def1* | (a)biotic stress | TGCTGCTCCTCATCGTCGTTGC | TTGCCGCCGCCGTAGCCTTC |
| *Def2* | (a)biotic stress | AGTCCAGGGCGACCGTGTG | CGAGTGGTGCTGGCTCTTGC |
| *APX1* | (a)biotic stress | GCTCTGTCTTGCATGGCACTCC | GATGGGCTCTAGCAACCTGACG |
| *GSR1* | (a)biotic stress | CCAATAGGGTCAACCTGACACCAG | TCCATACTCTTCAATTGCCTGCTC |
| *MPK3* | (a)biotic stress | ACAGCGACATGATGACGGAGTA | CCAATCACCTCGGTTATGAG |
| *SOD4* | (a)biotic stress | CGTGTTGCTTGTGGGATCATCG | TGTTATTCTAGCCTGCTGGTGACG |
| *WRKY106* | (a)biotic stress | GCTCGTCACCTACACCTTCG | AGCTTTCGTCCTCCTCTGC |
| *WRKY40* | (a)biotic stress | CTACTTCCGCTGCTCCTTCG | TGCTGCTGCTGGTGCTGCT |
| *WRKY17* | (a)biotic stress | TTTTTCTTCTTCCGCTGTTCTACTC | TCAGATCGAGGGTCGTCATCT |
| *WRKY33* | (a)biotic stress | GTGGTCCAGACGATGAGCGACAT | GCTGCTCAGCATCTCCAGGGTGT |
| *WRKY58* | (a)biotic stress | AGGAAGTGGAGGAGGCGAACA | GGATGGCTTGCGCTTGC |
| *ERF1* | (a)biotic stress | ACTTCCCCAGCGACACCTC | TGACCTCGTCGGACACCTGA |
| *EREB58* | (a)biotic stress | GACGGCGACAAGAAGCGA | CGGTGCCAGGACGACG |
| *NAS3* | Fe/Zn-uptake | GGCTCACCAGAAGATGGAGGAG | TCACGCATGTGGTGTAGACACG |
| *NAS4* | Fe/Zn-uptake | CACGGCACACACCACAAGCAACAAG | ATCCATGCGGTGTGGGCACATAGAC |
| *IRTa* | Fe/Zn-uptake | CTGCAGAGCAGCGTCAGG | AGTACTGTGTCATGTCGTCTC |
| *OPT8a* | Fe/Zn-uptake | GCTACATGAGCATGTCGCAGGCT | TGCCAGCCACAATGGTACCAACAAACTGA |
| *OPT8b* | Fe/Zn-uptake | GGCTACATGAGCATGGCACAGG | CCAGCCACAATGGTACCGACAAACTGG |
| *DCT2* | (a)biotic stress | TTCTTGCAGTCTCACTATGGAT | GTTTAGCACTCCAAGCACA |
| *AMT1* | N-uptake | CCAGCAGCCAGGTGTAAAA | CGACTCCCAAGTAGCCAAG |
| *GS2* | N metabolism | TATAAACCGGTCCGCGACA | CGATGAATCAAAGACAGCCGT |
| *NIR* | N metabolism | AGGTGGCGGACATCGGCTTC | ACGGCACGGACTTCCTGTAGAC |
| *NR1* | N metabolism | AGGTGGCGGACATCGGCTTC | ACGGCACGGACTTCCTGTAGAC |
| *NR2* | N metabolism | ACTGGTGCTGGTGCTTCTGGTCC | ATGCCGATCTCGCCCTTGTGC |
| *NAR2.2* | N-uptake | GCTGGAGGTGACCCTCTGCTACG | TGCCGGGCGATCCTGAACTGG |
| *MYB30* | (a)biotic stress | CTCCTTGTCGTTGTCCCTCT | CTTGCTTGTGCTTGAGGTGT |
| *MYB36* | (a)biotic stress | GGTGTTCGAGTACGAGACGA | ACAGGACGGTGGAAGTGG |
| *MYB95* | (a)biotic stress | CTCGTCTTCTCTCCGCTACC | TAGTCGACGACAACGAGTGG |
| *Pht1* | P-uptake | CGTAGTACGTGTGTGATAGTCTGG | TATTATCACACGTGGACCTCTACC |
| *Pht3* | P-uptake | GCCTTCCGTTACGTCATTGT | AGCACGTCTCTGATCCCATC |
| *Pht4* | P-uptake | ACCGGCTACCCTCACCTACT | CTACCTTCTTGGCGTCCTTG |
| *Pht8* | P-uptake | CCTGGAGGAGATGTTCAGGA | AAGACGGTGAACCAGTAGCC |
| *Pht9* | P-uptake | CATTGTCACGCTCGTCATCT | GGTGGAGTTGAAGTGGTCGT |

**Table S4.** Detected phenolic compounds in the rhizosphere of maize (cv. Benedictio) inoculated with a consortium of beneficial microorganisms (BMc).

| **No.** | **Compound** | **Retention time [min]** | **Molecular weight (g/mol)** | **[M+H]+ ions** | **[M-H]- ions** | **Molecular formula** |
| --- | --- | --- | --- | --- | --- | --- |
| 1 | Caffeine | 9.16 | 194 | 195.08757 |  | C_8_H_10_N_4_O_2_ |
| 2 | Naringenin | 23.21 | 272 |  | 271.06091 | C_15_H_12_O_5_ |
| 3 | Apigenin | 23.11 | 270 | 271.05988 | 269.04529 | C_15_H_10_O_5_ |
| 4 | Caffeic acid | 8.45 | 180 |  | 179.03401 | C_9_H_8_O_4_ |
| 5 | Protocatechuic acid | 5.63 | 154 |  | 153.01814 | C_7_H_6_O_4_ |
| 6 | Quercetin | 21.51 | 302 | 303.04980 | 301.03506 | C_15_H_10_O_7_ |
| 7 | Kämpherol | 23.22 | 286 | 287.05499 | 285.04016 | C_15_H_10_O_6_ |
| 8 | Catechin | 7.79 | 290 | 291.08627 | 289.07156 | C_15_H_14_O_6_ |
| 9 | Gallic acid | 3.91 | 170 |  | 169.01323 | C_7_H_6_O_5_ |
| 10 | MBOA | 13.61 | 165 | 166.04990 | 164.03419 | C_8_H_7_NO_3_ |
| 11 | 6-Amino-2*H*-1,4-benzoxazin-3(4*H*)-one | 3.15 | 164 | 165.06589 |  | C_8_H_8_N_2_O_2_ |
| 12 | 4-Methyl-2H-1,4-benzoxazin-3(4H)-one | 19.46 | 163 | 164.07060 |  | C_9_H_9_NO_2_ |
| 13 | 6-Acetyl-2H-1,4-benzoxazin-3(4H)-one | 13.14 | 191 | 192.06552 | 190.05003 | C_10_H_9_NO_3_ |
| 14 | 2H-1,4-benzoxazin-3(4H)-one | 12.49 | 149 | 150.05501 |  | C_8_H_7_NO_2_ |
| 15 | 2HMBOA | 17.6 | 163 | 164.07059 |  | C_9_H_9_NO_2_ |
| 16 | Ferulic acid | 12.68 | 194 |  | 193.0497 | C_10_H_10_O_4_ |
| 17 | Genistein | 23.1 | 270 | 271.05972 | 269.04556 | C_15_H_10_O_5_ |
| 18 | p-Coumaric acid | 11.17 | 164 |  | 163.03896 | C_9_H_8_O_3_ |
| 19 | Quercetin-3-glucoside | 11.93 | 464 | 465.10303 | 463.08780 | C_21_H_20_O_12_ |
| 20 | Curcumin | 26.11 | 368 | 369.13342 | 367.11841 | C_21_H_20_O_6_ |
| 21 | Cinnamic acid | 20.52 | 148 | 149.05980 | 147.04401 | C_9_H_8_O_2_ |
| 22 | Vanillic acid | 8.61 | 168 |  | 167.03398 | C_8_H_8_O_4_ |
| 23 | 4,4-Dihydroxy-2-methoxy-chalcone | 23.12 | 270 | 271.09619 | 269.08148 | C_16_H_14_O_4_ |
| 24 | Quercetin-glucuronide | 12.33 | 478 | 479.08206 | 477.06702 | C_21_H_18_O_13_ |

**Table S5.** Bacterial ASVs that passed the differential abundance testing (BMc vs. Ctrl) for extensive (Ext) or intensive (Int) fertilization (FERT). The differential abundance was tested via logistic regression and *p-*value correction. Mean relative abundances (%) and standard deviation are provided. The β-coefficient (β) of the model indicates the size of association of each ASV with BMc inoculation. BMc: Beneficial microorganisms consortium, Ctrl: Control.

| **ASV** | **FERT** | **Ctrl** | **BMc** | **β** |
| --- | --- | --- | --- | --- |
| *Acidobacteriota;Blastocatellia;Pyrinomonadales;Pyrinomonadaceae;RB41;ASV2846* | Int | 0+0 | 0.012+0.004 | 6802.29 |
| *Acidobacteriota;Vicinamibacteria;Vicinamibacterales;Vicinamibacteraceae;Unclassified;ASV2616* | Int | 0+0 | 0.023+0.01 | 4446.13 |
| *Acidobacteriota;Vicinamibacteria;Vicinamibacterales;Vicinamibacteraceae;Unclassified;ASV703* | Int | 0.027+0.018 | 0.066+0.011 | 3618.23 |
| *Acidobacteriota;Vicinamibacteria;Vicinamibacterales;Vicinamibacteraceae;Unclassified;ASV720* | Int | 0.015+0.018 | 0.05+0.014 | 115463.56 |
| *Acidobacteriota;Vicinamibacteria;Vicinamibacterales;Vicinamibacteraceae;Unclassified;ASV783* | Int | 0.009+0.01 | 0.063+0.017 | 1566.03 |
| *Acidobacteriota;Vicinamibacteria;Vicinamibacterales;Vicinamibacteraceae;Unclassified;ASV902* | Int | 0+0 | 0.056+0.018 | 1214.06 |
| *Actinobacteriota;Acidimicrobiia;Microtrichales;Ilumatobacteraceae;Ilumatobacter;ASV74* | Int | 0.34+0.034 | 0.233+0.013 | -768.56 |
| *Actinobacteriota;Acidimicrobiia;Microtrichales;Ilumatobacteraceae;Unclassified;ASV110* | Int | 0.263+0.041 | 0.167+0.032 | -2490.41 |
| *Actinobacteriota;Acidimicrobiia;Microtrichales;Ilumatobacteraceae;Unclassified;ASV1274* | Int | 0.046+0.013 | 0.024+0.007 | -215320.31 |
| *Actinobacteriota;Acidimicrobiia;Microtrichales;Ilumatobacteraceae;Unclassified;ASV233* | Int | 0.21+0.018 | 0.118+0.025 | -945.97 |
| *Actinobacteriota;Acidimicrobiia;Microtrichales;Ilumatobacteraceae;Unclassified;ASV316* | Int | 0.174+0.006 | 0.118+0.007 | -1207.68 |
| *Actinobacteriota;Actinobacteria;Corynebacteriales;Nocardiaceae;Nocardia;ASV206* | Int | 0.236+0.042 | 0.149+0.032 | -2895.38 |
| *Actinobacteriota;Actinobacteria;Frankiales;Geodermatophilaceae;Unclassified;ASV3487* | Int | 0.015+0.008 | 0.003+0.003 | -27451.4 |
| *Actinobacteriota;Actinobacteria;Micrococcales;Cellulomonadaceae;Cellulomonas;ASV620* | Int | 0.019+0.022 | 0.081+0.018 | 2760.91 |
| *Actinobacteriota;Actinobacteria;Micrococcales;Intrasporangiaceae;Intrasporangium;ASV331* | Int | 0.043+0.035 | 0.112+0.029 | 263734.3 |
| *Actinobacteriota;Actinobacteria;Micrococcales;Microbacteriaceae;Agromyces;ASV3* | Int | 0.705+0.064 | 1.084+0.131 | 312.09 |
| *Actinobacteriota;Actinobacteria;Micrococcales;Microbacteriaceae;Microbacterium;ASV338* | Int | 0.054+0.037 | 0.11+0.011 | 2723.33 |
| *Actinobacteriota;Actinobacteria;Micrococcales;Microbacteriaceae;Unclassified;ASV247* | Int | 0.078+0.053 | 0.148+0.007 | 1719.91 |
| *Actinobacteriota;Actinobacteria;Micrococcales;Micrococcaceae;Paenarthrobacter;ASV994* | Int | 0.03+0.011 | 0.067+0.013 | 10452.8 |
| *Actinobacteriota;Actinobacteria;Micrococcales;Promicromonosporaceae;Promicromonospora;ASV81* | Int | 0.169+0.026 | 0.287+0.09 | 1023.65 |
| *Actinobacteriota;Actinobacteria;Micromonosporales;Micromonosporaceae;Catellatospora;ASV165* | Int | 0.27+0.008 | 0.236+0.017 | -3854.54 |
| *Actinobacteriota;Actinobacteria;Propionibacteriales;Nocardioidaceae;Actinopolymorpha;ASV1319* | Int | 0.041+0.011 | 0.012+0.008 | -3439.82 |
| *Actinobacteriota;Actinobacteria;Propionibacteriales;Nocardioidaceae;Kribbella;ASV134* | Int | 0.246+0.052 | 0.082+0.056 | -687.62 |
| *Actinobacteriota;Actinobacteria;Propionibacteriales;Nocardioidaceae;Kribbella;ASV327* | Int | 0.159+0.04 | 0.015+0.029 | -654.94 |
| *Actinobacteriota;Actinobacteria;Propionibacteriales;Nocardioidaceae;Kribbella;ASV372* | Int | 0.212+0.053 | 0.056+0.067 | -1272.72 |
| *Actinobacteriota;Actinobacteria;Propionibacteriales;Nocardioidaceae;Nocardioides;ASV192* | Int | 0.283+0.012 | 0.177+0.021 | -678.37 |
| *Actinobacteriota;Actinobacteria;Propionibacteriales;Nocardioidaceae;Nocardioides;ASV252* | Int | 0.237+0.029 | 0.159+0.034 | -6828.07 |
| *Actinobacteriota;Actinobacteria;Propionibacteriales;Nocardioidaceae;Nocardioides;ASV383* | Int | 0.219+0.012 | 0.132+0.019 | -854.58 |
| *Actinobacteriota;Actinobacteria;Propionibacteriales;Nocardioidaceae;Nocardioides;ASV475* | Int | 0.114+0.017 | 0.083+0.009 | -4604.18 |
| *Actinobacteriota;Actinobacteria;Propionibacteriales;Nocardioidaceae;Nocardioides;ASV573* | Int | 0.128+0.032 | 0.075+0.007 | -48932.76 |
| *Actinobacteriota;Actinobacteria;Propionibacteriales;Nocardioidaceae;Unclassified;ASV668* | Int | 0.095+0.025 | 0.057+0.038 | -177264.66 |
| *Actinobacteriota;Actinobacteria;Streptomycetales;Streptomycetaceae;Streptomyces;ASV1054* | Int | 0.079+0.011 | 0.057+0.007 | -6392.28 |
| *Actinobacteriota;Actinobacteria;Streptomycetales;Streptomycetaceae;Streptomyces;ASV25* | Int | 0.804+0.061 | 0.548+0.083 | -1072.81 |
| *Actinobacteriota;Actinobacteria;Streptomycetales;Streptomycetaceae;Streptomyces;ASV255* | Int | 0.169+0.017 | 0.095+0.064 | -2611.68 |
| *Actinobacteriota;Actinobacteria;Streptomycetales;Streptomycetaceae;Streptomyces;ASV27* | Int | 1.039+0.193 | 0.528+0.048 | -168.29 |
| *Actinobacteriota;Actinobacteria;Streptomycetales;Streptomycetaceae;Streptomyces;ASV58* | Int | 0.454+0.066 | 0.321+0.043 | -915.56 |
| *Actinobacteriota;Actinobacteria;Streptomycetales;Streptomycetaceae;Streptomyces;ASV78* | Int | 0.384+0.046 | 0.306+0.013 | -2319.93 |
| *Actinobacteriota;Actinobacteria;Streptomycetales;Streptomycetaceae;Unclassified;ASV149* | Int | 0.236+0.019 | 0.079+0.02 | -384.84 |
| *Actinobacteriota;Actinobacteria;Streptomycetales;Streptomycetaceae;Unclassified;ASV820* | Int | 0.078+0.026 | 0.03+0.02 | -5459.81 |
| *Actinobacteriota;Rubrobacteria;Rubrobacterales;Rubrobacteriaceae;Rubrobacter;ASV112* | Int | 0.289+0.052 | 0.189+0.021 | -1084.41 |
| *Actinobacteriota;Thermoleophilia;Gaiellales;Gaiellaceae;Gaiella;ASV148* | Int | 0.172+0.016 | 0.242+0.031 | 3001.1 |
| *Actinobacteriota;Thermoleophilia;Gaiellales;Gaiellaceae;Gaiella;ASV155* | Int | 0.069+0.006 | 0.19+0.016 | 466.32 |
| *Actinobacteriota;Thermoleophilia;Gaiellales;Gaiellaceae;Gaiella;ASV2274* | Int | 0.028+0.008 | 0.003+0.005 | -6864.27 |
| *Actinobacteriota;Thermoleophilia;Gaiellales;Gaiellaceae;Gaiella;ASV329* | Int | 0.049+0.01 | 0.106+0.018 | 1730.85 |
| *Actinobacteriota;Thermoleophilia;Gaiellales;Gaiellaceae;Gaiella;ASV556* | Int | 0.036+0.011 | 0.088+0.012 | 1613.37 |
| *Actinobacteriota;Thermoleophilia;Gaiellales;Gaiellaceae;Gaiella;ASV571* | Int | 0.029+0.033 | 0.082+0.013 | 7155.25 |
| *Actinobacteriota;Thermoleophilia;Solirubrobacterales;67-14;Unclassified;ASV531* | Int | 0.101+0.009 | 0.066+0.007 | -3399.93 |
| *Actinobacteriota;Thermoleophilia;Solirubrobacterales;Solirubrobacteraceae;Solirubrobacter;ASV590* | Int | 0.082+0.021 | 0.056+0.004 | -12536.21 |
| *Actinobacteriota;Thermoleophilia;Solirubrobacterales;Solirubrobacteraceae;Solirubrobacter;ASV686* | Int | 0.04+0.006 | 0.05+0.001 | 55834.22 |
| *Bacteroidota;Bacteroidia;Chitinophagales;Chitinophagaceae;Chitinophaga;ASV831* | Int | 0.015+0.017 | 0.045+0.013 | 11378.16 |
| *Bacteroidota;Bacteroidia;Chitinophagales;Chitinophagaceae;Flavisolibacter;ASV2259* | Int | 0.007+0.014 | 0.032+0.004 | 108183.91 |
| *Bacteroidota;Bacteroidia;Chitinophagales;Chitinophagaceae;Terrimonas;ASV1826* | Int | 0.001+0.003 | 0.01+0.003 | 15107.16 |
| *Bacteroidota;Bacteroidia;Cytophagales;Microscillaceae;Ohtaekwangia;ASV1702* | Int | 0.006+0.013 | 0.043+0.022 | 18470.75 |
| *Bacteroidota;Bacteroidia;Sphingobacteriales;Sphingobacteriaceae;Arcticibacter;ASV1227* | Int | 0.023+0.015 | 0.071+0.03 | 3370.23 |
| *Chloroflexi;Chloroflexia;Thermomicrobiales;AKYG1722;Unclassified;ASV266* | Int | 0.033+0.009 | 0.129+0.014 | 741.19 |
| *Chloroflexi;Chloroflexia;Thermomicrobiales;AKYG1722;Unclassified;ASV964* | Int | 0.003+0.006 | 0.036+0.009 | 3020.68 |
| *Chloroflexi;Chloroflexia;Thermomicrobiales;JG30-KF-CM45;Unclassified;ASV101* | Int | 0.059+0.002 | 0.261+0.028 | 273.44 |
| *Chloroflexi;Chloroflexia;Thermomicrobiales;JG30-KF-CM45;Unclassified;ASV1081* | Int | 0+0 | 0.043+0.004 | 1204.57 |
| *Chloroflexi;Chloroflexia;Thermomicrobiales;JG30-KF-CM45;Unclassified;ASV1772* | Int | 0+0 | 0.024+0.003 | 2250.3 |
| *Chloroflexi;Chloroflexia;Thermomicrobiales;JG30-KF-CM45;Unclassified;ASV187* | Int | 0.066+0.034 | 0.165+0.024 | 2227.28 |
| *Chloroflexi;Chloroflexia;Thermomicrobiales;JG30-KF-CM45;Unclassified;ASV1935* | Int | 0.003+0.007 | 0.032+0.005 | 3692.79 |
| *Chloroflexi;Chloroflexia;Thermomicrobiales;JG30-KF-CM45;Unclassified;ASV365* | Int | 0.025+0.019 | 0.123+0.044 | 1482.22 |
| *Chloroflexi;Chloroflexia;Thermomicrobiales;JG30-KF-CM45;Unclassified;ASV38* | Int | 0.086+0.059 | 0.441+0.063 | 198.18 |
| *Chloroflexi;Chloroflexia;Thermomicrobiales;JG30-KF-CM45;Unclassified;ASV417* | Int | 0.044+0.034 | 0.096+0.012 | 3647.85 |
| *Chloroflexi;Chloroflexia;Thermomicrobiales;JG30-KF-CM45;Unclassified;ASV458* | Int | 0.013+0.015 | 0.09+0.011 | 879.94 |
| *Chloroflexi;Chloroflexia;Thermomicrobiales;JG30-KF-CM45;Unclassified;ASV611* | Int | 0+0 | 0.058+0.019 | 1255.82 |
| *Chloroflexi;Chloroflexia;Thermomicrobiales;JG30-KF-CM45;Unclassified;ASV706* | Int | 0+0 | 0.045+0.013 | 1734.58 |
| *Chloroflexi;Chloroflexia;Thermomicrobiales;JG30-KF-CM45;Unclassified;ASV873* | Int | 0.008+0.016 | 0.057+0.009 | 3156.82 |
| *Chloroflexi;Chloroflexia;Thermomicrobiales;JG30-KF-CM45;Unclassified;ASV899* | Int | 0.009+0.011 | 0.05+0.015 | 5712.56 |
| *Chloroflexi;Chloroflexia;Thermomicrobiales;JG30-KF-CM45;Unclassified;ASV909* | Int | 0.01+0.019 | 0.064+0.007 | 2640.44 |
| *Firmicutes;Bacilli;Bacillales;Bacillaceae;Bacillus;ASV83* | Int | 0.414+0.06 | 0.529+0.069 | 2378.81 |
| *Firmicutes;Bacilli;Bacillales;Bacillaceae;Unclassified;ASV418* | Int | 0.123+0.014 | 0.092+0.028 | -189909.86 |
| *Firmicutes;Bacilli;Bacillales;Bacillaceae;Unclassified;ASV82* | Int | 0.421+0.056 | 0.317+0.036 | -89416.43 |
| *Gemmatimonadota;Gemmatimonadetes;Gemmatimonadales;Gemmatimonadaceae;Unclassified;ASV103* | Int | 0.297+0.046 | 0.174+0.013 | -1216.06 |
| *Gemmatimonadota;Gemmatimonadetes;Gemmatimonadales;Gemmatimonadaceae;Unclassified;ASV126* | Int | 0.262+0.006 | 0.179+0.03 | -860.21 |
| *Gemmatimonadota;Gemmatimonadetes;Gemmatimonadales;Gemmatimonadaceae;Unclassified;ASV362* | Int | 0.13+0.04 | 0.018+0.035 | -1626.18 |
| *Gemmatimonadota;Gemmatimonadetes;Gemmatimonadales;Gemmatimonadaceae;Unclassified;ASV54* | Int | 0.399+0.06 | 0.295+0.023 | -1677.02 |
| *Nitrospirota;Nitrospiria;Nitrospirales;Nitrospiraceae;Nitrospira;ASV97* | Int | 0.274+0.019 | 0.216+0.014 | -1658.35 |
| *Proteobacteria;Alphaproteobacteria;Dongiales;Dongiaceae;Dongia;ASV928* | Int | 0.013+0.009 | 0.044+0.011 | 6365.76 |
| *Proteobacteria;Alphaproteobacteria;Micropepsales;Micropepsaceae;Unclassified;ASV434* | Int | 0.058+0.01 | 0.087+0.01 | 25071.49 |
| *Proteobacteria;Alphaproteobacteria;Rhizobiales;Beijerinckiaceae;Microvirga;ASV402* | Int | 0.081+0.021 | 0.138+0.016 | 3673.56 |
| *Proteobacteria;Alphaproteobacteria;Rhizobiales;Devosiaceae;Devosia;ASV1069* | Int | 0.037+0.025 | 0.07+0.017 | 43614.01 |
| *Proteobacteria;Alphaproteobacteria;Rhizobiales;Rhizobiaceae;Allorhizobium-Neorhizobium-Pararhizobium-Rhizobium;ASV1528* | Int | 0.004+0.009 | 0.045+0.008 | 2562.32 |
| *Proteobacteria;Alphaproteobacteria;Rhizobiales;Rhizobiaceae;Mesorhizobium;ASV1103* | Int | 0.049+0.003 | 0.011+0.021 | -12182.32 |
| *Proteobacteria;Alphaproteobacteria;Rhizobiales;Rhizobiaceae;Neorhizobium;ASV685* | Int | 0.083+0.014 | 0.06+0.004 | -6298.13 |
| *Proteobacteria;Alphaproteobacteria;Rhizobiales;Rhizobiaceae;Phyllobacterium;ASV34* | Int | 0.821+0.182 | 0.528+0.053 | -499.09 |
| *Proteobacteria;Alphaproteobacteria;Rhizobiales;Rhizobiaceae;Unclassified;ASV137* | Int | 0.315+0.029 | 0.244+0.032 | -61610.05 |
| *Proteobacteria;Alphaproteobacteria;Rhizobiales;Rhizobiaceae;Unclassified;ASV1664* | Int | 0+0 | 0.038+0.016 | 2248.45 |
| *Proteobacteria;Alphaproteobacteria;Rhizobiales;Rhizobiales_Incertae_Sedis;Bauldia;ASV1484* | Int | 0.007+0.015 | 0.067+0.026 | 5166.41 |
| *Proteobacteria;Alphaproteobacteria;Rhizobiales;Rhizobiales_Incertae_Sedis;Nordella;ASV125* | Int | 0.188+0.01 | 0.121+0.022 | -1283.53 |
| *Proteobacteria;Alphaproteobacteria;Rhizobiales;Xanthobacteraceae;Unclassified;ASV1467* | Int | 0+0 | 0.031+0.003 | 1741.24 |
| *Proteobacteria;Alphaproteobacteria;Sphingomonadales;Sphingomonadaceae;Sphingomonas;ASV1005* | Int | 0.124+0.023 | 0+0 | -528.86 |
| *Proteobacteria;Alphaproteobacteria;Sphingomonadales;Sphingomonadaceae;Sphingomonas;ASV460* | Int | 0.146+0.007 | 0.119+0.022 | -10673.12 |
| *Proteobacteria;Alphaproteobacteria;Tistrellales;Geminicoccaceae;Unclassified;ASV405* | Int | 0.084+0.007 | 0.103+0.007 | 6486.31 |
| *Proteobacteria;Gammaproteobacteria;Burkholderiales;Burkholderiaceae;Ralstonia;ASV299* | Int | 0.078+0.019 | 0.138+0.043 | 4880.94 |
| *Proteobacteria;Gammaproteobacteria;Burkholderiales;Comamonadaceae;Ramlibacter;ASV987* | Int | 0.035+0.006 | 0.056+0.015 | 9573.47 |
| *Proteobacteria;Gammaproteobacteria;Burkholderiales;Nitrosomonadaceae;Ellin6067;ASV1999* | Int | 0.003+0.003 | 0.012+0.004 | 999571.41 |
| *Proteobacteria;Gammaproteobacteria;Xanthomonadales;Xanthomonadaceae;Arenimonas;ASV1337* | Int | 0.011+0.008 | 0.032+0.009 | 26055.66 |
| *Proteobacteria;Gammaproteobacteria;Xanthomonadales;Xanthomonadaceae;Arenimonas;ASV3464* | Int | 0.001+0.001 | 0.007+0.002 | 18759.03 |
| *Proteobacteria;Gammaproteobacteria;Xanthomonadales;Xanthomonadaceae;Lysobacter;ASV839* | Int | 0.045+0.003 | 0.026+0.007 | -6046.62 |
| *Proteobacteria;Gammaproteobacteria;Xanthomonadales;Xanthomonadaceae;Stenotrophomonas;ASV563* | Int | 0.071+0.033 | 0.011+0.013 | -2035.65 |
| *Verrucomicrobiota;Verrucomicrobiae;Verrucomicrobiales;Rubritaleaceae;Luteolibacter;ASV1997* | Int | 0.022+0.013 | 0.01+0.003 | -39406.83 |
| *Verrucomicrobiota;Verrucomicrobiae;Verrucomicrobiales;Rubritaleaceae;Luteolibacter;ASV3626* | Int | 0.014+0.014 | 0+0 | -17257.31 |
| *Acidobacteriota;Vicinamibacteria;Vicinamibacterales;Vicinamibacteraceae;Unclassified;ASV387* | Ext | 0.07+0.048 | 0.137+0.016 | 17597.24 |
| *Acidobacteriota;Vicinamibacteria;Vicinamibacterales;Vicinamibacteraceae;Unclassified;ASV392* | Ext | 0.068+0.012 | 0.093+0.019 | 49642.47 |
| *Actinobacteriota;Acidimicrobiia;Microtrichales;Ilumatobacteraceae;Unclassified;ASV1248* | Ext | 0.048+0.023 | 0.006+0.012 | -6421.33 |
| *Actinobacteriota;Actinobacteria;Micrococcales;Microbacteriaceae;Agromyces;ASV3* | Ext | 1.174+0.061 | 0.95+0.034 | -337.85 |
| *Actinobacteriota;Actinobacteria;Micrococcales;Microbacteriaceae;Microbacterium;ASV228* | Ext | 0.175+0.015 | 0.121+0.024 | -10023.68 |
| *Actinobacteriota;Actinobacteria;Micrococcales;Micrococcaceae;Pseudarthrobacter;ASV18* | Ext | 0.675+0.083 | 0.513+0.021 | -678.96 |
| *Actinobacteriota;Actinobacteria;Micrococcales;Micrococcaceae;Pseudarthrobacter;ASV2* | Ext | 3.162+0.346 | 2.324+0.12 | -97.16 |
| *Actinobacteriota;Actinobacteria;Micrococcales;Micrococcaceae;Pseudarthrobacter;ASV5* | Ext | 1.402+0.132 | 1.027+0.071 | -219.42 |
| *Actinobacteriota;Actinobacteria;Micrococcales;Micrococcaceae;Unclassified;ASV24* | Ext | 0.958+0.25 | 0.522+0.075 | -280.61 |
| *Actinobacteriota;Actinobacteria;Propionibacteriales;Nocardioidaceae;Actinopolymorpha;ASV1319* | Ext | 0.038+0.008 | 0.005+0.01 | -5118.11 |
| *Actinobacteriota;Actinobacteria;Propionibacteriales;Nocardioidaceae;Kribbella;ASV372* | Ext | 0.099+0.045 | 0+0 | -1393.82 |
| *Actinobacteriota;Actinobacteria;Propionibacteriales;Nocardioidaceae;Nocardioides;ASV170* | Ext | 0.12+0.057 | 0.218+0.028 | 2905.3 |
| *Actinobacteriota;Actinobacteria;Propionibacteriales;Nocardioidaceae;Nocardioides;ASV2094* | Ext | 0+0 | 0.011+0.008 | 22643.45 |
| *Actinobacteriota;Actinobacteria;Pseudonocardiales;Pseudonocardiaceae;Actinophytocola;ASV3416* | Ext | 0+0 | 0.014+0.003 | 4684.17 |
| *Actinobacteriota;Actinobacteria;Pseudonocardiales;Pseudonocardiaceae;Pseudonocardia;ASV1204* | Ext | 0.016+0.011 | 0.036+0.006 | 8108.58 |
| *Actinobacteriota;Actinobacteria;Streptomycetales;Streptomycetaceae;Streptomyces;ASV131* | Ext | 0.358+0.076 | 0.242+0.017 | -1603.29 |
| *Actinobacteriota;Actinobacteria;Streptomycetales;Streptomycetaceae;Streptomyces;ASV25* | Ext | 0.655+0.046 | 0.504+0.036 | -819.82 |
| *Actinobacteriota;Actinobacteria;Streptomycetales;Streptomycetaceae;Streptomyces;ASV53* | Ext | 0.503+0.039 | 0.314+0.045 | -476.15 |
| *Actinobacteriota;Actinobacteria;Streptomycetales;Streptomycetaceae;Streptomyces;ASV58* | Ext | 0.378+0.055 | 0.277+0.027 | -1475.77 |
| *Actinobacteriota;Actinobacteria;Streptomycetales;Streptomycetaceae;Unclassified;ASV149* | Ext | 0.205+0.072 | 0.047+0.012 | -997.45 |
| *Actinobacteriota;Actinobacteria;Streptomycetales;Streptomycetaceae;Unclassified;ASV820* | Ext | 0.086+0.025 | 0.049+0.006 | -2852.94 |
| *Actinobacteriota;Rubrobacteria;Rubrobacterales;Rubrobacteriaceae;Rubrobacter;ASV332* | Ext | 0.193+0.032 | 0.144+0.02 | -18151.23 |
| *Actinobacteriota;Rubrobacteria;Rubrobacterales;Rubrobacteriaceae;Rubrobacter;ASV449* | Ext | 0.073+0.013 | 0.056+0.007 | -50966.43 |
| *Actinobacteriota;Thermoleophilia;Solirubrobacterales;67-14;Unclassified;ASV1824* | Ext | 0.025+0.008 | 0.003+0.006 | -7598.51 |
| *Actinobacteriota;Thermoleophilia;Solirubrobacterales;67-14;Unclassified;ASV2003* | Ext | 0.039+0.015 | 0+0 | -1627.53 |
| *Actinobacteriota;Thermoleophilia;Solirubrobacterales;67-14;Unclassified;ASV237* | Ext | 0.197+0.029 | 0.126+0.013 | -1345.18 |
| *Actinobacteriota;Thermoleophilia;Solirubrobacterales;67-14;Unclassified;ASV264* | Ext | 0.169+0.02 | 0.135+0.002 | -3683.17 |
| *Actinobacteriota;Thermoleophilia;Solirubrobacterales;67-14;Unclassified;ASV49* | Ext | 0.47+0.136 | 0.298+0.023 | -1716.68 |
| *Actinobacteriota;Thermoleophilia;Solirubrobacterales;67-14;Unclassified;ASV531* | Ext | 0.089+0.012 | 0.064+0.008 | -9147.45 |
| *Actinobacteriota;Thermoleophilia;Solirubrobacterales;67-14;Unclassified;ASV5555* | Ext | 0+0 | 0.007+0.005 | 11272.69 |
| *Actinobacteriota;Thermoleophilia;Solirubrobacterales;67-14;Unclassified;ASV888* | Ext | 0.08+0.018 | 0.027+0.032 | -6172.57 |
| *Actinobacteriota;Thermoleophilia;Solirubrobacterales;Solirubrobacteraceae;Conexibacter;ASV472* | Ext | 0.159+0.036 | 0+0 | -418.76 |
| *Actinobacteriota;Thermoleophilia;Solirubrobacterales;Solirubrobacteraceae;Solirubrobacter;ASV1032* | Ext | 0.056+0.013 | 0.023+0.015 | -4172.63 |
| *Actinobacteriota;Thermoleophilia;Solirubrobacterales;Solirubrobacteraceae;Solirubrobacter;ASV2951* | Ext | 0.016+0.006 | 0+0 | -4234.96 |
| *Actinobacteriota;Thermoleophilia;Solirubrobacterales;Solirubrobacteraceae;Unclassified;ASV4027* | Ext | 0+0 | 0.012+0.008 | 21705.33 |
| *Bacteroidota;Bacteroidia;Chitinophagales;Chitinophagaceae;Flaviaesturariibacter;ASV5688* | Ext | 0+0 | 0.006+0.004 | 22859.89 |
| *Bacteroidota;Bacteroidia;Chitinophagales;Chitinophagaceae;Flavisolibacter;ASV3557* | Ext | 0.003+0.006 | 0.021+0.006 | 14153.13 |
| *Bacteroidota;Bacteroidia;Chitinophagales;Chitinophagaceae;Niastella;ASV4530* | Ext | 0+0 | 0.012+0.004 | 5626.63 |
| *Bacteroidota;Bacteroidia;Cytophagales;Microscillaceae;Unclassified;ASV976* | Ext | 0.008+0.011 | 0.03+0.008 | 266653.68 |
| *Bacteroidota;Bacteroidia;Cytophagales;Spirosomaceae;Dyadobacter;ASV1269* | Ext | 0.006+0.013 | 0.035+0.009 | 133941.08 |
| *Firmicutes;Bacilli;Bacillales;Bacillaceae;Bacillus;ASV20* | Ext | 0.095+0.067 | 2.403+0.831 | 32.82 |
| *Firmicutes;Bacilli;Bacillales;Bacillaceae;Unclassified;ASV82* | Ext | 0.443+0.043 | 0.334+0.025 | -774.36 |
| *Firmicutes;Bacilli;Paenibacillales;Paenibacillaceae;Paenibacillus;ASV1729* | Ext | 0.032+0.009 | 0.015+0.007 | -16858.59 |
| *Gemmatimonadota;Gemmatimonadetes;Gemmatimonadales;Gemmatimonadaceae;Gemmatimonas;ASV4320* | Ext | 0.002+0.002 | 0.013+0.011 | 25859.48 |
| *Gemmatimonadota;Gemmatimonadetes;Gemmatimonadales;Gemmatimonadaceae;Unclassified;ASV569* | Ext | 0.069+0.025 | 0.026+0.019 | -13961.99 |
| *Myxococcota;Myxococcia;Myxococcales;Myxococcaceae;Unclassified;ASV3115* | Ext | 0+0 | 0.012+0.014 | 22206.92 |
| *Proteobacteria;Alphaproteobacteria;Dongiales;Dongiaceae;Dongia;ASV2640* | Ext | 0.007+0.009 | 0.024+0.005 | 36136.21 |
| *Proteobacteria;Alphaproteobacteria;Rhizobiales;Beijerinckiaceae;Microvirga;ASV216* | Ext | 0.076+0.036 | 0.182+0.009 | 860.18 |
| *Proteobacteria;Alphaproteobacteria;Rhizobiales;Beijerinckiaceae;Microvirga;ASV240* | Ext | 0.102+0.034 | 0.169+0.006 | 1615.42 |
| *Proteobacteria;Alphaproteobacteria;Rhizobiales;Beijerinckiaceae;Microvirga;ASV48* | Ext | 0.215+0.107 | 0.442+0.047 | 1532.87 |
| *Proteobacteria;Alphaproteobacteria;Rhizobiales;Beijerinckiaceae;Microvirga;ASV51* | Ext | 0.208+0.082 | 0.375+0.042 | 1095.88 |
| *Proteobacteria;Alphaproteobacteria;Rhizobiales;Beijerinckiaceae;Psychroglaciecola;ASV360* | Ext | 0.062+0.018 | 0.13+0.018 | 1743.56 |
| *Proteobacteria;Alphaproteobacteria;Rhizobiales;Beijerinckiaceae;Unclassified;ASV315* | Ext | 0.106+0.047 | 0.173+0.013 | 6259.64 |
| *Proteobacteria;Alphaproteobacteria;Rhizobiales;Devosiaceae;Devosia;ASV751* | Ext | 0+0 | 0.086+0.02 | 793.6 |
| *Proteobacteria;Alphaproteobacteria;Rhizobiales;Labraceae;Labrys;ASV1432* | Ext | 0.012+0.014 | 0.034+0.006 | 23412.78 |
| *Proteobacteria;Alphaproteobacteria;Rhizobiales;Methyloligellaceae;Unclassified;ASV256* | Ext | 0.06+0.07 | 0.144+0.012 | 4328.68 |
| *Proteobacteria;Alphaproteobacteria;Rhizobiales;Rhizobiaceae;Mesorhizobium;ASV1103* | Ext | 0.02+0.024 | 0.078+0.021 | 3193.37 |
| *Proteobacteria;Alphaproteobacteria;Rhizobiales;Rhizobiaceae;Pseudaminobacter;ASV1440* | Ext | 0+0 | 0.049+0.022 | 1423.04 |
| *Proteobacteria;Alphaproteobacteria;Rhizobiales;Rhizobiaceae;Unclassified;ASV514* | Ext | 0.071+0.036 | 0.164+0.037 | 2408.01 |
| *Proteobacteria;Alphaproteobacteria;Rhizobiales;Xanthobacteraceae;Pseudorhodoplanes;ASV580* | Ext | 0.043+0.033 | 0.107+0.019 | 19221.58 |
| *Proteobacteria;Alphaproteobacteria;Sphingomonadales;Sphingomonadaceae;Sphingomonas;ASV140* | Ext | 0.138+0.081 | 0.266+0.018 | 2387.48 |
| *Proteobacteria;Alphaproteobacteria;Sphingomonadales;Sphingomonadaceae;Sphingomonas;ASV1407* | Ext | 0.005+0.01 | 0.041+0.016 | 11485.87 |
| *Proteobacteria;Alphaproteobacteria;Sphingomonadales;Sphingomonadaceae;Sphingomonas;ASV157* | Ext | 0.087+0.064 | 0.184+0.017 | 2499.47 |
| *Proteobacteria;Alphaproteobacteria;Sphingomonadales;Sphingomonadaceae;Sphingomonas;ASV214* | Ext | 0.08+0.053 | 0.183+0.039 | 1380.66 |
| *Proteobacteria;Alphaproteobacteria;Sphingomonadales;Sphingomonadaceae;Sphingomonas;ASV56* | Ext | 0.249+0.151 | 0.478+0.037 | 1505.37 |
| *Proteobacteria;Alphaproteobacteria;Tistrellales;Geminicoccaceae;Candidatus_Alysiosphaera;ASV384* | Ext | 0.045+0.04 | 0.172+0.018 | 786.59 |
| *Proteobacteria;Alphaproteobacteria;Tistrellales;Geminicoccaceae;Candidatus_Alysiosphaera;ASV403* | Ext | 0.056+0.039 | 0.118+0.021 | 7380.26 |
| *Proteobacteria;Alphaproteobacteria;Tistrellales;Geminicoccaceae;Unclassified;ASV1606* | Ext | 0.033+0.006 | 0.009+0.007 | -4525.48 |
| *Proteobacteria;Gammaproteobacteria;Burkholderiales;Comamonadaceae;Ramlibacter;ASV1659* | Ext | 0+0 | 0.031+0.01 | 2557.18 |
| *Proteobacteria;Gammaproteobacteria;Burkholderiales;Comamonadaceae;Rhizobacter;ASV1406* | Ext | 0.027+0.031 | 0.075+0.012 | 18837.19 |
| *Proteobacteria;Gammaproteobacteria;Burkholderiales;Comamonadaceae;Unclassified;ASV1510* | Ext | 0+0 | 0.038+0.013 | 1924.39 |
| *Proteobacteria;Gammaproteobacteria;Burkholderiales;Comamonadaceae;Unclassified;ASV2377* | Ext | 0+0 | 0.019+0.009 | 5760.7 |
| *Proteobacteria;Gammaproteobacteria;Burkholderiales;Comamonadaceae;Unclassified;ASV2584* | Ext | 0+0 | 0.024+0.006 | 2992.34 |
| *Proteobacteria;Gammaproteobacteria;Burkholderiales;Comamonadaceae;Variovorax;ASV894* | Ext | 0.018+0.021 | 0.063+0.012 | 2357.71 |
| *Proteobacteria;Gammaproteobacteria;Burkholderiales;Nitrosomonadaceae;Ellin6067;ASV3221* | Ext | 0+0 | 0.011+0.003 | 5643.5 |
| *Proteobacteria;Gammaproteobacteria;Burkholderiales;Nitrosomonadaceae;Ellin6067;ASV350* | Ext | 0.128+0.036 | 0.091+0.008 | -18454.48 |
| *Proteobacteria;Gammaproteobacteria;Burkholderiales;Oxalobacteraceae;Unclassified;ASV3687* | Ext | 0+0 | 0.014+0.004 | 4705.91 |
| *Proteobacteria;Gammaproteobacteria;Burkholderiales;SC-I-84;Unclassified;ASV1129* | Ext | 0.046+0.014 | 0+0 | -1658.84 |
| *Proteobacteria;Gammaproteobacteria;Pseudomonadales;Pseudomonadaceae;Pseudomonas;ASV1083* | Ext | 0.032+0.01 | 0.055+0.013 | 16077.35 |
| *Proteobacteria;Gammaproteobacteria;Steroidobacterales;Steroidobacteraceae;Unclassified;ASV2811* | Ext | 0.002+0.002 | 0.011+0.002 | 13362.96 |
| *Proteobacteria;Gammaproteobacteria;Xanthomonadales;Rhodanobacteraceae;Unclassified;ASV448* | Ext | 0.058+0.027 | 0.11+0.017 | 79149.57 |

**Table S6.** Fungal ASVs that passed the differential abundance testing (BMc vs Ctrl) for extensive (Ext) or intensive (Int) fertilization (FERT). The differential abundance was tested via logistic regression and *p-*value correction. Mean relative abundances (%) and standard deviation are provided. The β-coefficient of the model indicates the size of association of each ASV with BMc inoculation. BMc: Beneficial microorganisms consortium, Ctrl: Control.

| **ASV** | **FERT** | **Ctrl** | **BMc** | | **β** |
| --- | --- | --- | --- | --- | --- |
| *Dichotomopilus;ASV8* | Int | 0.0144+0.0173 | 0.0002+0.0004 | -57139.22 | |
| *Chaetomiaceae_gen_Incertae_sedis;ASV13* | Int | 0.0015+0.003 | 0.0467+0.0086 | 1559.88 | |
| *Sporobolomyces;ASV15* | Int | 0.3047+0.1568 | 0.1122+0.046 | -167.4 | |
| *Hirsutella;ASV24* | Int | 0.0013+0.002 | 0.0255+0.0174 | 4825.44 | |
| *Rhizophydiales_gen_Incertae_sedis;ASV68* | Int | 0.0056+0.0045 | 0+0 | | -3604.52 |
| *Rozellomycota_gen_Incertae_sedis;ASV72* | Int | 0.0028+0.0024 | 0.0202+0.0117 | | 9885.19 |
| *Glomus;ASV74* | Int | 0.0007+0.0005 | 0.031+0.0209 | | 4025.64 |
| *Glomus;ASV75* | Int | 0+0 | 0.0124+0.0171 | | 17455.91 |
| *Mortierella;ASV90* | Int | 0.0014+0.0012 | 0.0056+0.0022 | | 2024.26 |
| *Mortierella;ASV91* | Int | 0.0918+0.0162 | 0.3601+0.2017 | | 160.19 |
| *Pyrenochaeta;ASV99* | Int | 0.004+0.006 | 0.0263+0.0149 | | 240.75 |
| *Pezizales_gen_Incertae_sedis;ASV102* | Int | 0.0011+0.0013 | 0.0062+0.0026 | | 1597.72 |
| *Betamyces;ASV117* | Int | 0.0421+0.0176 | 0.0105+0.0075 | | -82330.95 |
| *Betamyces;ASV120* | Int | 0.0025+0.0038 | 0.0245+0.0128 | | 9138.66 |
| *Chytridiomycota_gen_Incertae_sedis;ASV121* | Int | 0.0531+0.0061 | 0.0265+0.0095 | | -4534 |
| *Lecythophora;ASV137* | Int | 0.0038+0.0037 | 0.0245+0.0142 | | 46876.04 |
| *Coniochaeta;ASV142* | Int | 0.0124+0.0043 | 0.0745+0.0352 | | 2278.81 |
| *Sclerostagonospora;ASV148* | Int | 0.0301+0.0137 | 1.4254+0.5914 | | 52.09 |
| *Talaromyces;ASV149* | Int | 0.6569+0.3345 | 0.0588+0.045 | | -154.35 |
| *Hypocreales_gen_Incertae_sedis;ASV171* | Int | 0+0 | 0.0473+0.0542 | | 11301 |
| *Onygenales_gen_Incertae_sedis;ASV177* | Int | 0+0 | 0.0231+0.0331 | | 12787.9 |
| *Xylariales_gen_Incertae_sedis;ASV178* | Int | 0.0043+0.0055 | 0+0 | | -9949.41 |
| *Sporormiella;ASV194* | Int | 0.0135+0.0115 | 0.5339+0.3851 | | 286.02 |
| *Preussia;ASV195* | Int | 0.7256+0.1475 | 0.2414+0.0396 | | -178.69 |
| *Sporormiaceae_gen_Incertae_sedis;ASV197* | Int | 0.0492+0.0286 | 0.7648+0.741 | | 258.05 |
| *Gymnoascus;ASV198* | Int | 0.154+0.0523 | 0.0536+0.0164 | | -1087.33 |
| *Leucothecium;ASV205* | Int | 0.4151+0.244 | 0.0963+0.0365 | | -635.72 |
| *Aaosphaeria;ASV207* | Int | 0.0183+0.0137 | 0.0954+0.0106 | | 992.09 |
| *Rozellomycota_gen_Incertae_sedis;ASV208* | Int | 0+0 | 0.008+0.0069 | | 3679.82 |
| *Gibellulopsis;ASV245* | Int | 0.1092+0.0511 | 0.357+0.1604 | | 1804.39 |
| *Aspergillus;ASV252* | Int | 0.0214+0.0117 | 0.004+0.0035 | | -268.8 |
| *Plenodomus;ASV263* | Int | 0+0 | 0.0044+0.0038 | | 6456.87 |
| *Monographella;ASV276* | Int | 0.0002+0.0004 | 0.0047+0.0037 | | 253830.05 |
| *Ascomycota_gen_Incertae_sedis;ASV291* | Int | 0.1442+0.0968 | 0.5981+0.2516 | | 18.79 |
| *Periconia;ASV292* | Int | 0.0132+0.0136 | 0.1098+0.0492 | | 3598.28 |
| *Onygenales_gen_Incertae_sedis;ASV297* | Int | 0.0032+0.0025 | 0+0 | | -6169.7 |
| *Fusidium;ASV307* | Int | 0+0 | 0.0039+0.0032 | | 8023.28 |
| *Lobulomycetales_gen_Incertae_sedis;ASV310* | Int | 0.0002+0.0005 | 0.0053+0.0036 | | 3885.85 |
| *Thelebolaceae_gen_Incertae_sedis;ASV327* | Int | 0.2237+0.1209 | 0.077+0.0191 | | -2878.52 |
| *Pseudocoleophoma;ASV341* | Int | 0.0056+0.0056 | 0+0 | | -9865.13 |
| *Cephalotrichiella;ASV342* | Int | 0.0215+0.0106 | 0.0038+0.0044 | | -423.4 |
| *Articulospora;ASV349* | Int | 0.0005+0.0005 | 0.0043+0.0031 | | 4044.05 |
| *Trichoderma;ASV354* | Int | 0.4537+0.2596 | 2.7963+1.1019 | | 56.48 |
| *Sordariales_gen_Incertae_sedis;ASV365* | Int | 0.0492+0.0213 | 0.0002+0.0004 | | -2513.81 |
| *Claroideoglomus;ASV370* | Int | 0.0047+0.0041 | 0+0 | | -6690.49 |
| *Schizothecium;ASV382* | Int | 0.0253+0.0119 | 0.1008+0.0608 | | 2075.99 |
| *Sordariales_gen_Incertae_sedis;ASV390* | Int | 0.1136+0.0343 | 0.0283+0.0133 | | -1235.81 |
| *Neoschizothecium;ASV392* | Int | 0.0419+0.0336 | 0.2499+0.1811 | | 1147.76 |
| *Hypocreales_gen_Incertae_sedis;ASV396* | Int | 1.1678+0.2815 | 0.2856+0.1954 | | -97.52 |
| *Sordariales_gen_Incertae_sedis;ASV400* | Int | 0.0512+0.0262 | 1.1241+1.021 | | 198.6 |
| *Nectriaceae_gen_Incertae_sedis;ASV404* | Int | 0.6533+0.1576 | 0.161+0.0577 | | -210.71 |
| *Sordariales_gen_Incertae_sedis;ASV406* | Int | 0.0087+0.0124 | 0+0 | | -44215.51 |
| *Hypocreales_gen_Incertae_sedis;ASV411* | Int | 0.0029+0.0021 | 0.0882+0.0828 | | 3210.21 |
| *Hypocreales_gen_Incertae_sedis;ASV412* | Int | 0.0187+0.0033 | 0.044+0.0149 | | 7166.23 |
| *Hypocreales_gen_Incertae_sedis;ASV413* | Int | 0.0011+0.0013 | 0.0132+0.007 | | 19161.05 |
| *Valsonectria;ASV417* | Int | 0+0 | 0.0131+0.0135 | | 4795.05 |
| *Sordariales_gen_Incertae_sedis;ASV425* | Int | 0.0014+0.0018 | 0.005+0.0008 | | 156158.32 |
| *Sordariales_gen_Incertae_sedis;ASV426* | Int | 0.0141+0.009 | 0.4146+0.6395 | | 755.02 |
| *Glomeraceae_gen_Incertae_sedis;ASV435* | Int | 0.0004+0.0005 | 0.0642+0.0332 | | 1493.94 |
| *Hypholoma;ASV459* | Int | 0+0 | 0.0061+0.007 | | 8045.6 |
| *Hypocreales_gen_Incertae_sedis;ASV479* | Int | 0.0029+0.0021 | 0.1106+0.1316 | | 1756.99 |
| *Pyrenochaetopsis;ASV507* | Int | 0.0211+0.0112 | 0.0804+0.0296 | | 2492.6 |
| *Pleosporales_gen_Incertae_sedis;ASV515* | Int | 0.0023+0.0034 | 0.0192+0.0117 | | 339.81 |
| *Vishniacozyma;ASV525* | Int | 0.0062+0.0042 | 0+0 | | -45803.84 |
| *Ramophialophora;ASV529* | Int | 0.0928+0.1558 | 0.0015+0.0018 | | -604.07 |
| *Hypocreales_gen_Incertae_sedis;ASV532* | Int | 0+0 | 0.0075+0.004 | | 12355.64 |
| *Hypocreaceae_gen_Incertae_sedis;ASV535* | Int | 0.003+0.0016 | 0.0382+0.0231 | | 6886.94 |
| *Ceratobasidiaceae_gen_Incertae_sedis;ASV537* | Int | 0+0 | 0.0099+0.0092 | | 49308.96 |
| *Actinomucor;ASV539* | Int | 0.0027+0.0025 | 0.0274+0.0112 | | 8264.89 |
| *Actinomucor;ASV540* | Int | 0.0065+0.0082 | 0.0598+0.028 | | 4787.13 |
| *Typhula;ASV550* | Int | 0.0002+0.0004 | 0.0068+0.0055 | | 17145.67 |
| *Agaricomycetes_gen_Incertae_sedis;ASV552* | Int | 0.0106+0.0045 | 0.0186+0.0028 | | 15971.13 |
| *Cheilymenia;ASV567* | Int | 0.0249+0.0242 | 0.0016+0.0032 | | -9920.77 |
| *Halosphaeriaceae_gen_Incertae_sedis;ASV579* | Int | 0.3391+0.1226 | 0.004+0.0022 | | -301.22 |
| *Hymenoscyphus;ASV582* | Int | 0+0 | 0.0126+0.011 | | 1867.98 |
| *Ascomycota_gen_Incertae_sedis;ASV595* | Int | 0.0185+0.0082 | 2.8622+4.61 | | 97.11 |
| *Magnaporthales_gen_Incertae_sedis;ASV596* | Int | 0.0141+0.0086 | 0.0871+0.0282 | | 1224.28 |
| *Ceratobasidium;ASV609* | Int | 0.0004+0.0005 | 0.0133+0.0051 | | 9301.98 |
| *Mucor;ASV625* | Int | 0.1506+0.0755 | 0.0462+0.0172 | | -2667.33 |
| *Tranzscheliella;ASV638* | Int | 0.0026+0.0043 | 0.0291+0.0181 | | 786.25 |
| *Absidia;ASV641* | Int | 0.0002+0.0004 | 0.0167+0.0185 | | 543.63 |
| *Chytridiomycota_gen_Incertae_sedis;ASV659* | Int | 0.0161+0.006 | 0.0028+0.0057 | | -914.66 |
| *Fungi_gen_Incertae_sedis;ASV660* | Int | 0.0054+0.0047 | 0+0 | | -6975.79 |
| *Rhizophlyctis;ASV661* | Int | 0.0018+0.0008 | 0.1343+0.1346 | | 19308.92 |
| *Tausonia;ASV682* | Int | 0.2676+0.0725 | 0.5551+0.1754 | | 6586.34 |
| *Exophiala;ASV687* | Int | 0.006+0.0084 | 0.0004+0.0005 | | -348262.81 |
| *Acremonium;ASV689* | Int | 0.0412+0.0153 | 0.6798+0.6201 | | 680.8 |
| *Stachybotryaceae_gen_Incertae_sedis;ASV693* | Int | 0.0008+0.0007 | 0.0936+0.0683 | | 1145.91 |
| *Albifimbria;ASV696* | Int | 0.0011+0.0014 | 0.0149+0.0119 | | 13906.19 |
| *Paramicrothyrium;ASV703* | Int | 0.0071+0.0012 | 0.166+0.1102 | | 636.24 |
| *Clonostachys;ASV713* | Int | 0.1068+0.1118 | 0.0262+0.0155 | | -132.9 |
| *Ascomycota_gen_Incertae_sedis;ASV716* | Int | 0.1714+0.0681 | 1.477+0.8432 | | 139.81 |
| *Tetracladium;ASV727* | Int | 0.095+0.0353 | 0.2109+0.0703 | | 1625.59 |
| *Tetracladium;ASV732* | Int | 0.0022+0.0017 | 0.0613+0.0201 | | 1290.05 |
| *Ambispora;ASV735* | Int | 0.0086+0.0046 | 0.0866+0.0454 | | 1521.36 |
| *Fusarium;ASV740* | Int | 0.0089+0.0073 | 0.0427+0.019 | | 4525.44 |
| *Lophiotrema;ASV749* | Int | 0+0 | 0.0037+0.003 | | 60699.91 |
| *Alternaria;ASV750* | Int | 0.0036+0.0029 | 0.0183+0.0173 | | 983.53 |
| *Neosetophoma;ASV786* | Int | 0.0532+0.0476 | 0.3051+0.0917 | | 645.98 |
| *Ophiosphaerella;ASV789* | Int | 0.0822+0.0709 | 0.242+0.1193 | | 52.48 |
| *Pleosporales_gen_Incertae_sedis;ASV797* | Int | 0.0002+0.0004 | 0.0086+0.0096 | | 18056.69 |
| *Rhizophydiales_gen_Incertae_sedis;ASV820* | Int | 0.0165+0.0127 | 0+0 | | -10676.42 |
| *Agaricomycetes_gen_Incertae_sedis;ASV824* | Int | 0.0002+0.0004 | 0.0089+0.0049 | | 12984.13 |
| *Spizellomyces;ASV829* | Int | 0.0043+0.0042 | 0.0375+0.0189 | | 3399.71 |
| *Chrysozymaceae_gen_Incertae_sedis;ASV852* | Int | 0.0151+0.0104 | 0+0 | | -11719.16 |
| *Penicillium;ASV864* | Int | 0.3544+0.2189 | 0.0366+0.0209 | | -1316.79 |
| *Panaeolus;ASV879* | Int | 0.0041+0.0022 | 0.4084+0.533 | | 386.33 |
| *Clitocybe;ASV905* | Int | 0.0004+0.0007 | 0.0062+0.0032 | | 28898.39 |
| *Operculomyces;ASV933* | Int | 0.0257+0.035 | 0+0 | | -56413.25 |
| *Entoloma;ASV937* | Int | 0+0 | 0.01+0.0099 | | 13416.97 |
| *Lobulomycetales_gen_Incertae_sedis;ASV939* | Int | 0.0098+0.0031 | 0.177+0.1827 | | 3754.71 |
| *Ascomycota_gen_Incertae_sedis;ASV967* | Int | 0.3384+0.0981 | 0.7707+0.1912 | | 698.1 |
| *Conocybe;ASV983* | Int | 0+0 | 0.0041+0.0031 | | 5689.17 |
| *Piptocephalis;ASV998* | Int | 0.0732+0.0224 | 0.0232+0.0031 | | -3477.95 |
| *Fungi_gen_Incertae_sedis;ASV1010* | Int | 0.0216+0.0088 | 0.0527+0.0166 | | 6185.03 |
| *Spizellomycetales_gen_Incertae_sedis;ASV1024* | Int | 0.0002+0.0004 | 0.0243+0.0213 | | 9402.14 |
| *Coprinellus;ASV1029* | Int | 0.0048+0.0054 | 0.0387+0.0291 | | 764.81 |
| *Ascomycota_gen_Incertae_sedis;ASV1056* | Int | 0.0132+0.0057 | 0.0043+0.0029 | | -531.32 |
| *Rhizophlyctis;ASV1057* | Int | 0+0 | 0.0127+0.0085 | | 9876.23 |
| *Spizellomycetales_gen_Incertae_sedis;ASV1074* | Int | 0.0021+0.0019 | 0.0393+0.0283 | | 35021.74 |
| *Psathyrella;ASV1077* | Int | 0.0004+0.0004 | 0.0074+0.0066 | | 654423.25 |
| *Psathyrella;ASV1082* | Int | 0.1935+0.0901 | 0.0441+0.0349 | | -1689.46 |
| *Conocybe;ASV1090* | Int | 0.003+0.0021 | 0.0498+0.0517 | | 4891.88 |
| *Keithomyces;ASV1098* | Int | 0.0312+0.0166 | 0.2651+0.1658 | | 1337.33 |
| *Serendipita;ASV1126* | Int | 0.0012+0.0014 | 0.0147+0.0153 | | 27153.05 |
| *Powellomyces;ASV1161* | Int | 0.1606+0.1004 | 0.0269+0.0151 | | -1300.81 |
| *Microbotryomycetes_gen_Incertae_sedis;ASV1168* | Int | 0.0049+0.0049 | 0.0509+0.0251 | | 3760.21 |
| *Endogonomycetes_gen_Incertae_sedis;ASV1209* | Int | 0+0 | 0.0025+0.0018 | | 8314.36 |
| *Rhodotorula;ASV1216* | Int | 0.0002+0.0004 | 0.0071+0.0078 | | 9402.3 |
| *Spizellomycetales_gen_Incertae_sedis;ASV1218* | Int | 0.0034+0.0031 | 0+0 | | -11425.91 |
| *Samsoniella;ASV1225* | Int | 0.0053+0.0064 | 0+0 | | -7516.22 |
| *Sistotrema;ASV1230* | Int | 0.019+0.0041 | 0.0677+0.021 | | 1703.36 |
| *Sistotrema;ASV1231* | Int | 0.0236+0.015 | 0.0033+0.0066 | | -387.76 |
| *Funneliformis;ASV1281* | Int | 0.0041+0.0034 | 0.0301+0.0186 | | 254.22 |
| *Beauveria;ASV1295* | Int | 1.1124+0.3987 | 1.9872+0.5103 | | 6.99 |
| *Basidiomycota_gen_Incertae_sedis;ASV1314* | Int | 0.1605+0.107 | 0.0075+0.0108 | | -1403.16 |
| *Murispora;ASV1317* | Int | 0.0068+0.0019 | 0.2826+0.3606 | | 1136.45 |
| *Monosporascus;ASV1345* | Int | 0.0112+0.0093 | 0.6305+0.4317 | | 253.86 |
| *Hypocreales_gen_Incertae_sedis;ASV1352* | Int | 0.0287+0.0119 | 0.0821+0.0197 | | 3341.78 |
| *Rhizophydiaceae_gen_Incertae_sedis;ASV1356* | Int | 0.004+0.0023 | 0.009+0.002 | | 1211.68 |
| *Leucosporidium;ASV1364* | Int | 0+0 | 0.0045+0.0044 | | 11213.27 |
| *Microbotryales_gen_Incertae_sedis;ASV1366* | Int | 0.0818+0.0279 | 0.0074+0.0073 | | -1966.4 |
| *Alternaria;ASV1385* | Int | 0.0005+0.0005 | 0.0091+0.0077 | | 65418.54 |
| *Pezizaceae_gen_Incertae_sedis;ASV1392* | Int | 0.0316+0.0209 | 0.0827+0.0304 | | 18567.66 |
| *Aphanoascus;ASV1396* | Int | 0+0 | 0.0049+0.0058 | | 8695.43 |
| *Ascobolus;ASV1413* | Int | 0.001+0.0014 | 0.0172+0.0132 | | 13751.06 |
| *Furcasterigmium;ASV1446* | Int | 0.0248+0.0129 | 0.1558+0.0445 | | 831.47 |
| *Pseudogymnoascus;ASV1450* | Int | 0.0421+0.0228 | 0.0143+0.0072 | | -301.18 |
| *Trichoderma;ASV1455* | Int | 0.0011+0.0008 | 0.0326+0.0468 | | 7492.49 |
| *Trichoderma;ASV1457* | Int | 0.0038+0.0058 | 0.0859+0.0415 | | 1371.44 |
| *Naviculispora;ASV1459* | Int | 0.0359+0.0202 | 0.4741+0.042 | | 126.23 |
| *Mucor;ASV1465* | Int | 0.0185+0.0078 | 0.0048+0.0021 | | -22337.93 |
| *Trichoderma;ASV1475* | Int | 0.0162+0.0145 | 0.0016+0.0019 | | -64388.17 |
| *Talaromyces;ASV1496* | Int | 0.6631+0.2673 | 4.0582+2.9378 | | 115.95 |
| *Chaetomium;ASV1506* | Int | 0.0029+0.0021 | 0.0071+0.0019 | | 1667.11 |
| *Chaetomium;ASV1507* | Int | 0.0006+0.0004 | 0.0518+0.0085 | | 1194.01 |
| *Chaetomium;ASV1509* | Int | 0.4265+0.197 | 0.9637+0.128 | | 217.78 |
| *Preussia;ASV1510* | Int | 0+0 | 0.0066+0.0088 | | 11327.29 |
| *Pseudogymnoascus;ASV1530* | Int | 40.2276+19.892 | 8.5075+3.7431 | | -3.67 |
| *Trichoderma;ASV1538* | Int | 0.2544+0.1154 | 2.4793+1.4997 | | 57.52 |
| *Trichoderma;ASV1540* | Int | 0.1917+0.0931 | 0.5079+0.3775 | | 33.1 |
| *Verticillium;ASV1547* | Int | 0.0021+0.002 | 0.0399+0.0293 | | 14108.2 |
| *Trichoderma;ASV1555* | Int | 0.0006+0.0008 | 0.004+0.0008 | | 32914.09 |
| *Mrakia;ASV1559* | Int | 0.0076+0.0075 | 0.0376+0.0127 | | 9736.24 |
| *Clonostachys;ASV1563* | Int | 0.2468+0.0906 | 1.4774+0.3562 | | 69.39 |
| *Plectosphaerella;ASV1564* | Int | 0.0182+0.0153 | 0.0534+0.0168 | | 14788.17 |
| *Plectosphaerella;ASV1565* | Int | 0.0267+0.0116 | 0.2107+0.1056 | | 1000.87 |
| *Apiotrichum;ASV1573* | Int | 0.004+0.008 | 0.0358+0.0218 | | 28019.41 |
| *Myxotrichum;ASV1583* | Int | 0.0092+0.0099 | 0+0 | | -4787.22 |
| *Talaromyces;ASV1597* | Int | 0.0595+0.0734 | 0.001+0.002 | | -4055.93 |
| *Botryotrichum;ASV7* | Ext | 0.1173+0.0335 | 0.0445+0.035 | | -49594.04 |
| *Chaetomium;ASV10* | Ext | 0.0153+0.0102 | 0.002+0.0008 | | -10963.76 |
| *Chaetomiaceae_gen_Incertae_sedis;ASV13* | Ext | 0.0093+0.0072 | 0.0406+0.011 | | 4138.83 |
| *Sporobolomyces;ASV15* | Ext | 0.5416+0.2353 | 0.1391+0.0531 | | -300.94 |
| *Penicillium;ASV21* | Ext | 0+0 | 0.0052+0.0015 | | 15933.18 |
| *Fusariella;ASV30* | Ext | 0.0039+0.0037 | 0.045+0.049 | | 42847.04 |
| *Acremonium;ASV33* | Ext | 0.5776+0.0184 | 0.1953+0.0401 | | -149.65 |
| *Septoglomus;ASV43* | Ext | 0+0 | 0.0158+0.016 | | 40920.16 |
| *Podila;ASV50* | Ext | 6.3058+3.4394 | 2.0442+1.0707 | | -50.08 |
| *Halosphaeriaceae_gen_Incertae_sedis;ASV52* | Ext | 0.0361+0.0339 | 0.0005+0.0011 | | -8171.05 |
| *Heterocephalacria;ASV62* | Ext | 0.0002+0.0005 | 0.0132+0.0042 | | 6478.4 |
| *Rozellomycota_gen_Incertae_sedis;ASV72* | Ext | 0.0155+0.0098 | 0.0047+0.0036 | | -723.83 |
| *Rozellomycota_gen_Incertae_sedis;ASV73* | Ext | 0.0031+0.0028 | 0.0003+0.0005 | | -3602.12 |
| *Glomus;ASV74* | Ext | 0.0006+0.0004 | 0.0432+0.0417 | | 5895.67 |
| *Linnemannia;ASV85* | Ext | 0.94+0.5515 | 0.0729+0.0376 | | -160.19 |
| *Pyrenochaeta;ASV99* | Ext | 0.0635+0.0288 | 0.0202+0.015 | | -168.78 |
| *Cyathus;ASV104* | Ext | 0.0019+0.0018 | 0.0118+0.0093 | | 121178.41 |
| *Betamyces;ASV117* | Ext | 0.0081+0.0045 | 0.0313+0.0083 | | 5899.05 |
| *Betamyces;ASV120* | Ext | 0+0 | 0.0122+0.0078 | | 11317.41 |
| *Betamyces;ASV122* | Ext | 0.0002+0.0005 | 0.0095+0.0086 | | 409729.86 |
| *Rhizophydiales_gen_Incertae_sedis;ASV123* | Ext | 0.0099+0.0041 | 0.0015+0.003 | | -1295.52 |
| *Paraglomus;ASV125* | Ext | 0.0948+0.037 | 0.0037+0.005 | | -890.57 |
| *Paraglomus;ASV127* | Ext | 0.0077+0.0056 | 0.0008+0.0016 | | -1322.89 |
| *Lecythophora;ASV137* | Ext | 0.0057+0.0053 | 0.0215+0.0058 | | 10794.96 |
| *Sclerostagonospora;ASV147* | Ext | 0.0066+0.0086 | 0.638+0.5131 | | 245.06 |
| *Sclerostagonospora;ASV148* | Ext | 0.0256+0.0074 | 1.6415+0.346 | | 40.61 |
| *Filobasidium;ASV153* | Ext | 0.0096+0.006 | 0+0 | | -14939.95 |
| *Glomeraceae_gen_Incertae_sedis;ASV157* | Ext | 0+0 | 0.0055+0.0041 | | 4002.69 |
| *Trichophaeopsis;ASV186* | Ext | 0.0002+0.0004 | 0.1012+0.1908 | | 31286.87 |
| *Sporormiella;ASV194* | Ext | 0.007+0.0038 | 0.1368+0.0577 | | 626.84 |
| *Sporormiaceae_gen_Incertae_sedis;ASV197* | Ext | 0.1465+0.1012 | 0.8263+0.4171 | | 177.4 |
| *Claroideoglomus;ASV204* | Ext | 0.0002+0.0005 | 0.0097+0.0039 | | 8688.55 |
| *Xenasmatella;ASV206* | Ext | 0.0002+0.0004 | 0.0161+0.0153 | | 776.39 |
| *Aaosphaeria;ASV207* | Ext | 0.0062+0.0078 | 0.3601+0.1632 | | 241.06 |
| *Rozellomycota_gen_Incertae_sedis;ASV208* | Ext | 0+0 | 0.0342+0.0198 | | 5800.45 |
| *Microascales_gen_Incertae_sedis;ASV229* | Ext | 0.0113+0.0037 | 0.0039+0.0037 | | -611.18 |
| *Ascomycota_gen_Incertae_sedis;ASV232* | Ext | 0.1795+0.0465 | 0.0651+0.042 | | -1892.28 |
| *Sordariales_gen_Incertae_sedis;ASV239* | Ext | 0.0011+0.0013 | 0.0291+0.0125 | | 5557.9 |
| *Gibellulopsis;ASV245* | Ext | 0.2576+0.1153 | 0.0799+0.0265 | | -1188.7 |
| *Brunneochlamydosporium;ASV247* | Ext | 0.0432+0.02 | 0.01+0.0076 | | -337.45 |
| *Lasiosphaeriaceae_gen_Incertae_sedis;ASV267* | Ext | 0.0516+0.0412 | 0.0051+0.0029 | | -405.71 |
| *Ascomycota_gen_Incertae_sedis;ASV275* | Ext | 2.4726+0.7698 | 0.8989+0.4842 | | -142.73 |
| *Coniochaeta;ASV279* | Ext | 0.0291+0.0092 | 0.5191+0.5049 | | 461.27 |
| *Kurtzmanomyces;ASV287* | Ext | 0+0 | 0.0061+0.0054 | | 3799.12 |
| *Periconia;ASV292* | Ext | 0.012+0.0041 | 0.173+0.0904 | | 659.02 |
| *Fusidium;ASV306* | Ext | 0.0639+0.0097 | 0.0124+0.0122 | | -1710.51 |
| *Ascomycota_gen_Incertae_sedis;ASV312* | Ext | 0.2589+0.1753 | 0.0677+0.0256 | | -2210.32 |
| *Rhizophagus;ASV319* | Ext | 0+0 | 0.0033+0.0014 | | 23038.51 |
| *Thelebolaceae_gen_Incertae_sedis;ASV327* | Ext | 0.2602+0.0642 | 0.0561+0.0179 | | -392.81 |
| *Pseudeurotiaceae_gen_Incertae_sedis;ASV330* | Ext | 0.1436+0.0349 | 0.0192+0.0134 | | -792.91 |
| *Pleosporales_gen_Incertae_sedis;ASV332* | Ext | 0.017+0.0132 | 0.002+0.0022 | | -12189.16 |
| *Articulospora;ASV349* | Ext | 0.0032+0.0039 | 0.0389+0.0528 | | 1019.22 |
| *Trichoderma;ASV354* | Ext | 0.3617+0.2838 | 4.1657+1.4406 | | 32.02 |
| *Trichoderma;ASV357* | Ext | 0.0022+0.0018 | 0.0496+0.0552 | | 6799.97 |
| *Lasiosphaeriaceae_gen_Incertae_sedis;ASV363* | Ext | 0.3018+0.1217 | 0.0706+0.0269 | | -778.37 |
| *Sordariales_gen_Incertae_sedis;ASV365* | Ext | 0.0877+0.1179 | 0.0021+0.0035 | | -627.95 |
| *Sordariales_gen_Incertae_sedis;ASV380* | Ext | 0.037+0.0149 | 0.0003+0.0005 | | -3188.33 |
| *Schizothecium;ASV382* | Ext | 0.222+0.0338 | 0.0701+0.0634 | | -1450.46 |
| *Schizothecium;ASV386* | Ext | 0.0705+0.0448 | 0.029+0.0082 | | -5881.65 |
| *Schizothecium;ASV388* | Ext | 0.0006+0.0004 | 0.0113+0.0073 | | 13996.26 |
| *Sordariales_gen_Incertae_sedis;ASV390* | Ext | 0.1643+0.1421 | 0.0081+0.0088 | | -1605.67 |
| *Neoschizothecium;ASV392* | Ext | 0.1513+0.1227 | 0.4483+0.1092 | | 37.78 |
| *Sordariales_gen_Incertae_sedis;ASV400* | Ext | 0.0726+0.0158 | 1.6719+0.6115 | | 46.92 |
| *Sordariales_gen_Incertae_sedis;ASV402* | Ext | 0.0004+0.0008 | 0.0123+0.0053 | | 8381.12 |
| *Nectriaceae_gen_Incertae_sedis;ASV404* | Ext | 0.0891+0.0402 | 0.4636+0.1187 | | 230.39 |
| *Hypocreales_gen_Incertae_sedis;ASV407* | Ext | 0.0166+0.0058 | 0.0065+0.0054 | | -466.46 |
| *Fusarium;ASV409* | Ext | 0.1371+0.0498 | 0.0266+0.0123 | | -985.86 |
| *Hypocreales_gen_Incertae_sedis;ASV412* | Ext | 0.2223+0.1161 | 0.064+0.0452 | | -30.32 |
| *Hypocreales_gen_Incertae_sedis;ASV413* | Ext | 0.0002+0.0004 | 0.0075+0.0046 | | 187797.09 |
| *Dactylonectria;ASV422* | Ext | 0.2729+0.1014 | 0.0438+0.0361 | | -419.46 |
| *Glomus;ASV433* | Ext | 0+0 | 0.0342+0.0358 | | 38880.06 |
| *Glomeraceae_gen_Incertae_sedis;ASV435* | Ext | 0.0002+0.0004 | 0.0331+0.0258 | | 4239.55 |
| *Magnaporthaceae_gen_Incertae_sedis;ASV450* | Ext | 0.0107+0.0112 | 0.0744+0.0261 | | 5308.41 |
| *Chalara;ASV451* | Ext | 0.0118+0.006 | 0.0484+0.0148 | | 4978.59 |
| *Myrmecridium;ASV462* | Ext | 0+0 | 0.0036+0.0031 | | 9618.94 |
| *Ascomycota_gen_Incertae_sedis;ASV477* | Ext | 0.1327+0.0297 | 0.0445+0.0224 | | -1018.04 |
| *Pleotrichocladium;ASV487* | Ext | 0.1095+0.0407 | 0.0218+0.0177 | | -1632.48 |
| *Papiliotrema;ASV496* | Ext | 0.0069+0.0067 | 0+0 | | -6286.92 |
| *Pleosporales_gen_Incertae_sedis;ASV503* | Ext | 0+0 | 0.0128+0.0071 | | 11351.19 |
| *Pyrenochaetopsis;ASV507* | Ext | 0.0157+0.0029 | 0.1592+0.0448 | | 472.45 |
| *Phialophora;ASV508* | Ext | 0+0 | 0.0147+0.0146 | | 43373.95 |
| *Lipomyces;ASV512* | Ext | 0.005+0.0034 | 0+0 | | -3220.34 |
| *Kockovaella;ASV513* | Ext | 0.0002+0.0005 | 0.0515+0.0463 | | 186.56 |
| *Ramophialophora;ASV529* | Ext | 0.2338+0.0751 | 0.0159+0.0292 | | -502.83 |
| *Hypocreales_gen_Incertae_sedis;ASV532* | Ext | 0+0 | 0.0161+0.0052 | | 5598.29 |
| *Kiflimonium;ASV533* | Ext | 0.1926+0.158 | 0.0446+0.0191 | | -98.18 |
| *Hypocreales_gen_Incertae_sedis;ASV536* | Ext | 0.0914+0.1671 | 0.0008+0.0015 | | -490.84 |
| *Ceratobasidiaceae_gen_Incertae_sedis;ASV537* | Ext | 0.0004+0.0005 | 0.0513+0.0216 | | 1572.8 |
| *Pyronemataceae_gen_Incertae_sedis;ASV545* | Ext | 0.0018+0.0018 | 0.0196+0.0248 | | 578.04 |
| *Agaricomycetes_gen_Incertae_sedis;ASV552* | Ext | 0.105+0.0433 | 0.0021+0.0042 | | -841.28 |
| *Ceratobasidiaceae_gen_Incertae_sedis;ASV555* | Ext | 0.0015+0.0005 | 0.0003+0.0005 | | -4314.53 |
| *Cheilymenia;ASV567* | Ext | 0.0181+0.0128 | 0.0008+0.0015 | | -13187.61 |
| *Lasiosphaeriaceae_gen_Incertae_sedis;ASV591* | Ext | 0.0027+0.0015 | 0.039+0.0264 | | 10036.35 |
| *Ascomycota_gen_Incertae_sedis;ASV595* | Ext | 0.0322+0.0158 | 2.5678+2.7716 | | 73.8 |
| *Mortierella;ASV602* | Ext | 2.6141+0.8872 | 1.068+0.3347 | | -173.35 |
| *Mortierella;ASV604* | Ext | 0.1264+0.0797 | 0.0139+0.0108 | | -1001.6 |
| *Mortierella;ASV605* | Ext | 0.1328+0.096 | 0.0214+0.0113 | | -62.8 |
| *Mortierella;ASV606* | Ext | 0.7502+0.5096 | 0.0483+0.0382 | | -186.1 |
| *Hypocreales_gen_Incertae_sedis;ASV608* | Ext | 0.0179+0.0142 | 0.0013+0.0026 | | -420.88 |
| *Ceratobasidium;ASV609* | Ext | 0.0008+0.0011 | 0.0225+0.0066 | | 4434.91 |
| *Ascomycota_gen_Incertae_sedis;ASV619* | Ext | 0+0 | 0.0122+0.0104 | | 14593.47 |
| *Mucor;ASV625* | Ext | 0.1698+0.2543 | 0.0236+0.0097 | | -12419.42 |
| *Spizellomycetales_gen_Incertae_sedis;ASV634* | Ext | 0.0093+0.0091 | 0+0 | | -27248.6 |
| *Lobulomycetales_gen_Incertae_sedis;ASV652* | Ext | 0.0009+0.0008 | 0.0036+0.002 | | 238174.2 |
| *Trichoderma;ASV655* | Ext | 0.0041+0.0053 | 0.0709+0.0349 | | 1812.25 |
| *Olpidiaceae_gen_Incertae_sedis;ASV656* | Ext | 0.0002+0.0004 | 0.1085+0.0715 | | 1518.08 |
| *Rhizophlyctis;ASV661* | Ext | 0.0005+0.0009 | 0.04+0.0645 | | 18469.51 |
| *Pleosporales_gen_Incertae_sedis;ASV665* | Ext | 0.0108+0.0031 | 0.234+0.058 | | 303.06 |
| *Cyphellophora;ASV683* | Ext | 0.0002+0.0005 | 0.0157+0.0094 | | 11080.48 |
| *Acremonium;ASV689* | Ext | 0.6637+0.5777 | 0.0998+0.0346 | | -270.56 |
| *Stachybotrys;ASV692* | Ext | 4.8583+0.6159 | 1.1432+0.1246 | | -17.96 |
| *Albifimbria;ASV696* | Ext | 0.0022+0.0044 | 0.0484+0.0282 | | 2983.33 |
| *Paramyrothecium;ASV699* | Ext | 0+0 | 0.0096+0.0086 | | 3095.02 |
| *Paramicrothyrium;ASV703* | Ext | 0.0068+0.0022 | 0.1507+0.07 | | 1066.87 |
| *Stephanonectria;ASV711* | Ext | 0.0065+0.0098 | 0.0815+0.0426 | | 332.83 |
| *Clonostachys;ASV715* | Ext | 0.0496+0.0368 | 0.0034+0.0034 | | -146.4 |
| *Ascomycota_gen_Incertae_sedis;ASV716* | Ext | 1.0192+0.2692 | 0.1594+0.0465 | | -87.73 |
| *Tetracladium;ASV727* | Ext | 0.0968+0.0295 | 0.171+0.0108 | | 1048.03 |
| *Fusarium;ASV737* | Ext | 0.2422+0.0172 | 0.0712+0.0492 | | -429.1 |
| *Nectriaceae_gen_Incertae_sedis;ASV742* | Ext | 0.1191+0.0435 | 0.0391+0.0033 | | -2070.32 |
| *Cladosporium;ASV744* | Ext | 1.4276+0.3911 | 0.7993+0.2742 | | -14.36 |
| *Preussia;ASV748* | Ext | 0.0518+0.0249 | 0.0181+0.0063 | | -10526.33 |
| *Alternaria;ASV750* | Ext | 0.0393+0.0434 | 0.0064+0.0046 | | -619.19 |
| *Fusarium;ASV768* | Ext | 0.1213+0.0256 | 0.0268+0.0215 | | -1440.76 |
| *Anthracocystis;ASV773* | Ext | 0.2369+0.1817 | 0.0725+0.0325 | | -50.99 |
| *Auricularia;ASV777* | Ext | 0.0017+0.001 | 0.0971+0.1213 | | 1840.48 |
| *Auriculariales_gen_Incertae_sedis;ASV779* | Ext | 0+0 | 0.0033+0.0036 | | 10038.63 |
| *Auricularia;ASV780* | Ext | 0.001+0.0012 | 0.096+0.1069 | | 2350.58 |
| *Neosetophoma;ASV786* | Ext | 0.0405+0.0411 | 1.0236+0.2252 | | 66.38 |
| *Ophiosphaerella;ASV789* | Ext | 0.6931+0.108 | 0.2792+0.1568 | | -323.45 |
| *Pleosporales_gen_Incertae_sedis;ASV790* | Ext | 0.0006+0.0008 | 0.0574+0.0275 | | 2095.58 |
| *Phaeosphaeria;ASV796* | Ext | 0.0068+0.0045 | 0.0198+0.0056 | | 15520.39 |
| *Pleosporales_gen_Incertae_sedis;ASV797* | Ext | 0.0009+0.0012 | 0.0134+0.0096 | | 17811.32 |
| *Pochonia;ASV815* | Ext | 0.0306+0.0117 | 0.0051+0.0053 | | -7646.68 |
| *Metacordyceps;ASV818* | Ext | 0.2092+0.0647 | 0.0972+0.0263 | | -1420.08 |
| *Basidiomycota_gen_Incertae_sedis;ASV825* | Ext | 0.0013+0.001 | 0.0147+0.0065 | | 10908.45 |
| *Spizellomyces;ASV829* | Ext | 0.0087+0.0064 | 0.0371+0.0377 | | 845.38 |
| *Ramicandelaber;ASV835* | Ext | 0.012+0.005 | 0.024+0.0055 | | 54918.47 |
| *Chrysozymaceae_gen_Incertae_sedis;ASV852* | Ext | 0.0124+0.0132 | 0.0003+0.0005 | | -691.12 |
| *Spizellomycetales_gen_Incertae_sedis;ASV855* | Ext | 0+0 | 0.0072+0.0043 | | 11756.82 |
| *Penicillium;ASV864* | Ext | 0.0212+0.0086 | 0.1306+0.0703 | | 1283.1 |
| *Panaeolus;ASV879* | Ext | 0.0043+0.0035 | 0.2821+0.2608 | | 1647.63 |
| *Piptocephalis;ASV885* | Ext | 0.0024+0.0034 | 0.0197+0.0125 | | 15096.7 |
| *Lobulomycetales_gen_Incertae_sedis;ASV888* | Ext | 0+0 | 0.0039+0.0027 | | 4646.17 |
| *Fungi_gen_Incertae_sedis;ASV896* | Ext | 0+0 | 0.0284+0.0359 | | 38743.74 |
| *Spizellomyces;ASV900* | Ext | 0.0002+0.0004 | 0.1137+0.2178 | | 176807.47 |
| *Basidiomycota_gen_Incertae_sedis;ASV918* | Ext | 0.0187+0.0153 | 0+0 | | -5536.69 |
| *Spizellomyces;ASV931* | Ext | 0+0 | 0.0128+0.0121 | | 3968.66 |
| *Entoloma;ASV937* | Ext | 0.0982+0.1108 | 0.0005+0.001 | | -2075.99 |
| *Lobulomycetales_gen_Incertae_sedis;ASV939* | Ext | 0.0146+0.0112 | 0.0358+0.0135 | | 219.03 |
| *Syncephalis;ASV962* | Ext | 0.002+0.001 | 0.0167+0.004 | | 4773.74 |
| *Hypocreales_gen_Incertae_sedis;ASV968* | Ext | 0.8431+0.388 | 0.0094+0.0044 | | -95.19 |
| *Conocybe;ASV980* | Ext | 0+0 | 0.0068+0.005 | | 42856.68 |
| *Piptocephalis;ASV998* | Ext | 0.0471+0.0074 | 0.0281+0.0124 | | -421.26 |
| *Fungi_gen_Incertae_sedis;ASV1001* | Ext | 0.0058+0.0031 | 0.0008+0.001 | | -145751.8 |
| *Fungi_gen_Incertae_sedis;ASV1010* | Ext | 0.0218+0.0065 | 0.0529+0.0166 | | 15334.03 |
| *Coprinellus;ASV1029* | Ext | 0.0033+0.0036 | 0.0217+0.0073 | | 8660.24 |
| *Mortierella;ASV1041* | Ext | 0.0033+0.0022 | 0.0161+0.0082 | | 394.73 |
| *Spizellomycetales_gen_Incertae_sedis;ASV1051* | Ext | 0.0004+0.0005 | 0.0054+0.0041 | | 458312.67 |
| *Spizellomycetales_gen_Incertae_sedis;ASV1054* | Ext | 0+0 | 0.0115+0.0121 | | 2349.86 |
| *Ascomycota_gen_Incertae_sedis;ASV1056* | Ext | 0.0409+0.0219 | 0.0038+0.0045 | | -5965.18 |
| *Pyrenochaeta;ASV1060* | Ext | 0.5734+0.0832 | 0.3043+0.1718 | | -3781.43 |
| *Microascaceae_gen_Incertae_sedis;ASV1070* | Ext | 0.0497+0.0188 | 0.0023+0.0019 | | -2146.42 |
| *Spizellomycetales_gen_Incertae_sedis;ASV1074* | Ext | 0.0004+0.0005 | 0.0276+0.0133 | | 5729.06 |
| *Psathyrella;ASV1079* | Ext | 0.0122+0.0153 | 0+0 | | -5476.4 |
| *Agaricales_gen_Incertae_sedis;ASV1089* | Ext | 0.0002+0.0004 | 0.138+0.1611 | | 123596.12 |
| *Conocybe;ASV1090* | Ext | 0.0037+0.0024 | 0.4537+0.2392 | | 353.49 |
| *Conocybe;ASV1094* | Ext | 0.0019+0.002 | 0.1584+0.2032 | | 9779.8 |
| *Spizellomycetales_gen_Incertae_sedis;ASV1100* | Ext | 0.0004+0.0008 | 0.0562+0.0356 | | 2797.24 |
| *Hyalorbilia;ASV1117* | Ext | 0.0989+0.0434 | 0.0311+0.0083 | | -345.62 |
| *Apiosporaceae_gen_Incertae_sedis;ASV1118* | Ext | 0.0057+0.0028 | 0.021+0.0144 | | 1042.98 |
| *Spiromyces;ASV1120* | Ext | 0.0105+0.0069 | 0.0008+0.001 | | -40330.74 |
| *Spiromastix;ASV1127* | Ext | 0.0068+0.0034 | 0.0005+0.0006 | | -12661.88 |
| *Chrysosporium;ASV1128* | Ext | 0.8427+0.3597 | 0.3273+0.0629 | | -11.1 |
| *Olpidiaster;ASV1136* | Ext | 0.001+0.0015 | 0.0616+0.0761 | | 7973.85 |
| *Penicillium;ASV1149* | Ext | 0.0002+0.0004 | 0.0121+0.009 | | 162776.5 |
| *Penicillium;ASV1150* | Ext | 0.0009+0.0012 | 0.0142+0.0088 | | 7857.09 |
| *Leucoagaricus;ASV1155* | Ext | 0.0751+0.0976 | 0+0 | | -25653.59 |
| *Apodus;ASV1192* | Ext | 0.0004+0.0005 | 0.0088+0.008 | | 166371.21 |
| *Endogonomycetes_gen_Incertae_sedis;ASV1209* | Ext | 0.0055+0.0039 | 0+0 | | -25939.32 |
| *Udeniozyma;ASV1215* | Ext | 0.0794+0.0227 | 0.0085+0.0053 | | -1104.92 |
| *Rhodotorula;ASV1216* | Ext | 0.0002+0.0004 | 0.0356+0.0186 | | 3392.53 |
| *Akenomyces;ASV1242* | Ext | 0.0144+0.0128 | 0+0 | | -13869.18 |
| *Exophiala;ASV1244* | Ext | 0.0332+0.0095 | 0.0698+0.0173 | | 3406.11 |
| *Thysanorea;ASV1247* | Ext | 0.005+0.0034 | 0.0002+0.0005 | | -835.9 |
| *Funneliformis;ASV1279* | Ext | 0+0 | 0.0157+0.0211 | | 15192.06 |
| *Funneliformis;ASV1291* | Ext | 0.0002+0.0005 | 0.0185+0.0143 | | 8369.16 |
| *Mortierella;ASV1304* | Ext | 0.02+0.0187 | 0+0 | | -50051.74 |
| *Ascomycota_gen_Incertae_sedis;ASV1306* | Ext | 0.5635+0.0549 | 0.3642+0.1556 | | -34.12 |
| *Marquandomyces;ASV1309* | Ext | 0.0147+0.0083 | 0.1754+0.0746 | | 622.96 |
| *Marquandomyces;ASV1310* | Ext | 1.5822+0.2105 | 6.1539+2.5426 | | 18.59 |
| *Ascomycota_gen_Incertae_sedis;ASV1311* | Ext | 0.0123+0.0168 | 0.1434+0.0293 | | 726.43 |
| *Murispora;ASV1317* | Ext | 0.0134+0.0044 | 0.2217+0.1429 | | 535.56 |
| *Monosporascus;ASV1345* | Ext | 1.4088+1.8984 | 0.1046+0.0891 | | -1324.22 |
| *Hypocreales_gen_Incertae_sedis;ASV1352* | Ext | 0.1242+0.1018 | 0.0264+0.0187 | | -93151.8 |
| *Ascomycota_gen_Incertae_sedis;ASV1353* | Ext | 0.014+0.0132 | 0.0005+0.0006 | | -15378.35 |
| *Rhizophydiaceae_gen_Incertae_sedis;ASV1356* | Ext | 0.0025+0.0007 | 0.0192+0.0139 | | 14287.33 |
| *Kendrickiella;ASV1361* | Ext | 0.0008+0.0007 | 0.0095+0.0055 | | 99499.02 |
| *Arachnomyces;ASV1363* | Ext | 0.0953+0.067 | 0.005+0.0069 | | -2073.55 |
| *Alternaria;ASV1383* | Ext | 0.2622+0.1062 | 0.0916+0.0589 | | -30.39 |
| *Alternaria;ASV1385* | Ext | 0.0004+0.0005 | 0.0104+0.009 | | 36212.4 |
| *Alternaria;ASV1386* | Ext | 0.0038+0.0025 | 0.0457+0.0193 | | 4334.57 |
| *Mortierella;ASV1388* | Ext | 0.481+0.0835 | 0.1686+0.0689 | | -260.22 |
| *Mortierella;ASV1389* | Ext | 1.0222+0.1834 | 0.4305+0.2151 | | -239.36 |
| *Fungi_gen_Incertae_sedis;ASV1390* | Ext | 2.9155+0.8712 | 0.8946+0.2734 | | -80.7 |
| *Spizellomycetales_gen_Incertae_sedis;ASV1415* | Ext | 0.0096+0.0043 | 0.0005+0.001 | | -13424.94 |
| *Agaricomycetes_gen_Incertae_sedis;ASV1427* | Ext | 0.0091+0.0087 | 0+0 | | -55157.32 |
| *Filobasidium;ASV1440* | Ext | 0.0443+0.0242 | 0.0127+0.0092 | | -7007.57 |
| *Sarocladium;ASV1442* | Ext | 0.0044+0.001 | 0.0008+0.0015 | | -68224.04 |
| *Gaeumannomyces;ASV1443* | Ext | 0.0376+0.0207 | 0.0015+0.0013 | | -4056.35 |
| *Pseudogymnoascus;ASV1450* | Ext | 0.027+0.0096 | 0.0062+0.0031 | | -6534.72 |
| *Trichoderma;ASV1455* | Ext | 0.0002+0.0005 | 0.01+0.0102 | | 266019.54 |
| *Naviculispora;ASV1459* | Ext | 0.0436+0.0373 | 0.4703+0.0565 | | 141.45 |
| *Microascus;ASV1468* | Ext | 0.0094+0.0099 | 0+0 | | -11929.09 |
| *Cyphellophora;ASV1489* | Ext | 0.0002+0.0004 | 0.0341+0.0185 | | 2517.71 |
| *Talaromyces;ASV1496* | Ext | 0.3749+0.2494 | 5.1754+7.129 | | 50.18 |
| *Penicillium;ASV1515* | Ext | 0.6475+0.1784 | 1.8524+0.5374 | | 81.56 |
| *Trichoderma;ASV1533* | Ext | 0.2307+0.1463 | 0.0031+0.003 | | -494.07 |
| *Trichoderma;ASV1537* | Ext | 0.4039+0.2059 | 0.0626+0.013 | | -548.49 |
| *Trichoderma;ASV1538* | Ext | 0.2587+0.1115 | 2.5326+1.3344 | | 46.87 |
| *Trichoderma;ASV1540* | Ext | 0.1347+0.0345 | 0.3479+0.2019 | | 8500.53 |
| *Neoascochyta;ASV1543* | Ext | 0.0064+0.0066 | 0.0005+0.0011 | | -1989.4 |
| *Trichoderma;ASV1555* | Ext | 0.001+0.0015 | 0.0082+0.0036 | | 1637.47 |
| *Cephalotrichum;ASV1557* | Ext | 0.0042+0.0038 | 0+0 | | -49851.3 |
| *Mrakia;ASV1559* | Ext | 0.0058+0.0067 | 0.0276+0.0125 | | 219.93 |
| *Plectosphaerella;ASV1565* | Ext | 0.0389+0.0107 | 0.292+0.0669 | | 262.2 |
| *Fusarium;ASV1568* | Ext | 0.7874+0.3281 | 0.2004+0.0969 | | -221.25 |
| *Apiotrichum;ASV1573* | Ext | 0.006+0.0099 | 0.059+0.0667 | | 142366.65 |
| *Penicillium;ASV1580* | Ext | 0.0547+0.0124 | 0.0986+0.0369 | | 332.82 |
| *Fusicolla;ASV1584* | Ext | 0.824+0.2915 | 0.3993+0.1516 | | -16.14 |
| *Trichocladium;ASV1585* | Ext | 0.2426+0.2263 | 0.0275+0.018 | | -1024.1 |
| *Funneliformis;ASV1588* | Ext | 0.0042+0.0036 | 0.234+0.2287 | | 935.61 |
| *Talaromyces;ASV1593* | Ext | 0.0033+0.0022 | 0+0 | | -4956.91 |
| *Talaromyces;ASV1594* | Ext | 0+0 | 0.0279+0.0086 | | 2403.13 |

**Table S7.** Detailed information on metagenomics sequences annotated to ABi03, RU47, and OMG16 genomes.

| **Microorganisms** | **Classified reads** | **No. of reads mapping to genome (identity %, alignment %)** | | | | **Genome** |
| --- | --- | --- | --- | --- | --- | --- |
|  |  | **100%, 100%** | **100%, 50%** | **97%, 50%** |  |  |
| Bacillus atrophaeus | 30.866 | 25.983 | 26.661 | 30.445 |  | ABi03 |
| Pseudomonas sp. RU47 | 5.201 | 3.958 | 4.244 | 5.008 |  | RU47 |
| Trichoderma harzianum | 689 | 234 | 256 | 356 |  | OMG16 |

**Table S8.** The list of potential plant beneficial functions of rhizosphere microorganisms in a customized database, established including protein sequences downloaded from online Kyoto Encyclopedia of Genes and Genomes (KEGG) Orthology database.

| **Related plant beneficial function** | **Function name in KEGG database** | **Functional Genes included** | **KO** |
| --- | --- | --- | --- |
| Hormone production | Auxin | 10 |  |
|  |  | E4.1.1.74, ipdC; indolepyruvate decarboxylase [EC:4.1.1.74] | K04103 |
|  |  | E3.5.1.4, amiE; amidase [EC:3.5.1.4] | K01426 |
|  |  | TRP1; anthranilate synthase / indole-3-glycerol phosphate synthase / phosphoribosylanthranilate isomerase [EC:4.1.3.27 4.1.1.48 5.3.1.24] | K13501 |
|  |  | TRP3; anthranilate synthase / indole-3-glycerol phosphate synthase [EC:4.1.3.27 4.1.1.48] | K01656 |
|  |  | K06001, trpB; tryptophan synthase beta chain [EC:4.2.1.20] | K06001, K01696 |
|  |  | TRP; tryptophan synthase [EC:4.2.1.20] | K01694 |
|  |  | trpA; tryptophan synthase alpha chain [EC:4.2.1.20] | K01695 |
|  |  | trpD; anthranilate phosphoribosyltransferase [EC:2.4.2.18] | K00766 |
|  |  | tryptophan 2-monooxygenase | K00466 |
|  |  | nitrilase | K01501 |
|  |  |  |  |
| Trehalose production (osmoprotectant) |  | 5 |  |
|  |  | otsA; trehalose 6-phosphate synthase [EC:2.4.1.15] | K00697 |
|  |  | otsB; trehalose 6-phosphate phosphatase [EC:3.1.3.12] | K01087 |
|  |  | treS; trehalose transport system substrate-binding protein | K17311 |
|  |  | treT; trehalose synthase [EC:2.4.1.245] | K13057 |
|  |  | treZ, glgZ; maltooligosyltrehalose trehalohydrolase [EC:3.2.1.141] | K01236 |
| Nutrient cycling | Nitrogen | 50 |  |
|  |  | nirS; nitrite reductase (NO-forming) / hydroxylamine reductase [EC:1.7.2.1 1.7.99.1] | K15864 |
|  |  | E1.13.12.16; nitronate monooxygenase [EC:1.13.12.16] | K00459 |
|  |  | NR; nitrate reductase (NAD(P)H) [EC:1.7.1.1 1.7.1.2 1.7.1.3] | K10534 |
|  |  | nirK; E1.7.2.1; nitrite reductase (NO-forming) [EC:1.7.2.1] | K00368 |
|  |  | E1.7.99.1, hcp; hydroxylamine reductase [EC:1.7.99.1] | K05601 |
|  |  | nasA; E1.7.99.4C; nitrate reductase catalytic subunit [EC:1.7.99.4] | K00372 |
|  |  | E4.2.1.1; carbonic anhydrase [EC:4.2.1.1] | K01672, K18245, K18246 |
|  |  | NIAD; nitrate reductase (NADPH) [EC:1.7.1.3] | K10534 |
|  |  | amoA; ammonia monooxygenase subunit A [EC:1.14.99.39] | K10944 |
|  |  | amoB; ammonia monooxygenase subunit B | K10945 |
|  |  | amoC; ammonia monooxygenase subunit C | K10946 |
|  |  | anfG; nitrogenase [EC:1.18.6.1] | K00531 |
|  |  | cah; carbonic anhydrase [EC:4.2.1.1] | K01674 |
|  |  | cynT, can; carbonic anhydrase [EC:4.2.1.1] | K01673 |
|  |  | hao; hydroxylamine oxidase [EC:1.7.3.4] | not found |
|  |  | napA; periplasmic nitrate reductase NapA [EC:1.7.99.4 -> other EC number: EC:1.9.6.1] | K02567 |
|  |  | napB; nitrate reductase (cytochrome), electron transfer subunit | K02568 |
|  |  | napC; cytochrome c-type protein NapC | K02569 |
|  |  | napD; periplasmic nitrate reductase NapD | K02570 |
|  |  | napE; periplasmic nitrate reductase NapE | K02571 |
|  |  | napF; ferredoxin-type protein NapF | K02572 |
|  |  | napG; ferredoxin-type protein NapG | K02573 |
|  |  | napH; ferredoxin-type protein NapH | K02574 |
|  |  | narB; ferredoxin-nitrate reductase [EC:1.7.7.2] | K00367 |
|  |  | narI; narV; nitrate reductase 2, gamma subunit [EC:1.7.99.4] | K00374 |
|  |  | narJ; narW; nitrate reductase 2, delta subunit | K00373 |
|  |  | narH; narY; nitrate reductase 2, beta subunit [EC:1.7.99.4] | K00371 |
|  |  | narG; narZ; nitrate reductase 2, alpha subunit [EC:1.7.99.4] | K00370 |
|  |  | nifB; nitrogen fixation protein NifB | K02585 |
|  |  | nifD; nitrogenase molybdenum-iron protein alpha chain [EC:1.18.6.1] | K02586 |
|  |  | nifH; nitrogenase iron protein NifH [EC:1.18.6.1] | K02588 |
|  |  | nifHD2, nifI2; nitrogen regulatory protein PII 2 | K02590 |
|  |  | nifK; nitrogenase molybdenum-iron protein beta chain [EC:1.18.6.1] | K02591 |
|  |  | nifN; nitrogenase molybdenum-iron protein NifN | K02592 |
|  |  | nifT; nitrogen fixation protein NifT | K02593 |
|  |  | nifV; homocitrate synthase NifV | K02594 |
|  |  | nifW; nitrogenase-stabilizing/protective protein | K02595 |
|  |  | nirA; ferredoxin-nitrite reductase [EC:1.7.7.1] | K00366 |
|  |  | nirB; nitrite reductase (NAD(P)H) large subunit [EC:1.7.1.4] | K00362 |
|  |  | nirD; nitrite reductase (NAD(P)H) small subunit [EC:1.7.1.4] | K00363 |
|  |  | norB; nitric oxide reductase subunit B [EC:1.7.2.5] | K04561 |
|  |  | norC; nitric oxide reductase subunit C | K02305 |
|  |  | norD; nitric oxide reductase NorD protein [EC:1.7.99.7] | K02448 |
|  |  | norF; nitric oxide reductase NorF protein [EC:1.7.99.7] | K04747 |
|  |  | nosZ; nitrous-oxide reductase [EC:1.7.2.4] | K00376 |
|  |  | nrfB; cytochrome c-type protein NrfB | K04013 |
|  |  | nrfC; protein NrfC | K04014 |
|  |  | nrfD; formate-dependent nitrate reductase complex, transmembrane protein | K04015 |
|  |  | nrfE; formate-dependent nitrite reductase, possible assembly protein | K04016 |
|  |  | nrfF; formate-dependent nitrite reductase complex NrfF subunit | K04017 |
|  | Phosphate | 9 |  |
|  | Pyrroloquinoline quinone cofactor biosynthesis | pqqA; pyrroloquinoline quinone biosynthesis protein A | K06135 |
|  |  | pqqB; pyrroloquinoline quinone biosynthesis protein B | K06136 |
|  |  | pqqC; pyrroloquinoline-quinone synthase [EC:1.3.3.11] | K06137 |
|  |  | pqqD; pyrroloquinoline quinone biosynthesis protein D | K06138 |
|  |  | pqqE; PqqA peptide cyclase [EC:1.21.98.4] | K06139 |
|  |  | gdh; glucose 1-dehydrogenase [EC:1.1.1.47] | K00034, K22969 |
|  |  | phoD; alkaline phosphatase D [EC:3.1.3.1] | K01113 |
|  |  | phoN; acid phosphatase (class A) [EC:3.1.3.2] | K09474 |
| Biocontrol/antibiotic production | Siderophores | 22 |  |
|  | dhbF; nonribosomal peptide synthetase DhbF | dhbF; glyine---[glycyl-carrier protein] ligase [EC:6.2.1.66] | K04780 |
|  |  | entA; 2,3-dihydro-2,3-dihydroxybenzoate dehydrogenase [EC:1.3.1.28] | K00216 |
|  |  | entB, dhbB, vibB, mxcF; bifunctional isochorismate lyase / aryl carrier protein [EC:3.3.2.1] | K01252 |
|  | dhbE; 2,3-dihydroxybenzoate-AMP ligase [EC:2.7.7.58] | entE, dhbE, vibE, mxcE; 2,3-dihydroxybenzoate-AMP ligase [EC:2.7.7.58] | K02363 |
|  | entF; enterobactin synthetase component F [EC:2.7.7.-] | entF; L-serine---[L-seryl-carrier protein] ligase [EC:6.3.2.14 6.2.1.72] | K02364 |
|  |  | irp1, HMWP1; yersiniabactin nonribosomal peptide/polyketide synthase | K04786 |
|  |  | irp2, HMWP2; yersiniabactin nonribosomal peptide synthetase | K04784 |
|  |  | irp3, ybtU; yersiniabactin synthetase, thiazolinyl reductase component | K04785 |
|  |  | irp5, ybtE; yersiniabactin salicyl-AMP ligase [EC:6.3.2.-] | K04783 |
|  |  | mbtA; mycobactin salicyl-AMP ligase [EC:6.3.2.-] | K04787 |
|  |  | mbtB; mycobactin phenyloxazoline synthetase | K04788 |
|  |  | mbtC; mycobactin polyketide synthetase MbtC | K04790 |
|  |  | mbtD; mycobactin polyketide synthetase MbtD | K04791 |
|  |  | mbtE; mycobactin peptide synthetase MbtE | K04789 |
|  |  | mbtF; mycobactin peptide synthetase MbtF | K04792 |
|  |  | mbtG; mycobactin lysine-N-oxygenase | K04793 |
|  |  | mbtI, irp9, ybtS; salicylate synthetase [EC:5.4.4.2 4.2.99.21] | K04781 |
|  |  | pchB; isochorismate pyruvate lyase [EC:4.2.99.21] | K04782 |
|  |  | pchD; pyochelin biosynthesis protein PchD | K12238 |
|  |  | pchE; dihydroaeruginoic acid synthetase | K12239 |
|  | pchF; pyochelin synthetase | pchF; L-cysteine---[L-cysteinyl-carrier protein] ligase PchF [EC:6.2.1.69] | K12240 |
|  |  | vibF; nonribosomal peptide synthetase VibF | K12237 |
|  | secondary metabolites | 45 |  |
|  | Surfactin | srfAA | K15654 |
|  |  | srfAB | K15655 |
|  |  | srfAC | K15656 |
|  |  | srfAD | K15657 |
|  | pyrrolnitrin production | prnD; aminopyrrolnitrin oxygenase [EC:1.14.13.-] | K19982 |
|  | pyoluteorin production | pltR, pltK, pltJ, pltI | K22711, K25187, K25188, K25189 |
|  | phenazine (PCA) production | phzA_B | K20260 |
|  | hydrogen cyanide production | hcnA; hydrogen cyanide synthase HcnA [EC:1.4.99.5] | K10814 |
|  |  | hcnB; hydrogen cyanide synthase HcnB [EC:1.4.99.5] | K10815 |
|  |  | hcnC; hydrogen cyanide synthase HcnC [EC:1.4.99.5] | K10816 |
|  | isopenicillin N synthase | cefD; isopenicillin-N epimerase [EC:5.1.1.17] | K04127 |
|  |  | E1.21.3.1; PCBC; isopenicillin-N synthase [EC:1.21.3.1] | K04126 |
|  | rhizoxin | rhiG; rhizoxin biosynthesis acyltransferase | K15469 |
|  |  | rhiA; rhizoxin biosynthesis, polyketide synthase / nonribosomal peptide synthetase RhiA | K15674 |
|  |  | rhiB; rhizoxin biosynthesis, polyketide synthase / nonribosomal peptide synthetase RhiB | K15675 |
|  |  | rhiC; rhizoxin biosynthesis, polyketide synthase RhiC | K15676 |
|  |  | rhiD; rhizoxin biosynthesis, polyketide synthase RhiD | K15677 |
|  |  | rhiE; rhizoxin biosynthesis, polyketide synthase RhiE | K15678 |
|  |  | rhiF; rhizoxin biosynthesis, polyketide synthase RhiF | K15679 |
|  | Colicin | cvpA; membrane protein required for colicin V production | K03558 |
|  | fengycin | fenD | K15665 |
|  | iturin | ituA, mycA, bmyA; iturin family lipopeptide synthetase A | K15661 |
|  |  | ituB, mycB, bmyB; iturin family lipopeptide synthetase B | K15662 |
|  |  | ituC, mycC, bmyC; iturin family lipopeptide synthetase C | K15663 |
|  | cyclic lipopeptide: arthrofactin | ofaA, arfA; arthrofactin-type cyclic lipopeptide synthetase A | K15658 |
|  |  | ofaB, arfB; arthrofactin-type cyclic lipopeptide synthetase B | K15659 |
|  |  | ofaC, arfC; arthrofactin-type cyclic lipopeptide synthetase C | K15660 |
|  | glucanase | E3.2.1.6; endo-1,3(4)-beta-glucanase [EC:3.2.1.6] | K01180 |
|  |  | E3.2.1.4; endoglucanase [EC:3.2.1.4] | K01179 |
|  |  | XEG; xyloglucan-specific endo-beta-1,4-glucanase [EC:3.2.1.151] | K18576 |
|  |  | CEL74A; xyloglucan-specific exo-beta-1,4-glucanase [EC:3.2.1.155] | K18578 |
|  |  | bcsZ; endoglucanase [EC:3.2.1.4] | K20542 |
|  | fungal cellulase | CELB; cellulase [EC:3.2.1.4] | K19357 |
|  | cellulase | CBH1; cellulose 1,4-beta-cellobiosidase [EC:3.2.1.91] | K01225 |
|  |  | CBH2, cbhA; cellulose 1,4-beta-cellobiosidase [EC:3.2.1.91] | K19668 |
|  |  | E3.2.1.21; beta-glucosidase [EC:3.2.1.21] | K01188 |
|  |  | bglX; beta-glucosidase [EC:3.2.1.21] | K05349 |
|  |  | bglB; beta-glucosidase [EC:3.2.1.21] | K05350 |
|  |  | E3.2.1.86A, celF; 6-phospho-beta-glucosidase [EC:3.2.1.86] | K01222 |
|  |  | E3.2.1.86B, bglA; 6-phospho-beta-glucosidase [EC:3.2.1.86] | K01223 |
|  |  | E2.4.1.20; cellobiose phosphorylase [EC:2.4.1.20] | K00702 |
|  | chitinase | chiA; bifunctional chitinase/lysozyme [EC:3.2.1.14 3.2.1.17] | K13381 |
| Antibiotic degradation |  | 1 |  |
|  |  | phlG; 2,4-diacetylphloroglucinol hydrolase [EC:3.7.1.24] | K23519 |
| Stress protection |  | 6 |  |
|  | ACC deaminase (acdS) | 1 |  |
|  |  | E3.5.99.7; 1-aminocyclopropane-1-carboxylate deaminase [EC:3.5.99.7] | K01505 |
|  | Spermidine | 5 |  |
|  |  | E2.5.1.16, SRM, speE; spermidine synthase [EC:2.5.1.16] | K00797 |
|  |  | potA; spermidine/putrescine transport system ATP-binding protein [EC:3.6.3.31] | K11072 |
|  |  | potB; spermidine/putrescine transport system permease protein | K11071 |
|  |  | potC; spermidine/putrescine transport system permease protein | K11070 |
|  |  | potD; spermidine/putrescine transport system substrate-binding protein | K11069 |
| Degradation of aromatic compounds |  | 105 |  |
|  |  | acd = glutaryl-CoA dehydrogenase | K16173 |
|  |  | GCDH, gcdH; glutaryl-CoA dehydrogenase [EC:1.3.8.6] | K00252 |
|  |  | EPHX1; microsomal epoxide hydrolase [EC:3.3.2.9] | K01253 |
|  | Chlorocyclohexane and chlorobenzene degradation [PATH:ko00361] | E1.13.11.37; hydroxyquinol 1,2-dioxygenase [EC:1.13.11.37] | K04098 |
|  |  | pcpB; E1.14.13.50; pentachlorophenol monooxygenase [EC:1.14.13.50] | K03391 |
|  |  | E1.14.13.7; phenol 2-monooxygenase [EC:1.14.13.7] | K03380 |
|  |  | E1.3.1.32; maleylacetate reductase [EC:1.3.1.32] | K00217 |
|  |  | E3.1.1.45; carboxymethylenebutenolidase [EC:3.1.1.45] | K01061 |
|  |  | E3.8.1.2; 2-haloacid dehalogenase [EC:3.8.1.2] | K01560 |
|  |  | dehH, E3.8.1.3; haloacetate dehalogenase [EC:3.8.1.3] | K01561 |
|  |  | E5.5.1.7; chloromuconate cycloisomerase [EC:5.5.1.7] | K01860 |
|  |  | bedC1, todC1; benzene/toluene dioxygenase subunit alpha [EC:1.14.12.3 1.14.12.11] | K03268 |
|  |  | bphC; biphenyl-2,3-diol 1,2-dioxygenase [EC:1.13.11.39] | K00462 |
|  |  | catA; catechol 1,2-dioxygenase [EC:1.13.11.1] | K03381 |
|  |  | catB; muconate cycloisomerase [EC:5.5.1.1] | K01856 |
|  |  | dmpB; catechol 2,3-dioxygenase [EC:1.13.11.2] | K00446 |
|  |  | tfdB; 2,4-dichlorophenol 6-monooxygenase [EC:1.14.13.20] | K10676 |
|  | Benzoate degradation [PATH:ko00362] | CMLE; carboxy-cis,cis-muconate cyclase [EC:5.5.1.5] | K14334 |
|  |  | DHBD; 2,3-dihydroxybenzoate decarboxylase [EC:4.1.1.46] | K14333 |
|  |  | E1.14.13.12; benzoate 4-monooxygenase [EC:1.14.13.12] | K07824 |
|  |  | E1.3.1.62; pimeloyl-CoA dehydrogenase [EC:1.3.1.62] | K04118 |
|  |  | E3.7.1.9; 2-hydroxymuconate-semialdehyde hydrolase [EC:3.7.1.9] | K10216 |
|  |  | E4.1.1.77; 4-oxalocrotonate decarboxylase [EC:4.1.1.77] | K01617 |
|  |  | E5.3.2.-; 4-oxalocrotonate tautomerase [EC:5.3.2.-] | K01821 |
|  |  | aliA; cyclohexanecarboxylate-CoA ligase [EC:6.2.1.-] | K04116 |
|  |  | aliB; cyclohexanecarboxyl-CoA dehydrogenase [EC:1.3.99.-] | K04117 |
|  |  | badA; benzoate-CoA ligase [EC:6.2.1.25] | K04110 |
|  |  | badD; benzoyl-CoA reductase subunit [EC:1.3.7.8] | K04112 |
|  | bcrB_badE = benzoyl-CoA reductase subunit | badE; benzoyl-CoA reductase subunit [EC:1.3.7.8] | K04113 |
|  | bcrA_badF = benzoyl-CoA reductase subunit | badF; benzoyl-CoA reductase subunit [EC:1.3.7.8] | K04114 |
|  |  | badG; benzoyl-CoA reductase subunit [EC:1.3.7.8] | K04115 |
|  |  | badH; 2-hydroxycyclohexanecarboxyl-CoA dehydrogenase [EC:1.1.1.-] | K07535 |
|  |  | badI; 2-ketocyclohexanecarboxyl-CoA hydrolase [EC:3.1.2.-] | K07536 |
|  |  | badK; cyclohex-1-ene-1-carboxyl-CoA hydratase [EC:4.2.1.-] | K07534 |
|  |  | benB-xylY; benzoate/toluate 1,2-dioxygenase subunit beta [EC:1.14.12.10 1.14.12.-] | K05550 |
|  |  | benC-xylZ; benzoate/toluate 1,2-dioxygenase electron transfer component | K05784 |
|  |  | benD-xylL; dihydroxycyclohexadiene carboxylate dehydrogenase [EC:1.3.1.25 1.3.1.-] | K05783 |
|  |  | catC; muconolactone D-isomerase [EC:5.3.3.4] | K03464 |
|  |  | cmtC, dhbA; 2,3-dihydroxy-p-cumate/2,3-dihydroxybenzoate 3,4-dioxygenase [EC:1.13.11.- 1.13.11.14] | K10621 |
|  |  | cmtD, dhbB; HCOMODA/2-hydroxy-3-carboxy-muconic semialdehyde decarboxylase [EC:4.1.1.-] | K10622 |
|  |  | dch; cyclohexa-1,5-dienecarbonyl-CoA hydratase [EC:4.2.1.100] | K07537 |
|  |  | had; 6-hydroxycyclohex-1-ene-1-carboxyl-CoA dehydrogenase [EC:1.1.1.-] | K07538 |
|  |  | hbaA; 4-hydroxybenzoate-CoA ligase [EC:6.2.1.27 6.2.1.25] | K04105 |
|  |  | hbaB, hcrC; 4-hydroxybenzoyl-CoA reductase subunit gamma [EC:1.3.7.9] | K04107 |
|  | hcrA_hbaC = 4-hydroxybenzoyl-CoA reductase subunit alpha | hbaC, hcrA; 4-hydroxybenzoyl-CoA reductase subunit alpha [EC:1.3.7.9] | K04108 |
|  |  | hbaD, hcrB; 4-hydroxybenzoyl-CoA reductase subunit beta [EC:1.3.7.9] | K04109 |
|  |  | ligA; protocatechuate 4,5-dioxygenase, alpha chain [EC:1.13.11.8] | K04100 |
|  | ligB = protocatechuate 4,5-dioxygenase beta chain | ligB; protocatechuate 4,5-dioxygenase, beta chain [EC:1.13.11.8] | K04101 |
|  |  | ligI; 2-pyrone-4,6-dicarboxylate lactonase [EC:3.1.1.57] | K10221 |
|  |  | ligJ; 4-oxalmesaconate hydratase [EC:4.2.1.83] | K10220 |
|  | oah = 6-oxo-cyclohex-1-ene-carbonyl-CoA hydrolase | oah; 6-oxo-cyclohex-1-ene-carbonyl-CoA hydrolase [EC:3.7.1.-] | K07539 |
|  |  | pcaB; 3-carboxy-cis,cis-muconate cycloisomerase [EC:5.5.1.2] | K01857 |
|  |  | pcaC; 4-carboxymuconolactone decarboxylase [EC:4.1.1.44] | K01607 |
|  |  | pcaD; 3-oxoadipate enol-lactonase [EC:3.1.1.24] | K01055 |
|  |  | pcaF; 3-oxoadipyl-CoA thiolase [EC:2.3.1.174] | K07823 |
|  |  | pcaG; protocatechuate 3,4-dioxygenase, alpha subunit [EC:1.13.11.3] | K00448 |
|  |  | pcaH; protocatechuate 3,4-dioxygenase, beta subunit [EC:1.13.11.3] | K00449 |
|  |  | pcaI; 3-oxoadipate CoA-transferase, alpha subunit [EC:2.8.3.6] | K01031 |
|  |  | pcaJ; 3-oxoadipate CoA-transferase, beta subunit [EC:2.8.3.6] | K01032 |
|  |  | pobA; p-hydroxybenzoate 3-monooxygenase [EC:1.14.13.2] | K00481 |
|  | 00363 Bisphenol degradation [PATH:ko00363] | E1.13.11.41; 2,4'-dihydroxyacetophenone dioxygenase [EC:1.13.11.41] | K05913 |
|  | 00621 Dioxin degradation [PATH:ko00621] | E1.14.13.1; salicylate hydroxylase [EC:1.14.13.1] | K00480 |
|  |  | bphA; biphenyl 2,3-dioxygenase subunit alpha [EC:1.14.12.18] | K08689 |
|  |  | bphB; cis-2,3-dihydrobiphenyl-2,3-diol dehydrogenase [EC:1.3.1.56] | K08690 |
|  | 00622 Xylene degradation [PATH:ko00622] | cmtAb; p-cumate 2,3-dioxygenase subunit alpha [EC:1.14.12.-] | K10619 |
|  |  | cmtB; 2,3-dihydroxy-2,3-dihydro-p-cumate dehydrogenase [EC:1.3.1.58] | K10620 |
|  |  | cmtE; HOMODA hydrolase [EC:3.7.1.-] | K10623 |
|  |  | cymAa; p-cymene monooxygenase [EC:1.14.13.-] | K10616 |
|  |  | cymB; p-cumic alcohol dehydrogenase | K10617 |
|  |  | cymC; p-cumic aldehyde dehydrogenase | K10618 |
|  |  | etbD, todF; 2-hydroxy-6-oxohepta-2,4-dienoate hydroxylase [EC:3.7.1.-] | K18092 |
|  |  | xylC; benzaldehyde dehydrogenase (NAD) [EC:1.2.1.28] | K00141 |
|  | 00623 Toluene degradation [PATH:ko00623] | E1.17.99.1; 4-cresol dehydrogenase (hydroxylating) [EC:1.17.99.1] | K05797 |
|  |  | bbsB; benzoylsuccinyl-CoA thiolase BbsB subunit [EC:2.3.1.-] | K07550 |
|  |  | bbsC; 2-[hydroxy(phenyl)methyl]-succinyl-CoA dehydrogenase BbsC subunit [EC:1.1.1.35] | K07547 |
|  |  | bbsD; 2-[hydroxy(phenyl)methyl]-succinyl-CoA dehydrogenase BbsD subunit [EC:1.1.1.35] | K07548 |
|  |  | bbsE; benzylsuccinate CoA-transferase BbsE subunit [EC:2.8.3.15] | K07543 |
|  |  | bbsF; benzylsuccinate CoA-transferase BbsF subunit [EC:2.8.3.15] | K07544 |
|  |  | bbsG; (R)-benzylsuccinyl-CoA dehydrogenase [EC:1.3.8.3] | K07545 |
|  |  | bbsH; E-phenylitaconyl-CoA hydratase [EC:4.2.1.-] | K07546 |
|  | 00624 Polycyclic aromatic hydrocarbon degradation [PATH:ko00624] | E1.14.12.7; phthalate 4,5-dioxygenase [EC:1.14.12.7] | K18068 |
|  |  | E4.1.1.55; 4,5-dihydroxyphthalate decarboxylase [EC:4.1.1.55] | K04102 |
|  |  | nidA; PAH dioxygenase large subunit [EC:1.13.11.-] | K11943 |
|  |  | nidB; PAH dioxygenase small subunit [EC:1.13.11.-] | K11944 |
|  |  | nidD; aldehyde dehydrogenase [EC:1.2.1.-] | K11947 |
|  |  | phdF; extradiol dioxygenase [EC:1.13.11.-] | K11945 |
|  |  | phdG; hydratase-aldolase [EC:4.1.2.-] | K11946 |
|  |  | phdI; 1-hydroxy-2-naphthoate dioxygenase [EC:1.13.11.38] | K11948 |
|  |  | phdJ; 4-(2-carboxyphenyl)-2-oxobut-3-enoate aldolase [EC:4.1.2.34] | K11949 |
|  | 00627 Aminobenzoate degradation [PATH:ko00627] | E3.1.3.41; 4-nitrophenyl phosphatase [EC:3.1.3.41] | K01101 |
|  | ACMR = anthraniloyl-CoA monooxygenase | E1.14.13.40; anthraniloyl-CoA monooxygenase [EC:1.14.13.40] | K09461 |
|  | bsdC; 4-hydroxybenzoate decarboxylase subunit C [EC:4.1.1.61] | E4.1.1.61; 4-hydroxybenzoate decarboxylase [EC:4.1.1.61] | K16239 |
|  |  | E5.1.2.2; mandelate racemase [EC:5.1.2.2] | K01781 |
|  |  | abmG; 2-aminobenzoate-CoA ligase [EC:6.2.1.32] | K08295 |
|  |  | antA; anthranilate 1,2-dioxygenase (deaminating, decarboxylating) large subunit [EC:1.14.12.1] | K05599 |
|  |  | antB; anthranilate 1,2-dioxygenase (deaminating, decarboxylating) small subunit [EC:1.14.12.1] | K05600 |
|  |  | antC; anthranilate dioxygenase reductase | K11311 |
|  |  | desB, galA; gallate dioxygenase [EC:1.13.11.57] | K04099 |
|  |  | mdlC; benzoylformate decarboxylase [EC:4.1.1.7] | K01576 |
|  |  | vanA; vanillate monooxygenase [EC:1.14.13.82] | K03862 |
|  |  | vanB; vanillate monooxygenase [EC:1.14.13.82] | K03863 |
|  |  | hyaB_hybC = hydrogenase large subunit | K06281 |
|  |  | hyaA_hybO = hydrogenase small subunit | K06282 |
|  |  | hapE 4-hydroxyacetophenone monooxygenase | K14520 |
|  |  | desA = syringate O-demethylase | K15064 |
| Chemotaxis-related genes |  | 60 |  |
|  |  | aer; aerotaxis receptor | K03776 |
|  |  | cheA; two-component system, chemotaxis family, sensor kinase CheA [EC:2.7.13.3] | K03407 |
|  |  | cheB; two-component system, chemotaxis family, response regulator CheB [EC:3.1.1.61] | K03412 |
|  |  | cheBR; two-component system, chemotaxis family, CheB/CheR fusion protein [EC:2.1.1.80 3.1.1.61] | K13924 |
|  |  | cheC; chemotaxis protein CheC | K03410 |
|  |  | cheD; chemotaxis protein CheD [EC:3.5.1.44] | K03411 |
|  |  | cheR; chemotaxis protein methyltransferase CheR [EC:2.1.1.80] | K00575 |
|  |  | cheV; two-component system, chemotaxis family, response regulator CheV | K03415 |
|  |  | cheW; purine-binding chemotaxis protein CheW | K03408 |
|  |  | cheX; chemotaxis protein CheX | K03409 |
|  | CheY | cheY; two-component system, chemotaxis family, response regulator CheY | K03413 |
|  |  | cheZ; chemotaxis protein CheZ | K03414 |
|  |  | dppA; dipeptide transport system substrate-binding protein | K12368 |
|  |  | fliG; flagellar motor switch protein FliG | K02410 |
|  |  | fliM; flagellar motor switch protein FliM | K02416 |
|  |  | fliNY, fliN; flagellar motor switch protein FliN/FliY | K02417 |
|  |  | malE; maltose/maltodextrin transport system substrate-binding protein | K10108 |
|  |  | mcp; methyl-accepting chemotaxis protein | K03406 |
|  |  | mglB; methyl-galactoside transport system substrate-binding protein | K10540 |
|  |  | motA; chemotaxis protein MotA | K02556 |
|  |  | motB; chemotaxis protein MotB | K02557 |
|  |  | rbsB; ribose transport system substrate-binding protein | K10439 |
|  |  | tap; methyl-accepting chemotaxis protein IV, peptide sensor receptor | K05877 |
|  |  | tar; methyl-accepting chemotaxis protein II, aspartate sensor receptor | K05875 |
|  |  | trg; methyl-accepting chemotaxis protein III, ribose and galactose sensor receptor | K05876 |
|  |  | tsr; methyl-accepting chemotaxis protein I, serine sensor receptor | K05874 |
|  | Flagellar assembly | flgA; flagella basal body P-ring formation protein FlgA | K02386 |
|  |  | flgB; flagellar basal-body rod protein FlgB | K02387 |
|  |  | flgC; flagellar basal-body rod protein FlgC | K02388 |
|  |  | flgD; flagellar basal-body rod modification protein FlgD | K02389 |
|  |  | flgE; flagellar hook protein FlgE | K02390 |
|  |  | flgF; flagellar basal-body rod protein FlgF | K02391 |
|  |  | flgG; flagellar basal-body rod protein FlgG | K02392 |
|  |  | flgH; flagellar L-ring protein precursor FlgH | K02393 |
|  |  | flgI; flagellar P-ring protein precursor FlgI | K02394 |
|  |  | flgK; flagellar hook-associated protein 1 FlgK | K02396 |
|  |  | flgL; flagellar hook-associated protein 3 FlgL | K02397 |
|  |  | flgM; negative regulator of flagellin synthesis FlgM | K02398 |
|  |  | flgN; flagella synthesis protein FlgN | K02399 |
|  |  | flhA; flagellar biosynthesis protein FlhA | K02400 |
|  |  | flhB; flagellar biosynthetic protein FlhB | K02401 |
|  |  | flhC; flagellar transcriptional activator FlhC | K02402 |
|  |  | flhD; flagellar transcriptional activator FlhD | K02403 |
|  |  | fliC; flagellin | K02406 |
|  |  | fliD; flagellar hook-associated protein 2 | K02407 |
|  |  | fliE; flagellar hook-basal body complex protein FliE | K02408 |
|  |  | fliF; flagellar M-ring protein FliF | K02409 |
|  |  | fliH; flagellar assembly protein FliH | K02411 |
|  |  | fliI; flagellum-specific ATP synthase [EC:3.6.3.14] | K02412 |
|  |  | fliJ; flagellar FliJ protein | K02413 |
|  |  | fliK; flagellar hook-length control protein FliK | K02414 |
|  |  | fliOZ, fliO; flagellar protein FliO/FliZ | K02418 |
|  |  | fliP; flagellar biosynthetic protein FliP | K02419 |
|  |  | fliQ; flagellar biosynthetic protein FliQ | K02420 |
|  |  | fliR-flhB; flagellar biosynthetic protein FliR/FlhB | K13820 |
|  |  | fliR; flagellar biosynthetic protein FliR | K02421 |
|  |  | fliS; flagellar protein FliS | K02422 |
|  |  | fliT; flagellar protein FliT | K02423 |
|  |  | motC; chemotaxis protein MotC | K10564 |
|  |  | motD; chemotaxis protein MotD | K10565 |
| C4- dicarboxilates (sensing and transport) |  | 4 |  |
|  |  | dctR; two-component system, LuxR family, response regulator DctR | K11712 |
|  |  | dctS; two-component system, LuxR family, sensor histidine kinase DctS [EC:2.7.13.3] | K11711 |
|  |  | dctA; aerobic C4-dicarboxylate transport protein | K11103 |
|  |  | dctB; two-component system, NtrC family, C4-dicarboxylate transport sensor histidine kinase DctB [EC:2.7.13.3] | K10125 |
| Biofilm/EPS and LPS production |  | 16 |  |
|  | polysaccharide export outer membrane protein Wza | wza, gfcE; polysaccharide biosynthesis/export protein | K01991 |
|  | alginate export outer membrane protein AlgE | algE; alginate production protein | K16081 |
|  | alginate biosynthesis acetyltransferase AlgJ | algJ; alginate O-acetyltransferase complex protein AlgJ | K19295 |
|  | colanic acid biosynthesis acetyltransferase WcaB | wcaB; putative colanic acid biosynthesis acetyltransferase WcaB [EC:2.3.1.-] | K03819 |
|  | colanic acid biosynthesis acetyltransferase WcaF | wcaF; putative colanic acid biosynthesis acetyltransferase WcaF [EC:2.3.1.-] | K03818 |
|  | colanic acid/amylovoran biosynthesis pyruvyl transferase WcaK/AmsJ | wcaK, amsJ; colanic acid/amylovoran biosynthesis protein WcaK/AmsJ | K16710 |
|  | capsular polysaccharide export system permease KpsE | kpsE; capsular polysaccharide transport system permease protein | K10107 |
|  | exopolysaccharide biosynthesis transmembrane protein EpsG | epsG; transmembrane protein EpsG | K19419 |
|  | exopolysaccharide biosynthesis tyrosine kinase modulator EpsA | epsA, capA; protein tyrosine kinase modulator | K19420 |
|  | levansucrase SacB | sacB; levansucrase [EC:2.4.1.10] | K00692 |
|  | lipopolysaccharide transport system ATP-binding protein Wzt | wzt, rbfB; lipopolysaccharide transport system ATP-binding protein | K09691 |
|  | LptBFGC lipopolysaccharide export complex permease LptF | lptB; lipopolysaccharide export system ATP-binding protein [EC:7.5.2.5] | K06861 |
|  | LptBFGC lipopolysaccharide export complex permease LptG | lptF; lipopolysaccharide export system permease protein | K07091 |
|  | LptBFGC lipopolysaccharide export complex inner membrane protein LptC | lptG; lipopolysaccharide export system permease protein | K11720 |
|  | GacA, GacS two component regulatory system (small RNA signalling) | uvrY, gacA, varA; two-component system, NarL family, invasion response regulator UvrY | K07689 |
|  |  | barA, gacS, varS; two-component system, NarL family, sensor histidine kinase BarA [EC:2.7.13.3] | K07678 |
| AHL production |  | 1 |  |
|  |  | luxI; acyl homoserine lactone synthase [EC:2.3.1.184] | K22954 |
| Quorum sensing signalling |  | 24 |  |
|  |  | rpfC; two-component system, sensor histidine kinase RpfC [EC:2.7.13.3] | K10715 |
|  |  | rpfF; DSF synthase | K13816 |
|  |  | rpfG; two-component system, response regulator RpfG | K13815 |
|  |  | luxO; two-component system, repressor protein LuxO | K10912 |
|  |  | luxQ; two-component system, autoinducer 2 sensor kinase/phosphatase LuxQ [EC:2.7.13.3 3.1.3.-] | K10909 |
|  |  | luxS; S-ribosylhomocysteine lyase [EC:4.4.1.21] | K07173 |
|  |  | CQSA; CAI-1 autoinducer synthase [EC:2.3.-.-] | K10915 |
|  |  | lasR; LuxR family transcriptional regulator, quorum-sensing system regulator LasR | K18304 |
|  |  | lasI; acyl homoserine lactone synthase [EC:2.3.1.184] | K13060 |
|  |  | rhlR, phzR; LuxR family transcriptional regulator, quorum-sensing system regulator RhlR | K18099 |
|  |  | rhlI, phzI, solI, cepI, tofI; acyl homoserine lactone synthase [EC:2.3.1.184] | K13061 |
|  |  | sdiA; LuxR family transcriptional regulator, quorum-sensing system regulator SdiA | K07782 |
|  |  | agrA, blpR, fsrA; two-component system, LytTR family, response regulator AgrA | K07707 |
|  |  | agrC, blpH, fsrC; two-component system, LytTR family, sensor histidine kinase AgrC [EC:2.7.13.3] | K07706 |
|  |  | lsrA, ego; AI-2 transport system ATP-binding protein | K10558 |
|  |  | lsrB; AI-2 transport system substrate-binding protein | K10555 |
|  |  | lsrC; AI-2 transport system permease protein | K10556 |
|  |  | lsrD; AI-2 transport system permease protein | K10557 |
|  |  | rbsB; ribose transport system substrate-binding protein | K10439 |
|  |  | tdh; threonine 3-dehydrogenase [EC:1.1.1.103] | K00060, K15789 |
|  |  | qseB; two-component system, OmpR family, response regulator QseB | K07666 |
|  |  | qseC; two-component system, OmpR family, sensor histidine kinase QseC [EC:2.7.13.3] | K07645 |
|  |  | tnaA; tryptophanase [EC:4.1.99.1] | K01667 |
| mVOC production |  | 5 |  |
|  |  | E2.2.1.6L, ilvB, ilvG, ilvI; acetolactate synthase I/II/III large subunit [EC:2.2.1.6] | K01652 |
|  |  | E2.2.1.6S, ilvH, ilvN; acetolactate synthase I/III small subunit [EC:2.2.1.6] | K01653 |
|  |  | ilvM; acetolactate synthase II small subunit [EC:2.2.1.6] | K11258 |
|  | 2,3-butanediol (BD) synthesis (budC/ydjL+ilvBN) | budC; meso-butanediol dehydrogenase / (S,S)-butanediol dehydrogenase / diacetyl reductase [EC:1.1.1.- 1.1.1.76 1.1.1.304] | K18009, K03366 |
| Type 3 Secretion systems |  | 11 |  |
|  |  | yscC, sctC, ssaC; type III secretion protein C | K03219 |
|  |  | yscJ, sctJ, hrcJ, ssaJ; type III secretion protein J | K03222 |
|  |  | yscL, sctL; type III secretion protein L | K03223 |
|  |  | yscN, sctN, hrcN, ssaN; ATP synthase in type III secretion protein N [EC:7.4.2.8] | K03224 |
|  |  | yscQ, sctQ, hrcQ, ssaQ, spaO; type III secretion protein Q | K03225 |
|  |  | yscR, sctR, hrcR, ssaR; type III secretion protein R | K03226 |
|  |  | yscS, sctS, hrcS, ssaS; type III secretion protein S | K03227 |
|  |  | yscT, sctT, hrcT, ssaT; type III secretion protein T | K03228 |
|  |  | yscU, sctU, hrcU, ssaU; type III secretion protein U | K03229 |
|  |  | yscV, sctV, hrcV, ssaV, invA; type III secretion protein V | K03230 |
|  |  | yscW, sctW; type III secretion protein W | K04058 |

**Table S9.** Variables of the two main modules (Large Module 1 and 2) of the integrated network (Pearson correlation, |*ρ|*>0.8, *p*<0.05, Benjamini-Hochberg correction). B_tax: Bacterial taxa, F_tax: Fungal taxa, B_genes: Bacterial genes (Metagenome), P_genes: Plant genes (Expression levels), M: Module.

| **Variable** |  | **Group** | **M** |
| --- | --- | --- | --- |
| Chaetomiaceae_gen_Incertae_sedis;ASV13 |  | F_tax | 1 |
| Chaetomiaceae_gen_Incertae_sedis;ASV13 |  | F_tax | 1 |
| Hirsutella;ASV24 |  | F_tax | 1 |
| Rozellomycota_gen_Incertae_sedis;ASV72 |  | F_tax | 1 |
| Rozellomycota_gen_Incertae_sedis;ASV72 |  | F_tax | 1 |
| Glomus;ASV74 |  | F_tax | 1 |
| Glomus;ASV74 |  | F_tax | 1 |
| Glomus;ASV75 |  | F_tax | 1 |
| Pezizales_gen_Incertae_sedis;ASV102 |  | F_tax | 1 |
| Betamyces;ASV120 |  | F_tax | 1 |
| Betamyces;ASV120 |  | F_tax | 1 |
| Lecythophora;ASV137 |  | F_tax | 1 |
| Lecythophora;ASV137 |  | F_tax | 1 |
| Coniochaeta;ASV142 |  | F_tax | 1 |
| Sclerostagonospora;ASV148 |  | F_tax | 1 |
| Sclerostagonospora;ASV148 |  | F_tax | 1 |
| Onygenales_gen_Incertae_sedis;ASV177 |  | F_tax | 1 |
| Sporormiella;ASV194 |  | F_tax | 1 |
| Sporormiella;ASV194 |  | F_tax | 1 |
| Sporormiaceae_gen_Incertae_sedis;ASV197 |  | F_tax | 1 |
| Sporormiaceae_gen_Incertae_sedis;ASV197 |  | F_tax | 1 |
| Aaosphaeria;ASV207 |  | F_tax | 1 |
| Aaosphaeria;ASV207 |  | F_tax | 1 |
| Rozellomycota_gen_Incertae_sedis;ASV208 |  | F_tax | 1 |
| Rozellomycota_gen_Incertae_sedis;ASV208 |  | F_tax | 1 |
| Gibellulopsis;ASV245 |  | F_tax | 1 |
| Gibellulopsis;ASV245 |  | F_tax | 1 |
| Plenodomus;ASV263 |  | F_tax | 1 |
| Ascomycota_gen_Incertae_sedis;ASV291 |  | F_tax | 1 |
| Periconia;ASV292 |  | F_tax | 1 |
| Periconia;ASV292 |  | F_tax | 1 |
| Lobulomycetales_gen_Incertae_sedis;ASV310 |  | F_tax | 1 |
| Articulospora;ASV349 |  | F_tax | 1 |
| Articulospora;ASV349 |  | F_tax | 1 |
| Trichoderma;ASV354 |  | F_tax | 1 |
| Trichoderma;ASV354 |  | F_tax | 1 |
| Neoschizothecium;ASV392 |  | F_tax | 1 |
| Neoschizothecium;ASV392 |  | F_tax | 1 |
| Sordariales_gen_Incertae_sedis;ASV400 |  | F_tax | 1 |
| Sordariales_gen_Incertae_sedis;ASV400 |  | F_tax | 1 |
| Hypocreales_gen_Incertae_sedis;ASV411 |  | F_tax | 1 |
| Hypocreales_gen_Incertae_sedis;ASV413 |  | F_tax | 1 |
| Hypocreales_gen_Incertae_sedis;ASV413 |  | F_tax | 1 |
| Valsonectria;ASV417 |  | F_tax | 1 |
| Sordariales_gen_Incertae_sedis;ASV426 |  | F_tax | 1 |
| Glomeraceae_gen_Incertae_sedis;ASV435 |  | F_tax | 1 |
| Glomeraceae_gen_Incertae_sedis;ASV435 |  | F_tax | 1 |
| Hypholoma;ASV459 |  | F_tax | 1 |
| Hypocreales_gen_Incertae_sedis;ASV479 |  | F_tax | 1 |
| Pyrenochaetopsis;ASV507 |  | F_tax | 1 |
| Pyrenochaetopsis;ASV507 |  | F_tax | 1 |
| Pleosporales_gen_Incertae_sedis;ASV515 |  | F_tax | 1 |
| Hypocreales_gen_Incertae_sedis;ASV532 |  | F_tax | 1 |
| Hypocreales_gen_Incertae_sedis;ASV532 |  | F_tax | 1 |
| Ceratobasidiaceae_gen_Incertae_sedis;ASV537 |  | F_tax | 1 |
| Ceratobasidiaceae_gen_Incertae_sedis;ASV537 |  | F_tax | 1 |
| Actinomucor;ASV539 |  | F_tax | 1 |
| Actinomucor;ASV540 |  | F_tax | 1 |
| Typhula;ASV550 |  | F_tax | 1 |
| Ascomycota_gen_Incertae_sedis;ASV595 |  | F_tax | 1 |
| Ascomycota_gen_Incertae_sedis;ASV595 |  | F_tax | 1 |
| Magnaporthales_gen_Incertae_sedis;ASV596 |  | F_tax | 1 |
| Ceratobasidium;ASV609 |  | F_tax | 1 |
| Ceratobasidium;ASV609 |  | F_tax | 1 |
| Absidia;ASV641 |  | F_tax | 1 |
| Rhizophlyctis;ASV661 |  | F_tax | 1 |
| Rhizophlyctis;ASV661 |  | F_tax | 1 |
| Tausonia;ASV682 |  | F_tax | 1 |
| Albifimbria;ASV696 |  | F_tax | 1 |
| Albifimbria;ASV696 |  | F_tax | 1 |
| Paramicrothyrium;ASV703 |  | F_tax | 1 |
| Paramicrothyrium;ASV703 |  | F_tax | 1 |
| Tetracladium;ASV727 |  | F_tax | 1 |
| Tetracladium;ASV727 |  | F_tax | 1 |
| Lophiotrema;ASV749 |  | F_tax | 1 |
| Neosetophoma;ASV786 |  | F_tax | 1 |
| Neosetophoma;ASV786 |  | F_tax | 1 |
| Pleosporales_gen_Incertae_sedis;ASV797 |  | F_tax | 1 |
| Pleosporales_gen_Incertae_sedis;ASV797 |  | F_tax | 1 |
| Spizellomyces;ASV829 |  | F_tax | 1 |
| Spizellomyces;ASV829 |  | F_tax | 1 |
| Panaeolus;ASV879 |  | F_tax | 1 |
| Panaeolus;ASV879 |  | F_tax | 1 |
| Clitocybe;ASV905 |  | F_tax | 1 |
| Lobulomycetales_gen_Incertae_sedis;ASV939 |  | F_tax | 1 |
| Lobulomycetales_gen_Incertae_sedis;ASV939 |  | F_tax | 1 |
| Ascomycota_gen_Incertae_sedis;ASV967 |  | F_tax | 1 |
| Conocybe;ASV983 |  | F_tax | 1 |
| Fungi_gen_Incertae_sedis;ASV1010 |  | F_tax | 1 |
| Fungi_gen_Incertae_sedis;ASV1010 |  | F_tax | 1 |
| Spizellomycetales_gen_Incertae_sedis;ASV1024 |  | F_tax | 1 |
| Coprinellus;ASV1029 |  | F_tax | 1 |
| Coprinellus;ASV1029 |  | F_tax | 1 |
| Rhizophlyctis;ASV1057 |  | F_tax | 1 |
| Spizellomycetales_gen_Incertae_sedis;ASV1074 |  | F_tax | 1 |
| Spizellomycetales_gen_Incertae_sedis;ASV1074 |  | F_tax | 1 |
| Psathyrella;ASV1077 |  | F_tax | 1 |
| Conocybe;ASV1090 |  | F_tax | 1 |
| Conocybe;ASV1090 |  | F_tax | 1 |
| Keithomyces;ASV1098 |  | F_tax | 1 |
| Serendipita;ASV1126 |  | F_tax | 1 |
| Microbotryomycetes_gen_Incertae_sedis;ASV1168 |  | F_tax | 1 |
| Rhodotorula;ASV1216 |  | F_tax | 1 |
| Rhodotorula;ASV1216 |  | F_tax | 1 |
| Sistotrema;ASV1230 |  | F_tax | 1 |
| Sistotrema;ASV1231 |  | F_tax | 1 |
| Funneliformis;ASV1281 |  | F_tax | 1 |
| Beauveria;ASV1295 |  | F_tax | 1 |
| Murispora;ASV1317 |  | F_tax | 1 |
| Murispora;ASV1317 |  | F_tax | 1 |
| Rhizophydiaceae_gen_Incertae_sedis;ASV1356 |  | F_tax | 1 |
| Rhizophydiaceae_gen_Incertae_sedis;ASV1356 |  | F_tax | 1 |
| Alternaria;ASV1385 |  | F_tax | 1 |
| Alternaria;ASV1385 |  | F_tax | 1 |
| Pezizaceae_gen_Incertae_sedis;ASV1392 |  | F_tax | 1 |
| Aphanoascus;ASV1396 |  | F_tax | 1 |
| Ascobolus;ASV1413 |  | F_tax | 1 |
| Furcasterigmium;ASV1446 |  | F_tax | 1 |
| Trichoderma;ASV1455 |  | F_tax | 1 |
| Trichoderma;ASV1455 |  | F_tax | 1 |
| Naviculispora;ASV1459 |  | F_tax | 1 |
| Naviculispora;ASV1459 |  | F_tax | 1 |
| Trichoderma;ASV1475 |  | F_tax | 1 |
| Trichoderma;ASV1538 |  | F_tax | 1 |
| Trichoderma;ASV1538 |  | F_tax | 1 |
| Trichoderma;ASV1540 |  | F_tax | 1 |
| Trichoderma;ASV1540 |  | F_tax | 1 |
| Verticillium;ASV1547 |  | F_tax | 1 |
| Trichoderma;ASV1555 |  | F_tax | 1 |
| Trichoderma;ASV1555 |  | F_tax | 1 |
| Mrakia;ASV1559 |  | F_tax | 1 |
| Mrakia;ASV1559 |  | F_tax | 1 |
| Clonostachys;ASV1563 |  | F_tax | 1 |
| Plectosphaerella;ASV1565 |  | F_tax | 1 |
| Plectosphaerella;ASV1565 |  | F_tax | 1 |
| Apiotrichum;ASV1573 |  | F_tax | 1 |
| Apiotrichum;ASV1573 |  | F_tax | 1 |
| Fusariella;ASV30 |  | F_tax | 1 |
| Septoglomus;ASV43 |  | F_tax | 1 |
| Heterocephalacria;ASV62 |  | F_tax | 1 |
| Cyathus;ASV104 |  | F_tax | 1 |
| Betamyces;ASV122 |  | F_tax | 1 |
| Sclerostagonospora;ASV147 |  | F_tax | 1 |
| Glomeraceae_gen_Incertae_sedis;ASV157 |  | F_tax | 1 |
| Trichophaeopsis;ASV186 |  | F_tax | 1 |
| Claroideoglomus;ASV204 |  | F_tax | 1 |
| Xenasmatella;ASV206 |  | F_tax | 1 |
| Sordariales_gen_Incertae_sedis;ASV239 |  | F_tax | 1 |
| Coniochaeta;ASV279 |  | F_tax | 1 |
| Kurtzmanomyces;ASV287 |  | F_tax | 1 |
| Rhizophagus;ASV319 |  | F_tax | 1 |
| Trichoderma;ASV357 |  | F_tax | 1 |
| Schizothecium;ASV388 |  | F_tax | 1 |
| Sordariales_gen_Incertae_sedis;ASV402 |  | F_tax | 1 |
| Glomus;ASV433 |  | F_tax | 1 |
| Magnaporthaceae_gen_Incertae_sedis;ASV450 |  | F_tax | 1 |
| Chalara;ASV451 |  | F_tax | 1 |
| Myrmecridium;ASV462 |  | F_tax | 1 |
| Pleosporales_gen_Incertae_sedis;ASV503 |  | F_tax | 1 |
| Phialophora;ASV508 |  | F_tax | 1 |
| Kockovaella;ASV513 |  | F_tax | 1 |
| Pyronemataceae_gen_Incertae_sedis;ASV545 |  | F_tax | 1 |
| Lasiosphaeriaceae_gen_Incertae_sedis;ASV591 |  | F_tax | 1 |
| Ascomycota_gen_Incertae_sedis;ASV619 |  | F_tax | 1 |
| Lobulomycetales_gen_Incertae_sedis;ASV652 |  | F_tax | 1 |
| Trichoderma;ASV655 |  | F_tax | 1 |
| Olpidiaceae_gen_Incertae_sedis;ASV656 |  | F_tax | 1 |
| Pleosporales_gen_Incertae_sedis;ASV665 |  | F_tax | 1 |
| Cyphellophora;ASV683 |  | F_tax | 1 |
| Auricularia;ASV777 |  | F_tax | 1 |
| Auriculariales_gen_Incertae_sedis;ASV779 |  | F_tax | 1 |
| Auricularia;ASV780 |  | F_tax | 1 |
| Pleosporales_gen_Incertae_sedis;ASV790 |  | F_tax | 1 |
| Phaeosphaeria;ASV796 |  | F_tax | 1 |
| Basidiomycota_gen_Incertae_sedis;ASV825 |  | F_tax | 1 |
| Ramicandelaber;ASV835 |  | F_tax | 1 |
| Spizellomycetales_gen_Incertae_sedis;ASV855 |  | F_tax | 1 |
| Lobulomycetales_gen_Incertae_sedis;ASV888 |  | F_tax | 1 |
| Fungi_gen_Incertae_sedis;ASV896 |  | F_tax | 1 |
| Spizellomyces;ASV900 |  | F_tax | 1 |
| Spizellomyces;ASV931 |  | F_tax | 1 |
| Syncephalis;ASV962 |  | F_tax | 1 |
| Mortierella;ASV1041 |  | F_tax | 1 |
| Spizellomycetales_gen_Incertae_sedis;ASV1051 |  | F_tax | 1 |
| Spizellomycetales_gen_Incertae_sedis;ASV1054 |  | F_tax | 1 |
| Agaricales_gen_Incertae_sedis;ASV1089 |  | F_tax | 1 |
| Conocybe;ASV1094 |  | F_tax | 1 |
| Spizellomycetales_gen_Incertae_sedis;ASV1100 |  | F_tax | 1 |
| Apiosporaceae_gen_Incertae_sedis;ASV1118 |  | F_tax | 1 |
| Chrysosporium;ASV1128 |  | F_tax | 1 |
| Olpidiaster;ASV1136 |  | F_tax | 1 |
| Apodus;ASV1192 |  | F_tax | 1 |
| Funneliformis;ASV1279 |  | F_tax | 1 |
| Funneliformis;ASV1291 |  | F_tax | 1 |
| Marquandomyces;ASV1310 |  | F_tax | 1 |
| Kendrickiella;ASV1361 |  | F_tax | 1 |
| Microascus;ASV1468 |  | F_tax | 1 |
| Cyphellophora;ASV1489 |  | F_tax | 1 |
| Penicillium;ASV1515 |  | F_tax | 1 |
| Funneliformis;ASV1588 |  | F_tax | 1 |
| Talaromyces;ASV1594 |  | F_tax | 1 |
| Acidobacteriota_Blastocatellia_Pyrinomonadales_Pyrinomonadaceae_RB41_ASV2846 |  | B_tax | 1 |
| Acidobacteriota_Vicinamibacteria_Vicinamibacterales_Vicinamibacteraceae_Unclassified_ASV783 |  | B_tax | 1 |
| Acidobacteriota_Vicinamibacteria_Vicinamibacterales_Vicinamibacteraceae_Unclassified_ASV902 |  | B_tax | 1 |
| Actinobacteriota_Actinobacteria_Micrococcales_Cellulomonadaceae_Cellulomonas_ASV620 |  | B_tax | 1 |
| Actinobacteriota_Actinobacteria_Micrococcales_Microbacteriaceae_Agromyces_ASV3 |  | B_tax | 1 |
| Actinobacteriota_Actinobacteria_Micrococcales_Microbacteriaceae_Microbacterium_ASV338 |  | B_tax | 1 |
| Actinobacteriota_Thermoleophilia_Gaiellales_Gaiellaceae_Gaiella_ASV155 |  | B_tax | 1 |
| Actinobacteriota_Thermoleophilia_Gaiellales_Gaiellaceae_Gaiella_ASV329 |  | B_tax | 1 |
| Actinobacteriota_Thermoleophilia_Gaiellales_Gaiellaceae_Gaiella_ASV571 |  | B_tax | 1 |
| Bacteroidota_Bacteroidia_Cytophagales_Microscillaceae_Ohtaekwangia_ASV1702 |  | B_tax | 1 |
| Chloroflexi_Chloroflexia_Thermomicrobiales_AKYG1722_Unclassified_ASV266 |  | B_tax | 1 |
| Chloroflexi_Chloroflexia_Thermomicrobiales_AKYG1722_Unclassified_ASV964 |  | B_tax | 1 |
| Chloroflexi_Chloroflexia_Thermomicrobiales_JG30-KF-CM45_Unclassified_ASV101 |  | B_tax | 1 |
| Chloroflexi_Chloroflexia_Thermomicrobiales_JG30-KF-CM45_Unclassified_ASV1081 |  | B_tax | 1 |
| Chloroflexi_Chloroflexia_Thermomicrobiales_JG30-KF-CM45_Unclassified_ASV1772 |  | B_tax | 1 |
| Chloroflexi_Chloroflexia_Thermomicrobiales_JG30-KF-CM45_Unclassified_ASV187 |  | B_tax | 1 |
| Chloroflexi_Chloroflexia_Thermomicrobiales_JG30-KF-CM45_Unclassified_ASV365 |  | B_tax | 1 |
| Chloroflexi_Chloroflexia_Thermomicrobiales_JG30-KF-CM45_Unclassified_ASV38 |  | B_tax | 1 |
| Chloroflexi_Chloroflexia_Thermomicrobiales_JG30-KF-CM45_Unclassified_ASV458 |  | B_tax | 1 |
| Chloroflexi_Chloroflexia_Thermomicrobiales_JG30-KF-CM45_Unclassified_ASV611 |  | B_tax | 1 |
| Chloroflexi_Chloroflexia_Thermomicrobiales_JG30-KF-CM45_Unclassified_ASV706 |  | B_tax | 1 |
| Chloroflexi_Chloroflexia_Thermomicrobiales_JG30-KF-CM45_Unclassified_ASV873 |  | B_tax | 1 |
| Chloroflexi_Chloroflexia_Thermomicrobiales_JG30-KF-CM45_Unclassified_ASV899 |  | B_tax | 1 |
| Proteobacteria_Alphaproteobacteria_Micropepsales_Micropepsaceae_Unclassified_ASV434 |  | B_tax | 1 |
| Proteobacteria_Gammaproteobacteria_Burkholderiales_Burkholderiaceae_Ralstonia_ASV299 |  | B_tax | 1 |
| Acidobacteriota_Vicinamibacteria_Vicinamibacterales_Vicinamibacteraceae_Unclassified_ASV387 |  | B_tax | 1 |
| Actinobacteriota_Actinobacteria_Propionibacteriales_Nocardioidaceae_Nocardioides_ASV2094 |  | B_tax | 1 |
| Actinobacteriota_Actinobacteria_Pseudonocardiales_Pseudonocardiaceae_Actinophytocola_ASV3416 |  | B_tax | 1 |
| Bacteroidota_Bacteroidia_Chitinophagales_Chitinophagaceae_Flaviaesturariibacter_ASV5688 |  | B_tax | 1 |
| Bacteroidota_Bacteroidia_Chitinophagales_Chitinophagaceae_Niastella_ASV4530 |  | B_tax | 1 |
| Firmicutes_Bacilli_Bacillales_Bacillaceae_Bacillus_ASV20 |  | B_tax | 1 |
| Firmicutes_Bacilli_Paenibacillales_Paenibacillaceae_Paenibacillus_ASV1729 |  | B_tax | 1 |
| Gemmatimonadota_Gemmatimonadetes_Gemmatimonadales_Gemmatimonadaceae_Gemmatimonas_ASV4320 |  | B_tax | 1 |
| Myxococcota_Myxococcia_Myxococcales_Myxococcaceae_Unclassified_ASV3115 |  | B_tax | 1 |
| Proteobacteria_Alphaproteobacteria_Rhizobiales_Beijerinckiaceae_Unclassified_ASV315 |  | B_tax | 1 |
| Proteobacteria_Gammaproteobacteria_Burkholderiales_Comamonadaceae_Ramlibacter_ASV1659 |  | B_tax | 1 |
| Proteobacteria_Gammaproteobacteria_Burkholderiales_Comamonadaceae_Rhizobacter_ASV1406 |  | B_tax | 1 |
| Proteobacteria_Gammaproteobacteria_Burkholderiales_Comamonadaceae_Unclassified_ASV2584 |  | B_tax | 1 |
| Proteobacteria_Gammaproteobacteria_Burkholderiales_Nitrosomonadaceae_Ellin6067_ASV3221 |  | B_tax | 1 |
| Proteobacteria_Gammaproteobacteria_Burkholderiales_Nitrosomonadaceae_Ellin6067_ASV350 |  | B_tax | 1 |
| Proteobacteria_Gammaproteobacteria_Burkholderiales_Oxalobacteraceae_Unclassified_ASV3687 |  | B_tax | 1 |
| Ca |  | Nutrients | 1 |
| Fe |  | Nutrients | 1 |
| C_total_ |  | Nutrients | 1 |
| Shoot_Dry_Mass |  | Plant-Growth | 1 |
| *dhbF_glyine*___[glycyl_carrier_protein]_ligase_[EC:6.2.1.66] |  | B_Genes | 1 |
| *fenD_fengycin* |  | B_Genes | 1 |
| *ituA_mycA_bmyA_iturin_family_lipopeptide_synthetase_A* |  | B_Genes | 1 |
| *ituB_mycB_bmyB_iturin_family_lipopeptide_synthetase_B* |  | B_Genes | 1 |
| *ituC_mycC_bmyC_iturin_family_lipopeptide_synthetase_C* |  | B_Genes | 1 |
| *entF_L_serine___[L_seryl_carrier_protein]_ligase_[EC:6.3.2.14_6.2.1.72]* |  | B_Genes | 1 |
| *ofaC_arfC_arthrofactin_type_cyclic_lipopeptide_synthetase_C* |  | B_Genes | 1 |
| *ofaB_arfB_arthrofactin_type_cyclic_lipopeptide_synthetase_B* |  | B_Genes | 1 |
| *Surfactin_srfAA* |  | B_Genes | 1 |
| *ofaA_arfA_arthrofactin_type_cyclic_lipopeptide_synthetase_A* |  | B_Genes | 1 |
| *Surfactin_srfAB* |  | B_Genes | 1 |
| *ZmNAS3* |  | P_Genes | 1 |
| *two_component_system_OmpR_family_response_regulator_QseB* |  | B_Genes | 1 |
| *Surfactin_srfAD* |  | B_Genes | 1 |
| *Surfactin_srfAC* |  | B_Genes | 1 |
| *bphB_cis_2,3_dihydrobiphenyl_2,3_diol_dehydrogenase_[EC:1.3.1.56]* |  | B_Genes | 1 |
| *Mn* |  | Nutrients | 1 |
| *mbtE_mycobactin_peptide_synthetase_MbtE* |  | B_Genes | 1 |
| CK |  | Shoot-Hormones | 1 |
| TRP1_anthranilate_synthase/indole_3_glycerol_phosphate_synthase/phosphoribosylanthranilate_isomerase_[EC:4.1.3.27_4.1.1.48_5.3.1.24] |  | B_Genes | 1 |
| GA |  | Shoot-Hormones | 1 |
| malic_acid |  | Metabolites | 1 |
| IAA |  | Shoot-Hormones | 1 |
| citric_acid |  | Metabolites | 1 |
| fumaric_acid |  | Metabolites | 1 |
| succinic_acid |  | Metabolites | 1 |
| cis_aconitic_acid |  | Metabolites | 1 |
| trans_aconitic_acid |  | Metabolites | 1 |
| Tryptophan |  | Metabolites | 1 |
| Asparagin |  | Metabolites | 1 |
| Serin |  | Metabolites | 1 |
| Glutamine |  | Metabolites | 1 |
| Glycin |  | Metabolites | 1 |
| Trehalose |  | Metabolites | 1 |
| Glucose |  | Metabolites | 1 |
| MBOA |  | Metabolites | 1 |
| Root-CK |  | Root-Hormones | 1 |
| Root-IAA |  | Root-Hormones | 1 |
| Root-JA |  | Root-Hormones | 1 |
| Benzoic_acid |  | Metabolites | 1 |
| Root-SA |  | Root-Hormones | 1 |
| ascorbate_peroxidase_activity |  | Stress-Factors | 1 |
| SOD |  | Stress-Factors | 1 |
| potA_spermidine_putrescine_transport_system_ATP_binding_protein |  | B_Genes | 1 |
| benzoxazin |  | Metabolites | 1 |
| benzoxazin4 |  | Metabolites | 1 |
| Quercetin_Naringenin |  | Metabolites | 1 |
| Glycine_betaine |  | Stress-Factors | 1 |
| total_antioxidants |  | Stress-Factors | 1 |
| Dichotomopilus;ASV8 |  | F_tax | 2 |
| Sporobolomyces;ASV15 |  | F_tax | 2 |
| Sporobolomyces;ASV15 |  | F_tax | 2 |
| Rhizophydiales_gen_Incertae_sedis;ASV68 |  | F_tax | 2 |
| Mortierella;ASV90 |  | F_tax | 2 |
| Mortierella;ASV91 |  | F_tax | 2 |
| Pyrenochaeta;ASV99 |  | F_tax | 2 |
| Pyrenochaeta;ASV99 |  | F_tax | 2 |
| Talaromyces;ASV149 |  | F_tax | 2 |
| Xylariales_gen_Incertae_sedis;ASV178 |  | F_tax | 2 |
| Preussia;ASV195 |  | F_tax | 2 |
| Gymnoascus;ASV198 |  | F_tax | 2 |
| Leucothecium;ASV205 |  | F_tax | 2 |
| Monographella;ASV276 |  | F_tax | 2 |
| Onygenales_gen_Incertae_sedis;ASV297 |  | F_tax | 2 |
| Thelebolaceae_gen_Incertae_sedis;ASV327 |  | F_tax | 2 |
| Thelebolaceae_gen_Incertae_sedis;ASV327 |  | F_tax | 2 |
| Cephalotrichiella;ASV342 |  | F_tax | 2 |
| Sordariales_gen_Incertae_sedis;ASV365 |  | F_tax | 2 |
| Sordariales_gen_Incertae_sedis;ASV365 |  | F_tax | 2 |
| Claroideoglomus;ASV370 |  | F_tax | 2 |
| Schizothecium;ASV382 |  | F_tax | 2 |
| Schizothecium;ASV382 |  | F_tax | 2 |
| Sordariales_gen_Incertae_sedis;ASV390 |  | F_tax | 2 |
| Sordariales_gen_Incertae_sedis;ASV390 |  | F_tax | 2 |
| Hypocreales_gen_Incertae_sedis;ASV396 |  | F_tax | 2 |
| Nectriaceae_gen_Incertae_sedis;ASV404 |  | F_tax | 2 |
| Nectriaceae_gen_Incertae_sedis;ASV404 |  | F_tax | 2 |
| Hypocreales_gen_Incertae_sedis;ASV412 |  | F_tax | 2 |
| Hypocreales_gen_Incertae_sedis;ASV412 |  | F_tax | 2 |
| Vishniacozyma;ASV525 |  | F_tax | 2 |
| Ramophialophora;ASV529 |  | F_tax | 2 |
| Ramophialophora;ASV529 |  | F_tax | 2 |
| Hypocreaceae_gen_Incertae_sedis;ASV535 |  | F_tax | 2 |
| Agaricomycetes_gen_Incertae_sedis;ASV552 |  | F_tax | 2 |
| Agaricomycetes_gen_Incertae_sedis;ASV552 |  | F_tax | 2 |
| Cheilymenia;ASV567 |  | F_tax | 2 |
| Cheilymenia;ASV567 |  | F_tax | 2 |
| Halosphaeriaceae_gen_Incertae_sedis;ASV579 |  | F_tax | 2 |
| Mucor;ASV625 |  | F_tax | 2 |
| Mucor;ASV625 |  | F_tax | 2 |
| Chytridiomycota_gen_Incertae_sedis;ASV659 |  | F_tax | 2 |
| Exophiala;ASV687 |  | F_tax | 2 |
| Stachybotryaceae_gen_Incertae_sedis;ASV693 |  | F_tax | 2 |
| Clonostachys;ASV713 |  | F_tax | 2 |
| Ambispora;ASV735 |  | F_tax | 2 |
| Fusarium;ASV740 |  | F_tax | 2 |
| Alternaria;ASV750 |  | F_tax | 2 |
| Alternaria;ASV750 |  | F_tax | 2 |
| Ophiosphaerella;ASV789 |  | F_tax | 2 |
| Ophiosphaerella;ASV789 |  | F_tax | 2 |
| Rhizophydiales_gen_Incertae_sedis;ASV820 |  | F_tax | 2 |
| Agaricomycetes_gen_Incertae_sedis;ASV824 |  | F_tax | 2 |
| Chrysozymaceae_gen_Incertae_sedis;ASV852 |  | F_tax | 2 |
| Chrysozymaceae_gen_Incertae_sedis;ASV852 |  | F_tax | 2 |
| Penicillium;ASV864 |  | F_tax | 2 |
| Penicillium;ASV864 |  | F_tax | 2 |
| Operculomyces;ASV933 |  | F_tax | 2 |
| Entoloma;ASV937 |  | F_tax | 2 |
| Entoloma;ASV937 |  | F_tax | 2 |
| Piptocephalis;ASV998 |  | F_tax | 2 |
| Piptocephalis;ASV998 |  | F_tax | 2 |
| Ascomycota_gen_Incertae_sedis;ASV1056 |  | F_tax | 2 |
| Ascomycota_gen_Incertae_sedis;ASV1056 |  | F_tax | 2 |
| Endogonomycetes_gen_Incertae_sedis;ASV1209 |  | F_tax | 2 |
| Endogonomycetes_gen_Incertae_sedis;ASV1209 |  | F_tax | 2 |
| Spizellomycetales_gen_Incertae_sedis;ASV1218 |  | F_tax | 2 |
| Samsoniella;ASV1225 |  | F_tax | 2 |
| Basidiomycota_gen_Incertae_sedis;ASV1314 |  | F_tax | 2 |
| Monosporascus;ASV1345 |  | F_tax | 2 |
| Monosporascus;ASV1345 |  | F_tax | 2 |
| Hypocreales_gen_Incertae_sedis;ASV1352 |  | F_tax | 2 |
| Hypocreales_gen_Incertae_sedis;ASV1352 |  | F_tax | 2 |
| Microbotryales_gen_Incertae_sedis;ASV1366 |  | F_tax | 2 |
| Pseudogymnoascus;ASV1450 |  | F_tax | 2 |
| Pseudogymnoascus;ASV1450 |  | F_tax | 2 |
| Mucor;ASV1465 |  | F_tax | 2 |
| Preussia;ASV1510 |  | F_tax | 2 |
| Pseudogymnoascus;ASV1530 |  | F_tax | 2 |
| Myxotrichum;ASV1583 |  | F_tax | 2 |
| Talaromyces;ASV1597 |  | F_tax | 2 |
| Botryotrichum;ASV7 |  | F_tax | 2 |
| Chaetomium;ASV10 |  | F_tax | 2 |
| Penicillium;ASV21 |  | F_tax | 2 |
| Acremonium;ASV33 |  | F_tax | 2 |
| Podila;ASV50 |  | F_tax | 2 |
| Halosphaeriaceae_gen_Incertae_sedis;ASV52 |  | F_tax | 2 |
| Rozellomycota_gen_Incertae_sedis;ASV73 |  | F_tax | 2 |
| Linnemannia;ASV85 |  | F_tax | 2 |
| Paraglomus;ASV125 |  | F_tax | 2 |
| Paraglomus;ASV127 |  | F_tax | 2 |
| Filobasidium;ASV153 |  | F_tax | 2 |
| Ascomycota_gen_Incertae_sedis;ASV232 |  | F_tax | 2 |
| Brunneochlamydosporium;ASV247 |  | F_tax | 2 |
| Lasiosphaeriaceae_gen_Incertae_sedis;ASV267 |  | F_tax | 2 |
| Ascomycota_gen_Incertae_sedis;ASV275 |  | F_tax | 2 |
| Fusidium;ASV306 |  | F_tax | 2 |
| Ascomycota_gen_Incertae_sedis;ASV312 |  | F_tax | 2 |
| Pseudeurotiaceae_gen_Incertae_sedis;ASV330 |  | F_tax | 2 |
| Pleosporales_gen_Incertae_sedis;ASV332 |  | F_tax | 2 |
| Lasiosphaeriaceae_gen_Incertae_sedis;ASV363 |  | F_tax | 2 |
| Sordariales_gen_Incertae_sedis;ASV380 |  | F_tax | 2 |
| Schizothecium;ASV386 |  | F_tax | 2 |
| Hypocreales_gen_Incertae_sedis;ASV407 |  | F_tax | 2 |
| Fusarium;ASV409 |  | F_tax | 2 |
| Dactylonectria;ASV422 |  | F_tax | 2 |
| Ascomycota_gen_Incertae_sedis;ASV477 |  | F_tax | 2 |
| Pleotrichocladium;ASV487 |  | F_tax | 2 |
| Papiliotrema;ASV496 |  | F_tax | 2 |
| Lipomyces;ASV512 |  | F_tax | 2 |
| Kiflimonium;ASV533 |  | F_tax | 2 |
| Hypocreales_gen_Incertae_sedis;ASV536 |  | F_tax | 2 |
| Mortierella;ASV602 |  | F_tax | 2 |
| Mortierella;ASV604 |  | F_tax | 2 |
| Mortierella;ASV605 |  | F_tax | 2 |
| Mortierella;ASV606 |  | F_tax | 2 |
| Stachybotrys;ASV692 |  | F_tax | 2 |
| Paramyrothecium;ASV699 |  | F_tax | 2 |
| Clonostachys;ASV715 |  | F_tax | 2 |
| Fusarium;ASV737 |  | F_tax | 2 |
| Nectriaceae_gen_Incertae_sedis;ASV742 |  | F_tax | 2 |
| Preussia;ASV748 |  | F_tax | 2 |
| Fusarium;ASV768 |  | F_tax | 2 |
| Pochonia;ASV815 |  | F_tax | 2 |
| Metacordyceps;ASV818 |  | F_tax | 2 |
| Basidiomycota_gen_Incertae_sedis;ASV918 |  | F_tax | 2 |
| Hypocreales_gen_Incertae_sedis;ASV968 |  | F_tax | 2 |
| Fungi_gen_Incertae_sedis;ASV1001 |  | F_tax | 2 |
| Pyrenochaeta;ASV1060 |  | F_tax | 2 |
| Psathyrella;ASV1079 |  | F_tax | 2 |
| Hyalorbilia;ASV1117 |  | F_tax | 2 |
| Spiromyces;ASV1120 |  | F_tax | 2 |
| Penicillium;ASV1150 |  | F_tax | 2 |
| Leucoagaricus;ASV1155 |  | F_tax | 2 |
| Udeniozyma;ASV1215 |  | F_tax | 2 |
| Akenomyces;ASV1242 |  | F_tax | 2 |
| Mortierella;ASV1304 |  | F_tax | 2 |
| Ascomycota_gen_Incertae_sedis;ASV1306 |  | F_tax | 2 |
| Marquandomyces;ASV1309 |  | F_tax | 2 |
| Ascomycota_gen_Incertae_sedis;ASV1311 |  | F_tax | 2 |
| Ascomycota_gen_Incertae_sedis;ASV1353 |  | F_tax | 2 |
| Arachnomyces;ASV1363 |  | F_tax | 2 |
| Alternaria;ASV1383 |  | F_tax | 2 |
| Mortierella;ASV1388 |  | F_tax | 2 |
| Mortierella;ASV1389 |  | F_tax | 2 |
| Agaricomycetes_gen_Incertae_sedis;ASV1427 |  | F_tax | 2 |
| Filobasidium;ASV1440 |  | F_tax | 2 |
| Gaeumannomyces;ASV1443 |  | F_tax | 2 |
| Trichoderma;ASV1533 |  | F_tax | 2 |
| Trichoderma;ASV1537 |  | F_tax | 2 |
| Cephalotrichum;ASV1557 |  | F_tax | 2 |
| Fusarium;ASV1568 |  | F_tax | 2 |
| Fusicolla;ASV1584 |  | F_tax | 2 |
| Trichocladium;ASV1585 |  | F_tax | 2 |
| Talaromyces;ASV1593 |  | F_tax | 2 |
| Actinobacteriota_Acidimicrobiia_Microtrichales_Ilumatobacteraceae_Ilumatobacter_ASV74 |  | B_tax | 2 |
| Actinobacteriota_Acidimicrobiia_Microtrichales_Ilumatobacteraceae_Unclassified_ASV110 |  | B_tax | 2 |
| Actinobacteriota_Acidimicrobiia_Microtrichales_Ilumatobacteraceae_Unclassified_ASV1274 |  | B_tax | 2 |
| Actinobacteriota_Acidimicrobiia_Microtrichales_Ilumatobacteraceae_Unclassified_ASV233 |  | B_tax | 2 |
| Actinobacteriota_Acidimicrobiia_Microtrichales_Ilumatobacteraceae_Unclassified_ASV316 |  | B_tax | 2 |
| Actinobacteriota_Actinobacteria_Corynebacteriales_Nocardiaceae_Nocardia_ASV206 |  | B_tax | 2 |
| Actinobacteriota_Actinobacteria_Frankiales_Geodermatophilaceae_Unclassified_ASV3487 |  | B_tax | 2 |
| Actinobacteriota_Actinobacteria_Micromonosporales_Micromonosporaceae_Catellatospora_ASV165 |  | B_tax | 2 |
| Actinobacteriota_Actinobacteria_Propionibacteriales_Nocardioidaceae_Actinopolymorpha_ASV1319 |  | B_tax | 2 |
| Actinobacteriota_Actinobacteria_Propionibacteriales_Nocardioidaceae_Kribbella_ASV134 |  | B_tax | 2 |
| Actinobacteriota_Actinobacteria_Propionibacteriales_Nocardioidaceae_Kribbella_ASV327 |  | B_tax | 2 |
| Actinobacteriota_Actinobacteria_Propionibacteriales_Nocardioidaceae_Kribbella_ASV372 |  | B_tax | 2 |
| Actinobacteriota_Actinobacteria_Propionibacteriales_Nocardioidaceae_Nocardioides_ASV192 |  | B_tax | 2 |
| Actinobacteriota_Actinobacteria_Propionibacteriales_Nocardioidaceae_Nocardioides_ASV252 |  | B_tax | 2 |
| Actinobacteriota_Actinobacteria_Propionibacteriales_Nocardioidaceae_Nocardioides_ASV383 |  | B_tax | 2 |
| Actinobacteriota_Actinobacteria_Propionibacteriales_Nocardioidaceae_Nocardioides_ASV475 |  | B_tax | 2 |
| Actinobacteriota_Actinobacteria_Propionibacteriales_Nocardioidaceae_Nocardioides_ASV573 |  | B_tax | 2 |
| Actinobacteriota_Actinobacteria_Streptomycetales_Streptomycetaceae_Streptomyces_ASV25 |  | B_tax | 2 |
| Actinobacteriota_Actinobacteria_Streptomycetales_Streptomycetaceae_Streptomyces_ASV27 |  | B_tax | 2 |
| Actinobacteriota_Actinobacteria_Streptomycetales_Streptomycetaceae_Streptomyces_ASV58 |  | B_tax | 2 |
| Actinobacteriota_Actinobacteria_Streptomycetales_Streptomycetaceae_Streptomyces_ASV78 |  | B_tax | 2 |
| Actinobacteriota_Actinobacteria_Streptomycetales_Streptomycetaceae_Unclassified_ASV149 |  | B_tax | 2 |
| Actinobacteriota_Rubrobacteria_Rubrobacterales_Rubrobacteriaceae_Rubrobacter_ASV112 |  | B_tax | 2 |
| Actinobacteriota_Thermoleophilia_Gaiellales_Gaiellaceae_Gaiella_ASV556 |  | B_tax | 2 |
| Actinobacteriota_Thermoleophilia_Solirubrobacterales_67-14_Unclassified_ASV531 |  | B_tax | 2 |
| Firmicutes_Bacilli_Bacillales_Bacillaceae_Unclassified_ASV82 |  | B_tax | 2 |
| Gemmatimonadota_Gemmatimonadetes_Gemmatimonadales_Gemmatimonadaceae_Unclassified_ASV103 |  | B_tax | 2 |
| Gemmatimonadota_Gemmatimonadetes_Gemmatimonadales_Gemmatimonadaceae_Unclassified_ASV126 |  | B_tax | 2 |
| Gemmatimonadota_Gemmatimonadetes_Gemmatimonadales_Gemmatimonadaceae_Unclassified_ASV54 |  | B_tax | 2 |
| Proteobacteria_Alphaproteobacteria_Rhizobiales_Rhizobiaceae_Neorhizobium_ASV685 |  | B_tax | 2 |
| Proteobacteria_Alphaproteobacteria_Rhizobiales_Rhizobiaceae_Phyllobacterium_ASV34 |  | B_tax | 2 |
| Proteobacteria_Alphaproteobacteria_Rhizobiales_Rhizobiaceae_Unclassified_ASV137 |  | B_tax | 2 |
| Proteobacteria_Alphaproteobacteria_Sphingomonadales_Sphingomonadaceae_Sphingomonas_ASV1005 |  | B_tax | 2 |
| Proteobacteria_Alphaproteobacteria_Sphingomonadales_Sphingomonadaceae_Sphingomonas_ASV460 |  | B_tax | 2 |
| Proteobacteria_Gammaproteobacteria_Burkholderiales_Comamonadaceae_Ramlibacter_ASV987 |  | B_tax | 2 |
| Proteobacteria_Gammaproteobacteria_Xanthomonadales_Xanthomonadaceae_Lysobacter_ASV839 |  | B_tax | 2 |
| Verrucomicrobiota_Verrucomicrobiae_Verrucomicrobiales_Rubritaleaceae_Luteolibacter_ASV1997 |  | B_tax | 2 |
| Verrucomicrobiota_Verrucomicrobiae_Verrucomicrobiales_Rubritaleaceae_Luteolibacter_ASV3626 |  | B_tax | 2 |
| Actinobacteriota_Actinobacteria_Micrococcales_Microbacteriaceae_Microbacterium_ASV228 |  | B_tax | 2 |
| Actinobacteriota_Actinobacteria_Micrococcales_Micrococcaceae_Pseudarthrobacter_ASV18 |  | B_tax | 2 |
| Actinobacteriota_Actinobacteria_Micrococcales_Micrococcaceae_Pseudarthrobacter_ASV2 |  | B_tax | 2 |
| Actinobacteriota_Actinobacteria_Micrococcales_Micrococcaceae_Pseudarthrobacter_ASV5 |  | B_tax | 2 |
| Actinobacteriota_Actinobacteria_Micrococcales_Micrococcaceae_Unclassified_ASV24 |  | B_tax | 2 |
| Actinobacteriota_Actinobacteria_Propionibacteriales_Nocardioidaceae_Nocardioides_ASV170 |  | B_tax | 2 |
| Actinobacteriota_Actinobacteria_Streptomycetales_Streptomycetaceae_Streptomyces_ASV131 |  | B_tax | 2 |
| Actinobacteriota_Actinobacteria_Streptomycetales_Streptomycetaceae_Streptomyces_ASV53 |  | B_tax | 2 |
| Actinobacteriota_Thermoleophilia_Solirubrobacterales_67-14_Unclassified_ASV1824 |  | B_tax | 2 |
| Actinobacteriota_Thermoleophilia_Solirubrobacterales_67-14_Unclassified_ASV237 |  | B_tax | 2 |
| Actinobacteriota_Thermoleophilia_Solirubrobacterales_67-14_Unclassified_ASV264 |  | B_tax | 2 |
| Actinobacteriota_Thermoleophilia_Solirubrobacterales_67-14_Unclassified_ASV49 |  | B_tax | 2 |
| Actinobacteriota_Thermoleophilia_Solirubrobacterales_Solirubrobacteraceae_Conexibacter_ASV472 |  | B_tax | 2 |
| Actinobacteriota_Thermoleophilia_Solirubrobacterales_Solirubrobacteraceae_Solirubrobacter_ASV2951 |  | B_tax | 2 |
| Actinobacteriota_Thermoleophilia_Solirubrobacterales_Solirubrobacteraceae_Unclassified_ASV4027 |  | B_tax | 2 |
| Proteobacteria_Alphaproteobacteria_Rhizobiales_Beijerinckiaceae_Microvirga_ASV216 |  | B_tax | 2 |
| Proteobacteria_Alphaproteobacteria_Rhizobiales_Beijerinckiaceae_Microvirga_ASV240 |  | B_tax | 2 |
| Proteobacteria_Alphaproteobacteria_Rhizobiales_Beijerinckiaceae_Microvirga_ASV48 |  | B_tax | 2 |
| Proteobacteria_Alphaproteobacteria_Rhizobiales_Beijerinckiaceae_Microvirga_ASV51 |  | B_tax | 2 |
| Proteobacteria_Alphaproteobacteria_Rhizobiales_Beijerinckiaceae_Psychroglaciecola_ASV360 |  | B_tax | 2 |
| Proteobacteria_Alphaproteobacteria_Rhizobiales_Methyloligellaceae_Unclassified_ASV256 |  | B_tax | 2 |
| Proteobacteria_Alphaproteobacteria_Rhizobiales_Rhizobiaceae_Unclassified_ASV514 |  | B_tax | 2 |
| Proteobacteria_Alphaproteobacteria_Sphingomonadales_Sphingomonadaceae_Sphingomonas_ASV140 |  | B_tax | 2 |
| Proteobacteria_Alphaproteobacteria_Sphingomonadales_Sphingomonadaceae_Sphingomonas_ASV157 |  | B_tax | 2 |
| Proteobacteria_Alphaproteobacteria_Sphingomonadales_Sphingomonadaceae_Sphingomonas_ASV214 |  | B_tax | 2 |
| Proteobacteria_Alphaproteobacteria_Sphingomonadales_Sphingomonadaceae_Sphingomonas_ASV56 |  | B_tax | 2 |
| Proteobacteria_Gammaproteobacteria_Burkholderiales_Comamonadaceae_Variovorax_ASV894 |  | B_tax | 2 |
| Proteobacteria_Gammaproteobacteria_Burkholderiales_SC-I-84_Unclassified_ASV1129 |  | B_tax | 2 |
| Proteobacteria_Gammaproteobacteria_Pseudomonadales_Pseudomonadaceae_Pseudomonas_ASV1083 |  | B_tax | 2 |
| Proteobacteria_Gammaproteobacteria_Xanthomonadales_Rhodanobacteraceae_Unclassified_ASV448 |  | B_tax | 2 |
| *ZmPR1* |  | P_Genes | 2 |
| *ZmPR4* |  | P_Genes | 2 |
| *ZmACS6* |  | P_Genes | 2 |
| *ZmDef1* |  | P_Genes | 2 |
| *ZmDef2* |  | P_Genes | 2 |
| *ZmWRKY17* |  | P_Genes | 2 |
| *ZmWRKY33* |  | P_Genes | 2 |
| *ZmMPK3* |  | P_Genes | 2 |
| *ZmWRKY58* |  | P_Genes | 2 |
| *ZmERF1* |  | P_Genes | 2 |
| *ZmEREB58* |  | P_Genes | 2 |
| *ZmIRTa* |  | P_Genes | 2 |
| *NR1* |  | P_Genes | 2 |
| *NAR2.2* |  | P_Genes | 2 |
| *ZmMYB30* |  | P_Genes | 2 |
| *ZmMYB36* |  | P_Genes | 2 |
| *ZmWRKY106* |  | P_Genes | 2 |
| *ZmMYB95* |  | P_Genes | 2 |
| *ZmPht3* |  | P_Genes | 2 |
| *ZmPht4* |  | P_Genes | 2 |
| *potD_spermidine_putrescine_transport_system_substrate_binding_protein* |  | B_Genes | 2 |
| *4,5_dihydroxyphthalate_decarboxylase_[EC:4.1.1.55]* |  | B_Genes | 2 |
| *ZmPht8* |  | P_Genes | 2 |
| *ABA* |  | Shoot-Hormones | 2 |
| *JA* |  | Shoot-Hormones | 2 |
| *mbtF_mycobactin_peptide_synthetase_MbtF* |  | B_Genes | 2 |
| *rbsB_ribose_transport_system_substrate_binding_protein_quorumSensing* |  | B_Genes | 2 |
| H_2_O_2_ |  | Stress-Factors | 2 |

**Table S10**. HPLC conditions for determination of phenolic compounds in rhizosphere soil solutions of maize (cv. Benedictio).

| **UHPLC-MS** (identification) | **RP-HPLC** (quantitative analysis with known standards) |
| --- | --- |
| HPLC System: Agilent 1290 Ultra Performance Liquid Chromatography System with QExactive Plus Electrospray Mass Spectrometer (Thermo Fisher Scientific) | HPLC System: Shimadzu LC10 |
| Column: Phenomenex Synergi 4µ Polar-RP 80A 250 x 2 mm (Phenomenex, Torrance, CA, United States) | Column: GROM-SIL 120 ODS ST, 5 μm, 290 × 4.6 mm + 20 × 4.6 mm guard column (Grom, Herrenberg, Germany) |
| Eluent: 0.2% formic acid (solvent A);  0.2% formic acid in acetonitrile (solvent B) | Eluent: 0.2% formic acid (solvent A); methanol (solvent B) |
| Gradient elution: 0 - 6.0 min: 4 -18% B; 6.0 - 18 min: 18 - 26% B; 18 - 25 min: 26 - 65% B; 25 - 36 min: 65 - 100% B; 36 - 40 min: 100 % B. | Gradient elution: 0 - 32 min: 25 - 50% B; 32 - 33 min: 50 - 55% B; 33 - 42 min: 55% B; 42 - 43 min: 55 - 100% B; 43 - 46 min: 100% B. |
| Flow rate: 0.4 ml min^-1^ | Flow rate: 0.4 ml min^-1^ |
| Injection volume: 20 µl | Injection volume: 20 µl |
| Column temperature: 40°C | Column temperature: 40°C |
| Detection: MS parameters: ESI ddMS2 (TOP5) positive/negative, 100 - 950 µ, Spray Voltage 5 kV, capillary temp 360°C, sheath gas 60, Aux gas 20, probe heater 450°C with resolution full scan mode: 70,000, Resolution MS2 Spectra 17.500 and fragmentation by stepped collision energy: 20,60,110. | Detection: UV 280 nm |
